# Lewis Acid Assisted Brønsted Acid Catalysed Decarbonylation of Isocyanates: A Combined DFT and Experimental Study

**DOI:** 10.1002/chem.202201422

**Published:** 2022-06-21

**Authors:** Ayan Dasgupta, Yara van Ingen, Michael G. Guerzoni, Kaveh Farshadfar, Jeremy M. Rawson, Emma Richards, Alireza Ariafard, Rebecca L. Melen

**Affiliations:** ^1^ Cardiff Catalysis Institute School of Chemistry Cardiff University main Building Park Place Cardiff CF10 3AT Cymru/Wales United Kingdom; ^2^ Department of Chemistry Islamic Azad University Central TehranBranch, Poonak Tehran 1469669191 Iran; ^3^ Department of Chemistry and Biochemistry University of Windsor 401 Sunset Ave. Windsor ON N9B 3P4 Canada; ^4^ School of Natural Sciences-Chemistry University of Tasmania Private Bag 75 Hobart Tasmania 7001 Australia

**Keywords:** biuret, boranes, DFT, isocyanate, urea

## Abstract

An efficient and mild reaction protocol for the decarbonylation of isocyanates has been developed using catalytic amounts of Lewis acidic boranes. The electronic nature (electron withdrawing, electron neutral, and electron donating) and the position of the substituents (*ortho*/*meta*/*para*) bound to isocyanate controls the chain length and composition of the products formed in the reaction. Detailed DFT studies were undertaken to account for the formation of the mono/di‐carboxamidation products and benzoxazolone compounds.

## Graphical abstract

**An efficient and mild reaction protocol** for the decarbonylation of isocyanates using catalytic amounts of Lewis acidic boranes is described. The electronic nature and the position of the substituents (*ortho*/*meta*/*para*) bound to isocyanate controls the chain length and composition of the products.

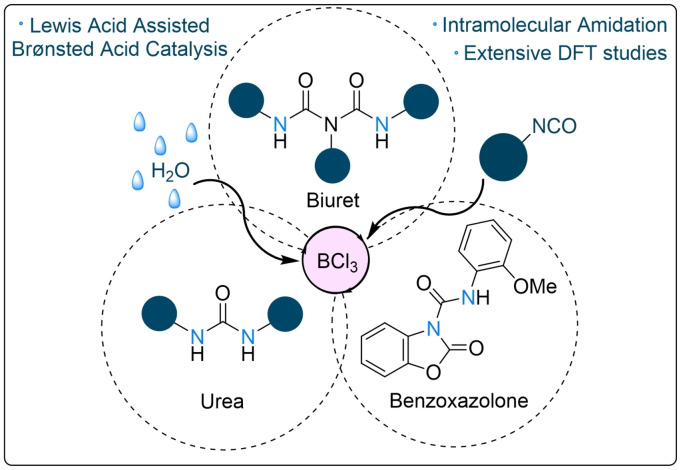

Studies on the application of main group elements in synthetic chemistry has recently become a burgeoning field of research.[^1^, ^2^, ^3^] The catalytic utility of main group elements has received unprecedented attention from the scientific community and extensive studies have revealed many previously unidentified reactivities of such elements.^[4]^ In particular, boranes have demonstrated outstanding reactivities and promising outcomes in catalysing a range of organic reactions.^[5]^ The presence of an empty *p*‐orbital at the central boron atom renders them catalytically active as they can readily, but reversibly, accept a pair of electrons from donor substrates.^[6]^ Boranes are oxophilic and can readily form an adduct with a water molecule to act as a Brønsted acid. The ability of the borane‐water adduct to donate a proton is as strong as HCl (p*K_a_* =8.4/8.5 in MeCN).^[7]^

Isocyanates are known to be reactive electrophiles^[8]^ which undergo a range of reactions with various nucleophiles, such as amines to produce carbodiimides,[^9^, ^10^, ^11^] as exemplified by the hydroamination of isocyanates which produces functionalised urea derivatives.[^12^, ^13^, ^14^] This class of scaffolds has been identified as important building blocks towards pharmaceuticals,^[15]^ agrochemicals,^[16]^ and in materials chemistry.^[17]^ Following a report by Perveen et al.,^[18]^ Gale and co‐workers demonstrated the coupling of aromatic amines with aryl isocyanates to afford symmetrical urea derivatives, using excess or stoichiometric amounts of a tertiary amine base.^[19]^ In 2019, Kays and co‐workers reported iron(II)‐catalysed hydroamination of isocyanates in which the reaction between aryl/alkyl secondary amines with aryl/alkyl isocyanates afforded biuret products (Scheme 1, bottom, left).^[20]^ Recent studies from Wang and co‐workers revealed that (un)symmetrical biuret derivatives can be synthesised from the reaction between various aryl/alkyl isocyanates and secondary amines without the use of a catalyst/solvent.^[21]^ Relating to this work, in 2019 Goicoechea and co‐workers investigated 1,2‐carboboration of the isocyanate C=O bond employing stoichiometric amounts of isocyanates and electrophilic tris(pentafluorophenyl)borane [B(C_6_F_5_)_3_] to afford six‐membered heterocycles.^[22]^

**Scheme 1:**
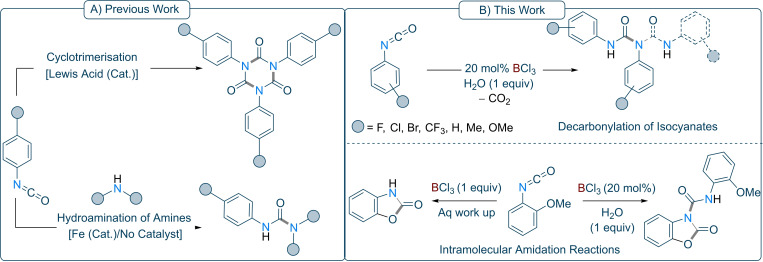
(A) Cyclotrimerisation and hydroamination of amines using aryl/alkyl isocyanates; (B) Borane catalysed decarbonylation and intramolecular cyclisation of aryl/alkyl isocyanates.

However, reactivities of catalytic Lewis acidic boranes towards isocyanates remain unexplored. In 2019, Ward and co‐workers demonstrated a synthetic methodology to afford cyclic trimers from isocyanates and di‐isocyanates using catalytic amounts of Lewis acidic Al‐complexes in good to excellent yields (17 examples, yields up to 98 %) (Scheme 1, top left).^[23]^ In this study, we were interested in the catalytic applications of boranes for the formation of mono/di‐carboxamidation products and oxazolone scaffolds (Scheme 1, right).

We began this study with the catalytic reaction of B(C_6_F_5_)_3_ (10 mol %) with phenyl isocyanate in 1,2‐dichloroethane (1,2‐C_2_H_4_Cl_2_) at room temperature (23 °C). After 24 h the reaction mixture was quenched with saturated NH_4_Cl (aq.) solution. Slow evaporation of the resulting reaction mixture from dichloromethane (CH_2_Cl_2_) led to the formation of colourless crystals whose molecular structure was determined from single crystal X‐ray diffraction to be a symmetrical biuret derivative *N*,*N*′,*N*′′‐triphenylbiuret (**18**) (See Supporting Information, Figure S40).^[24]^ The product resulted from the trimerisation of the isocyanate with loss of CO, an analogous structure (**20**) was obtained when employing *p*‐Cl phenyl isocyanate as depicted in Figure 1 (left). We then turned our attention to establish a general method to prepare such biuret compounds using catalytic amounts of a borane catalyst. To establish the optimal reaction conditions to afford the biuret product **18**, phenyl isocyanate was treated with 5, 10, and 20 mol % of B(C_6_F_5_)_3_ at both 23 °C and 70 °C in 1,2‐C_2_H_4_Cl_2_. Unfortunately, **18** was obtained only in poor yields in all cases (15–22 %). The more Lewis acidic BCl_3_ ^[25]^ was also tested, however, 20 mol % catalytic loading in 1,2‐C_2_H_4_Cl_2_ at 70 °C transformed phenyl isocyanate to **18** in just 20 % isolated yield. Interestingly, the 1 : 1 stoichiometric reaction led to the formation of the six‐membered borane adduct **20 a** in 56 % yield (Figure 1, right).^[26]^

**Figure 1:**
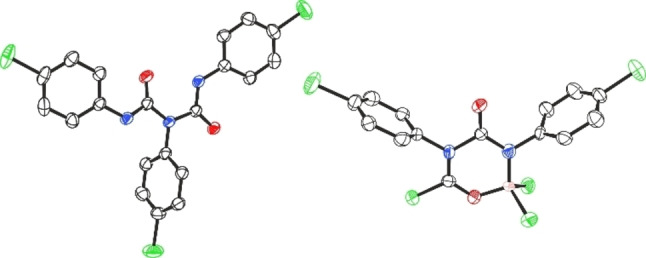
Crystal structures of **20** (left) and **20 a** (right). Thermal ellipsoids shown at 50 %. H atoms omitted for clarity. Carbon: black; Oxygen: red; Nitrogen: blue; Chlorine: green; Boron: pink.

The formation of **18** from phenyl isocyanate using catalytic amounts of borane raised questions on the loss of one CO unit from phenyl isocyanate to afford the corresponding biuret, and also on the source of protons to account for the N−H groups in the product. Control experiments were performed to investigate the source of protons. Phenyl isocyanate was treated with 20 mol % BCl_3_ in 1,2‐C_2_H_4_Cl_2_ at 70 °C. After 20 h, the reaction was quenched with 2 mL H_2_O and the biuret product **18** was isolated in 35 % yield. Furthermore, if stoichiometric amounts of H_2_O were deliberately introduced to the 1,2‐C_2_H_4_Cl_2_ solvent, the yield of **18** was increased to 71 %. Therefore, it can be unequivocally concluded that the source of protons in the products is due to trace water present in the reaction.

Although BCl_3_ is prone to hydrolysis to afford H_3_BO_3_, we analysed the reaction using 20 mol % H_3_BO_3_ as a catalyst and the decarbonylation of phenyl isocyanate was not observed. This suggests that, under the reaction conditions, stoichiometric water does not react with BCl_3_ to form boric acid, rather a H_2_O→BCl_3_ (**5**) adduct forms, which most likely acts as a Brønsted acid catalyst for this reaction.

These results motivated us to establish the most plausible reaction pathway for the biuret synthesis. We undertook DFT calculations at the SMD/M06‐2X‐D3/def2‐TZVP//SMD/M06‐2X/6‐31G(d) level of theory in CH_2_Cl_2_ to unveil the reaction mechanism. As shown in Figure 2a, the activation of phenyl isocyanate using a Lewis acidic borane can take place through two different modes: direct activation (path A); or Lewis acid assisted Brønsted acid activation (LBA, path B). In path A, BCl_3_ binds to phenyl isocyanate **2** and significantly increases the electrophilicity of the carbonyl carbon, thereby facilitating the nucleophilic attack of another phenyl isocyanate/water molecule to afford the desired biuret product. Either the oxygen or nitrogen functionality of phenyl isocyanate can coordinate to BCl_3_ to afford intermediates **4** and **3** which are 5.6 and 2.1 kcal/mol higher in energy than the reference structure **1**, respectively (Figure 2a). Our calculations indicate that H_2_O acts as a better nucleophile than phenyl isocyanate in attacking the activated isocyanate, a statement supported by the fact that the transition structures associated with the nucleophilic attack of water via **TS^ii^**_**3**_/**TS^ii^**_**4**_ are positioned lower in energy than those associated with the nucleophilic attack of a free isocyanate via **TS^i^**_**3**_/**TS^i^**_**4**_. We also found that the energy barrier for attack by the nucleophile to **3** (N coordination) is lower than that to **4** (O coordination); for example, the nucleophilic attack of water via **TS^ii^**_**3**_ is 5.2 kcal/mol lower in energy than that via **TS^ii^**_**4**_. Thus, better activation of the isocyanate occurs when BCl_3_ coordinates to the nitrogen atom. In the LBA mode (path B), a water molecule coordinates to BCl_3_ generating H_2_O→BCl_3_ (**5**) which can act as a Brønsted acid. Once **5** is formed, it can activate the phenyl isocyanate through the formation of intermediate **6** in which the H_2_O→BCl_3_ adduct interacts with the nitrogen atom of the isocyanate. Following that, the in situ generated anion [BCl_3_(OH)]^−^ acts as the nucleophile, attacking the carbonyl carbon of the activated isocyanate via transition structure **TS_6‐7_**, forming intermediate **7**. Our calculations show that path B (LBA mechanism) is favoured over path A (direct activation), as evidenced by **TS_6‐7_** having lower energy than all transition states in path A. As a result, the rest of our DFT investigations will concentrate exclusively on the details of the LBA mechanism. As shown in Figure 2a, once intermediate **7** has formed it is then isomerised to the more stable intermediate **8**, after overcoming an overall activation barrier of 6.1 kcal/mol. The isomerisation of **8** to the less stable species **9** via BCl_3_ migration from the oxygen to the nitrogen atom sets the stage for CO_2_ liberation. We found that water can mediate CO_2_ liberation via a deprotonation process involving transition structure **TS_10‐11_**, directly leading to the formation of an aniline coordinated to BCl_3_ (species **11**). The reaction between **11** and water rapidly leads to salt **12**. This subsequently dissociates to produce aniline and regenerate **5**, thus completing catalytic cycle 1. It follows from the above discussion that cycle 1 generates aniline and releases the active catalyst H_2_O→BCl_3_ (**5**) from **12** in an endergonic process with ΔG=15.8 kcal/mol.

**Figure 2:**
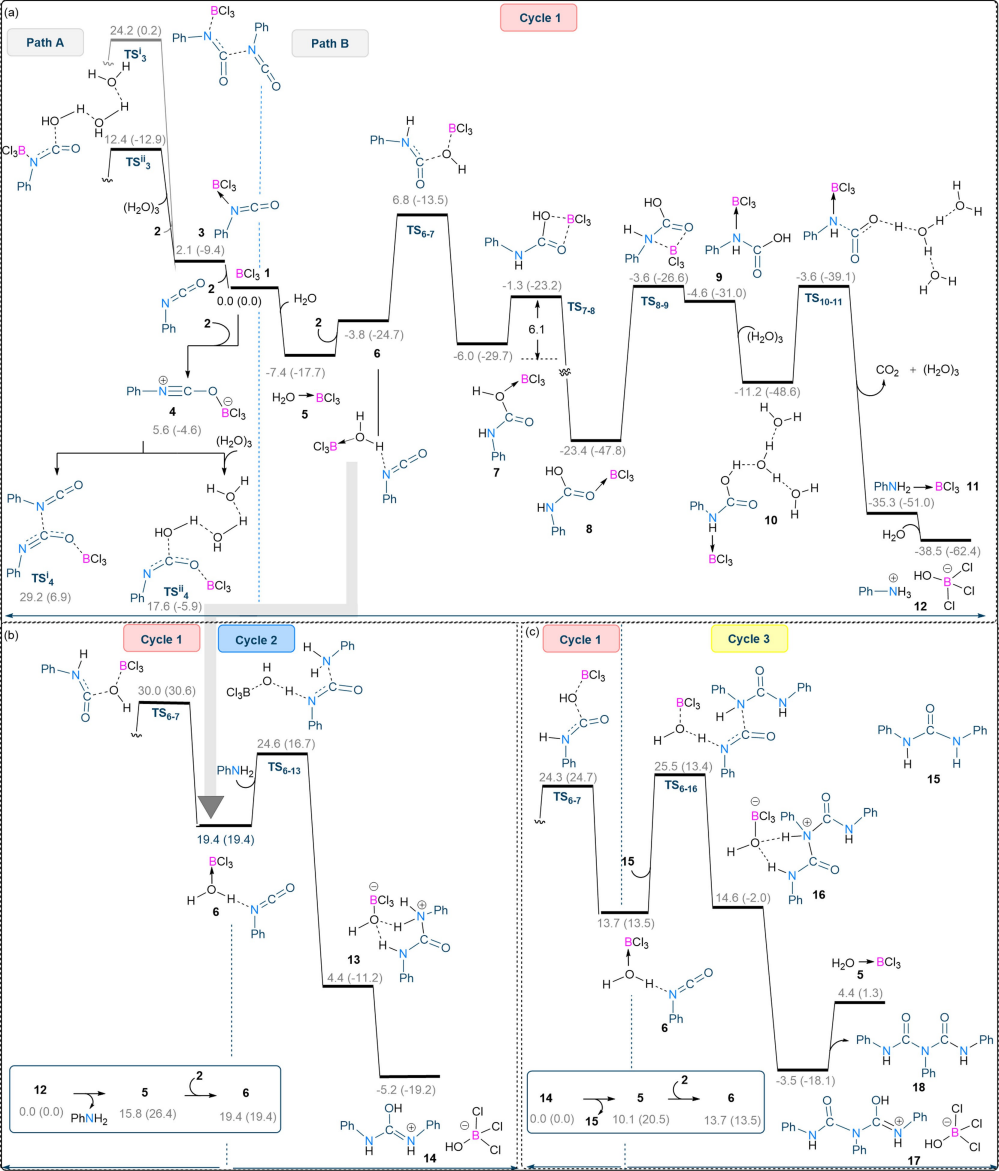
DFT computed reaction pathways calculated using SMD/M06‐2X‐D3/def2‐TZVP//SMD/M06‐2X/6‐31G(d) level of theory in dichloroethane for the formation of decarbonylative trimerisation of phenyl isocyanate using BCl_3_ as a catalyst. (a) Comparison between the energy profiles for Lewis acid catalysis (path A) and LBA catalysis (path B) for formation of aniline (cycle 1). (b) Comparison between the energy profiles for cycles 1 and 2. Since intermediate **12** is the most stable species formed in cycle 1, it was chosen as the reference structure for this comparison. (c) Comparison between the energy profiles for cycles 1 and 3. Since intermediate **14** is the most stable species formed in cycle 2, it was chosen as the reference structure for this comparison. Free energies (potential energies) are given in kcal/mol.

The synthesis of aniline from phenyl isocyanate using catalytic amounts of Lewis acids was patented in 1991.^[27]^ We also generated aniline from phenyl isocyanate with stoichiometric BCl_3_ and water experimentally in 1,2‐CH_2_Cl_2_ at 70 °C for 18 h. A basic work‐up of the reaction mixture with 1 M NaOH led to the formation of aniline, as evidenced by the crude ^1^H NMR spectrum (see Supporting Information, Figure S39).

Once aniline has formed catalytic cycle 2 can now occur. As before, active catalyst **5** can react with an isocyanate to form **6**, which is a junction for the two cycles either (i) attack by water to give another molecule of aniline (cycle 1), or (ii) attack by aniline to yield urea product **15** (cycle 2) (Figure 2). Our calculations explicitly predict that catalytic cycle 2 occurs more rapidly than catalytic cycle 1, as demonstrated by the fact that **TS_6‐13_** has lower energy than **TS_6‐7_**. As shown in Figure 2b, the aniline generated in cycle 1 reacts with **6** to afford species **13** after crossing transition structure **TS_6‐13_**. A proton shift from nitrogen to oxygen in **13** produces the stable ion pair **14**. Dissociation of **14** to the urea product **15** and regeneration of the active catalyst **5** is an endergonic process with ΔG=10.1 kcal/mol (Figure 2c, insert).

Following the generation of the urea product in cycle 2, the active catalyst again reacts with another isocyanate to produce intermediate **6**. Once formed, cycle 1 and 2 can now compete with cycle 3. In cycle 3, urea **15** acts as the nucleophile and produces the final biuret product **18**. Since **15** is a weaker nucleophile than aniline, cycle 3 is calculated to proceed at a rate comparable to cycle 1, as evidenced by the close energies of **TS_6‐7_** and **TS_6‐16_** (Figure 2). This result explains why the formation of the biuret product is highly dependent on the identity of the isocyanate used (see below); the urea products with a weaker nucleophilic property do not form a biuret. However, in the case shown in Figure 2c, **TS_6‐16_** is expected to have lower energy than **TS_6‐7_**. This inconsistency can be explained by an error in the overestimation of the entropy effect for **TS_6‐16_**, which involves two molecules **6** and **15** to produce this transition structure. It is well established that all two‐to‐one transformations suffer from such a calculation error.^[28]^ This type of error does not exist for **TS_6‐7_** because it is formed via a one‐to‐one transformation. The proposed mechanism of the three concurrent catalytic cycles is shown in Figure 3.

**Figure 3:**
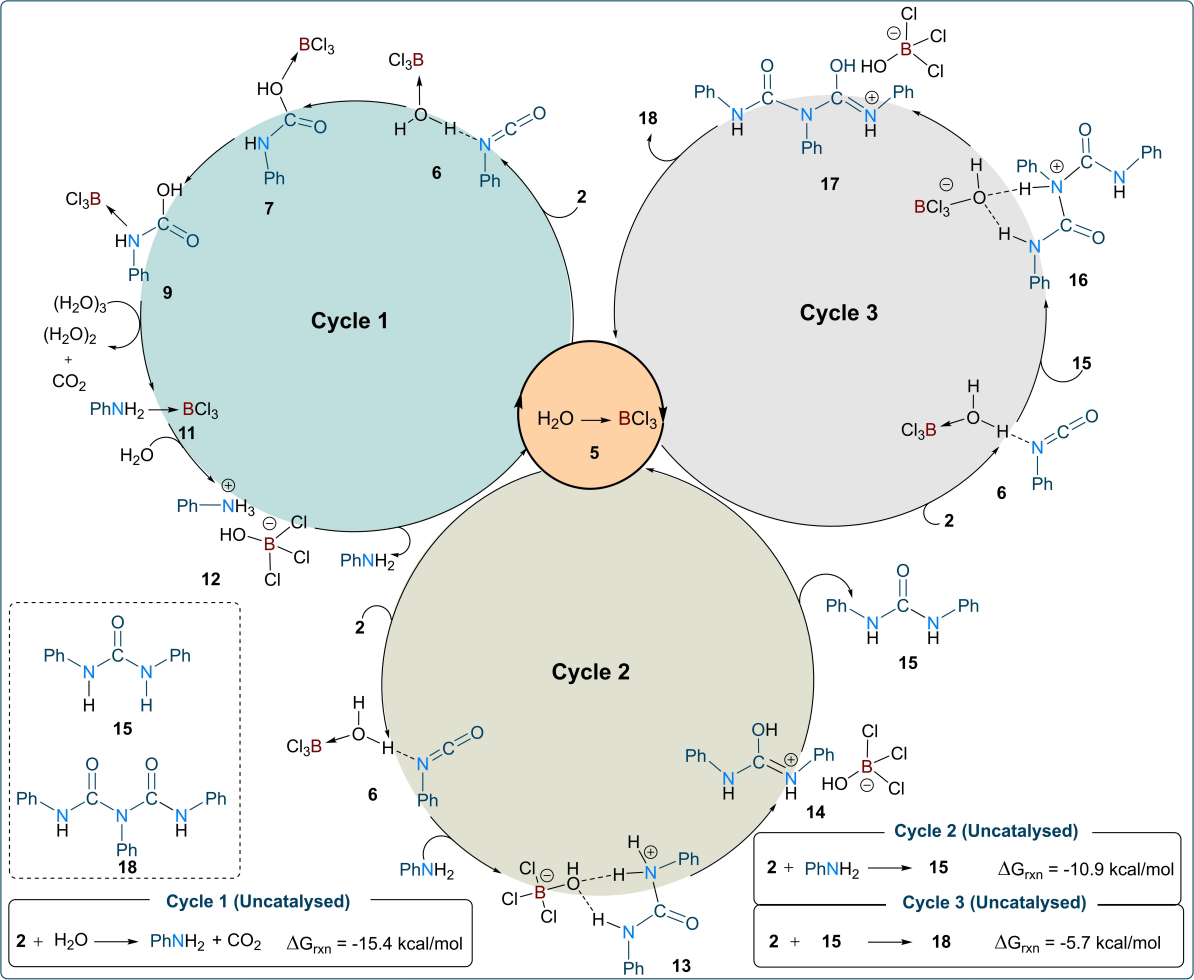
DFT‐based proposed reaction mechanism for the formation of biuret products from phenyl isocyanate using catalytic BCl_3_.

We have also investigated the thermodynamic aspects of the formation of aniline, urea **15**, and biuret **18**, and found that all are thermodynamically favourable (Figure 3, inserts). This suggests that the involvement of an appropriate catalyst such as BCl_3_ can make the formation of these products kinetically feasible. In the absence of the BCl_3_ catalyst, with or without stoichiometric water, the formation of **18** was not detected in any significant amounts. Although the activation barrier for the transformation **5**+**2**–>**7** is only 14.2 kcal/mol for the first turnover (Figure 2a), it increases for the subsequent turnovers. This is because product **18** is more strongly bound to the proton than the anion [BCl_3_(OH)]^−^ (Figure 2c). This causes the regeneration of the active catalyst [BCl_3_(OH_2_)] from **17** to be endergonic by about 7.9 kcal/mol (Figure 2c), raising the overall activation barrier to 14.2+7.9=22.1 kcal/mol for the subsequent turnovers. This suggests that the formation of product **18** could act as a type of inhibitor.

Finally, we turned our attention to the scope for the formation of the biuret/urea derivatives from corresponding aryl/alkyl isocyanates. BCl_3_ (20 mol %) and a 1 : 1 stoichiometric amount of aryl/alkyl isocyanate and water were reacted in 1,2‐C_2_H_4_Cl_2_ at 70 °C for 18–24 h to afford the corresponding biuret/urea derivatives in good yields (up to 76 %). Various aryl isocyanates bearing electron withdrawing/π‐releasing (F, Cl and Br), electron neutral (H), and electron donating (Me/OMe) at the *para*/*meta* positions of the aryl ring were employed for the decarbonylation reaction and corresponding biuret products (**18**–**24**) were obtained in good yields (Scheme 2, 40–73 %). However, aryl isocyanates bearing a strongly electron withdrawing groups (*para/meta*‐CF_3_), as well as cyclohexyl isocyanate, afforded the corresponding urea derivatives (**25**, **26** and **28**; yields 30–76 %) rather than the biuret products. This was confirmed by NMR spectroscopy and single crystal X‐ray diffraction.

**Scheme 2:**
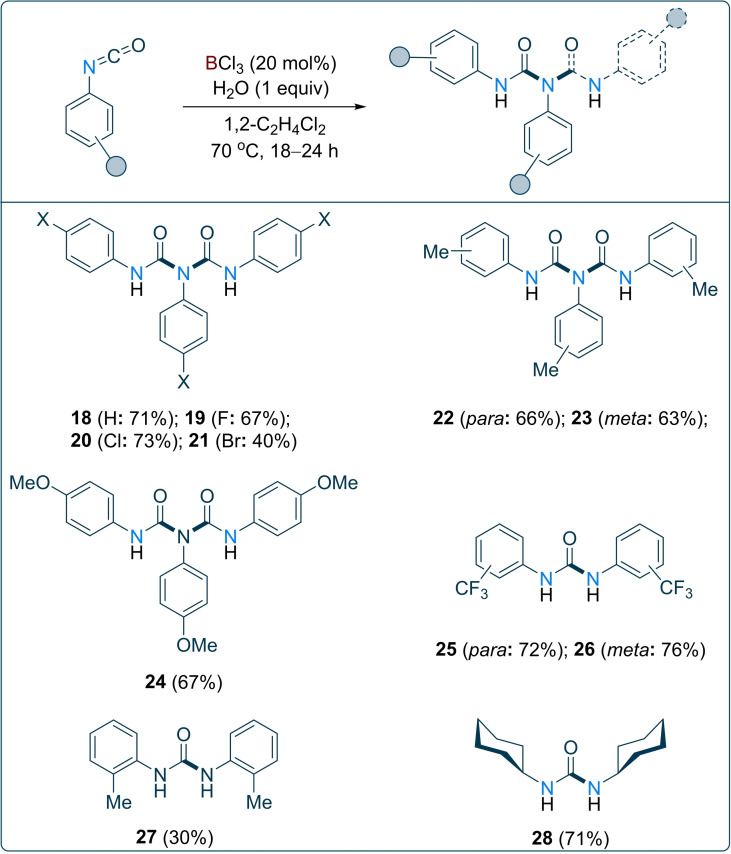
Decarbonylation of aryl/alkyl isocyanates using catalytic 20 mol % BCl_3_. Reactions were carried out in 0.1 mmol scale. Reported yields are isolated.

Prolonging the reaction time failed to afford the biuret products. The presence of a strong electron‐withdrawing group on the aryl ring reduces the nucleophilicity of the nitrogen atom of the urea intermediate, therefore, further reaction of **25**/**26** with another equivalent of isocyanate in cycle 3 fails to afford the biuret product. When *o*‐tolyl isocyanate was employed, urea **27** was formed as the major product in 30 % yield, and the corresponding biuret (**27 a**) was formed as a minor component in 10 % yield. Presumably, this is due to steric congestion caused by the *ortho* substitution on the aryl ring.

Attempted synthesis of unsymmetrical urea/biuret derivatives by employing two different functionalised aryl isocyanates unfortunately failed to afford the desired unsymmetrical urea compounds, instead complicated reaction mixtures were obtained.

An unexpected result was observed when 2‐methoxyphenyl isocyanate was used for the reaction. Indeed, 2‐methoxyphenyl isocyanate failed to afford the urea or biuret product. Vapour diffusion of the new product using CH_2_Cl_2_/pentane afforded a crystal suitable for X‐Ray diffraction which showed the formation of a benzoxazolone product **29** (Figure 4, left; Scheme 3, top). The stoichiometric reaction between 2‐methoxyphenyl isocyanate and BCl_3_ in dry CH_2_Cl_2_ at room temperature (23 °C) after 22 h afforded copious precipitate. Recrystallisation of the white precipitate from CH_2_Cl_2_ produced colourless crystals which revealed the formation of a benzoxazolone‐borane macro cycle **30 a** (61 % yield) composed of three units of 2(*3H*)‐benzoxazolone and three boron dichlorides (Figure 4, right). Hydrolysis of **30 a** leads to the clean formation of 2(*3H*)‐benzoxazolone **30** in 71 % yield. 2(*3H*)‐benzoxazolone scaffolds are medicinally relevant molecules having wide therapeutic applications as analgesic, anti‐inflammatory, anti‐psychotic and neuroprotective compounds.^[29]^ Therefore, a facile metal‐free synthesis to make such scaffold would be highly interesting.[^30^, ^31^]

**Figure 4:**
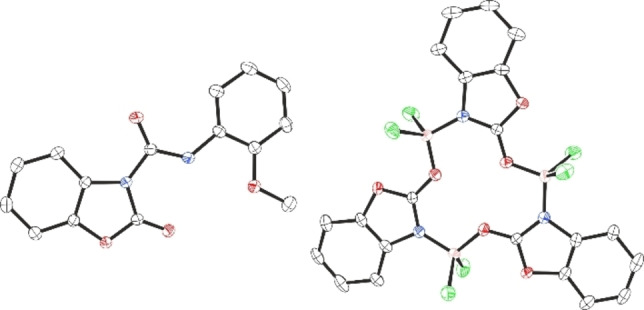
Crystal structure of **29** (left) and **30 a** (right). Thermal ellipsoids shown at 50 %. H atoms omitted for clarity. Carbon: black; Oxygen: red; Nitrogen: blue; Chlorine: green; Boron: pink.

**Scheme 3:**
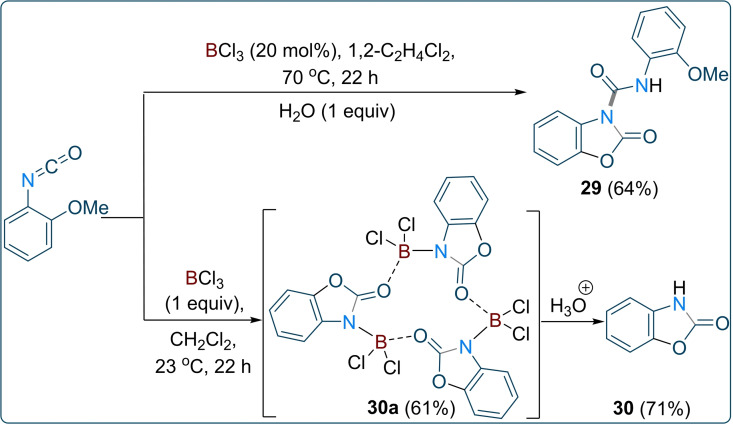
Intramolecular cyclisation of 2‐methoxyphenyl isocyanate using 20 mol % (top) and stoichiometric amounts of BCl_3_ (bottom).

In conclusion, a mild reaction protocol has been described towards decarbonylation of aryl/alkyl isocyanates employing catalytic amounts of BCl_3_ to form urea/biuret products. Detailed DFT studies were carried out to interpret the reaction mechanism which revealed that the active catalyst is the H_2_O→BCl_3_ adduct which is a Brønsted acid. Three competitive catalytic cycles have been proposed to account for the formation of the products.

Further investigation also revealed that 2(*3H*)‐benzoxazolone scaffolds can be synthesised in good yields when using 2‐methoxyphenyl isocyanate as starting material. Exploration of reactivities of other similar compounds including thiocyanates, ketenes, and allenes is currently in progress.

## Conflict of interest

The authors declare no conflict of interest.

## Supporting information

As a service to our authors and readers, this journal provides supporting information supplied by the authors. Such materials are peer reviewed and may be re‐organized for online delivery, but are not copy‐edited or typeset. Technical support issues arising from supporting information (other than missing files) should be addressed to the authors.

Supporting Information

## Data Availability

Deposition numbers 2125084 (**18**), 2128581 (**20**), 2160532 (**20a**), 2128580 (**22**), 2128579 (**25**), 2157033 (**29**), 2157032 (**30a**) contain the supplementary crystallographic data for this paper. These data are provided free of charge from the Cambridge Crystallographic Data Centre. Information about the data that underpins the results presented in this article can be found in the Cardiff University data catalogue at https://doi.org/10.17035/d.2022.0177867934.
