# Supplementary material for: Lewis Acid Assisted Brønsted Acid Catalysed Decarbonylation of Isocyanates: A Combined DFT and Experimental Study
Source: Chemistry. 2022 Jun 21;28(45):e202201422. doi: 10.1002/chem.202201422 (PMC9541586; doi:10.1002/chem.202201422)
Supplement: Supplementary file 1 — Supporting Information [file CHEM-28-0-s001.pdf]

Chemistry–A European Journal

Supporting Information

Lewis Acid Assisted Brønsted Acid Catalysed Decarbonylation of Isocyanates: A Combined DFT and Experimental Study

Ayan Dasgupta, Yara van Ingen, Michael G. Guerzoni, Kaveh Farshadfar, Jeremy M. Rawson, Emma Richards,* Alireza Ariafard,* and Rebecca L. Melen*

Table of Content

1	Experimental	3
	1.1 General experimental	3
2	Product Characterisation	4
	2.1 General procedure a	4
	2.2 Synthesis and spectral characterisation of products	4
3	NMR Spectra	13
4	Crystallographic Data	52
	4.1 Single crystal X-ray diffraction experimental	52
	4.2 Crystal structures	53
	4.3 X-ray refinement data	60
5	Computational Data	67
	5.1 Computational details	67
	5.2 Cartesian coordinates and total energies for the calculated structures	67
6	References	88

1. Experimental

1.1 General experimental:

Except for the starting materials, all reactions and manipulations were carried out under an atmosphere of dry, O_2-free nitrogen using standard double-manifold techniques with a rotary oil pump. A nitrogen-filled glove box (MBraun) was used to manipulate solids including the storage of starting materials, ambient temperature reactions, product recovery, and sample preparation for analysis. All solvents (toluene, dichloromethane, acetonitrile) were dried by employing a Grubbs-type column system (Innovative Technology) or a solvent purification system MB SPS-800 and stored under a nitrogen atmosphere. Anhydrous (with Sure/Seal) 1,2-dichloroethane (1,2-$C_2H_4Cl_2$) was purchased from Merck and dried over molecular sieves before use. Deuterated solvents were distilled and/or dried over molecular sieves before use. Chemicals were purchased from commercial suppliers and used as received. $B(C_6F_5)_3$ was prepared as per the standard literature report [1] and 1 M BCl_3 solution in hexane was purchased from Sigma-Aldrich and used as is. Aryl(alkyl) isocyanates were purchased from Sigma-Aldrich/Alfa Asear and used as is. Thin-layer chromatography (TLC) was performed on pre-coated aluminium sheets of Merck silica gel 60 F254 (0.20 mm). ^{1}H, ^{13}C, ^{11}B and ^{19}F NMR spectra were recorded on a Bruker Avance II 400 or Bruker Avance 500 spectrometer. All coupling constants are absolute values and are expressed in Hertz (Hz). ^{13}C NMR spectra were measured as ^{1}H decoupled. Yields are given as isolated yields. Chemical shifts are expressed as parts per million (ppm, δ) downfield of tetramethylsilane (TMS) and are referenced to $CDCl_3$ (7.26/77.16 ppm); CD_2Cl_2 (5.32/53.84 ppm); CD_3OD (3.31/49.00 ppm); CD_3CN (1.94/1.32 ppm) and $(CD_3)_2SO$ (2.50/39.52 ppm) as internal standard. NMR spectra were referenced to $CFCl_3$ (^{19}F).[2] The description of signals includes s = singlet, d = doublet, t = triplet, q = quartet, and m = multiplet, br. = broad. All spectra were analysed assuming a first order approximation. IR-Spectra were measured on a Shimadzu IRAffinity-1 photo-spectrometer. Mass spectra were measured on a Waters LCT Premier/XE or a Waters GCT Premier spectrometer. Ions were generated by the Atmospheric Solids, Analysis Probe (ASAP), Electrospray (ES) or Electron Ionisation (EI). The molecular ion peaks values quoted for either molecular ion (M^+), molecular ion plus or minus hydrogen ($M+H^+$, $M–H^–$), molecular ion plus sodium ($M+Na^+$).

2. Product Characterisation

2.1 General Procedure a:

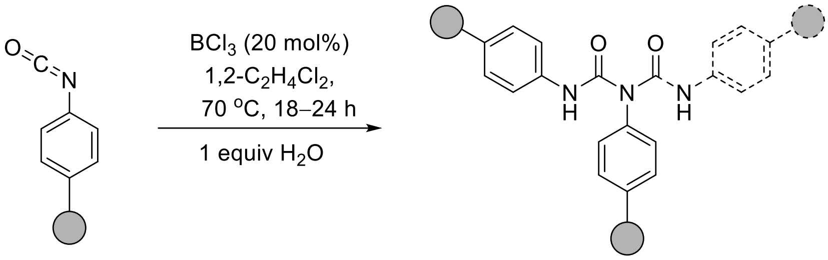

Boron trichloride [BCl_3; 1 M in hexane] (20 mol%) was diluted using 1,2-dichloroethane (1,2-$C_2H_4Cl_2$) (0.5 mL), added slowly dropwise to a premix of water (1 equiv) and 1,2-$C_2H_4Cl_2$ solution (1 mL) of the aryl(alkyl) isocyanate (1.3 equiv) with vigorous stirring. The reaction tube was sealed under a nitrogen atmosphere and heated at 70 °C for 18–24 h. All volatiles were removed *in vacuo* and the crude compound was purified *via* recrystallisation using acetonitrile-pentane mixture (5:1 v/v) unless otherwise stated.

2.2 Synthesis and spectral characterisation of products:

Synthesis of compound **18**.[3]

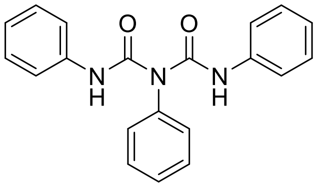

Synthesised in accordance with *General Procedure a* using BCl_3 solution (40 μL, 0.02 mmol), H_2O (3.6 μL, 0.20 mmol), phenyl isocyanate (24 mg, 0.20 mmol) in 1,2-$C_2H_4Cl_2$. Recrystallisation of the crude reaction mixture using an acetonitrile-pentane mixture (5:1 v/v) afforded compound **18** as a white solid. Yield: 16 mg, 0.05 mmol, 71%.

^{1}H NMR (500 MHz, $CDCl_3$, 298 K) δ: 8.93 (br. s, 2H, NH), 7.62–7.58 (m, 2H, Ar–CH), 7.57–7.53 (m, 1H, Ar–CH), 7.47–7.45 (m, 2H, Ar–CH), 7.41–7.39 (m, 4H, Ar–CH), 7.33–7.29 (m, 4H, Ar–CH), 7.13–7.10 (m, 2H, Ar–CH); ^{13}C NMR (126 MHz, $CDCl_3$, 298 K) δ: 153.6 (C=O), 137.3, 136.6, 130.7, 130.1, 129.9, 129.2, 124.6, 120.8; IR ν_{max} (cm^{-1}): 3055, 2987, 1716 (C=O), 1670, 1591, 1500, 1440, 1315, 1263, 1226, 1176, 1093, 1028; HRMS (ES+) $[M+H]^+$ $[C_{20}H_{18}N_3O_2]^+$: calculated 332.1399, found 332.1395.

Synthesis of compound **19**.[4]

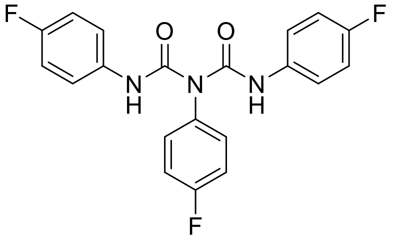

Synthesised in accordance with *General Procedure a* using BCl_3 solution (40 μL, 0.02 mmol), H_2O (3.6 μL, 0.20 mmol), 4-fluorophenyl isocyanate (28 mg, 0.20 mmol) in 1,2-$C_2H_4Cl_2$. Recrystallisation of the crude reaction mixture using an acetonitrile-pentane mixture (5:1 v/v) afforded compound **19** as a white solid. Yield: 17 mg, 0.04 mmol, 67%.

^{1}H NMR (500 MHz, CD_2Cl_2, 298 K) δ: 8.86 (br. s, 2H, NH), 7.45 (dd, J = 8.7, 4.9 Hz, 2H, Ar–CH), 7.38–7.35 (m, 4H, Ar–CH), 7.29 (t, J = 8.4 Hz, 2H, Ar–CH), 7.03 (t, J = 8.6 Hz, 4H, Ar–CH); ^{13}C NMR (126 MHz, CD_2Cl_2, 298 K) δ: 164.6 (d, $J_{C–F}$ = 250.0 Hz), 161.1 (d, $J_{C–F}$ = 243.1 Hz), 154.1 (C=O) , 133.9 (d, $J_{C–F}$ = 2.9 Hz), 133.0 (d, $J_{C–F}$ = 3.4 Hz), 132.5 (d, $J_{C–F}$ = 8.9 Hz), 123.3 (d, $J_{C–F}$ = 8.0 Hz), 118.1 (d, $J_{C–F}$ = 23.0 Hz), 116.2 (d, $J_{C–F}$ = 22.6 Hz); ^{19}F NMR (471 MHz, CD_2Cl_2, 298 K) δ: -111.34 (s, 1F, Ar–F), -118.64 (s, 2F, Ar–F); IR ν_{max} (cm^{-1}): 3031, 2984, 1721 (C=O), 1606, 1544, 1510, 1257, 1221, 1156, 1088; HRMS (ES+) $[M+H]^+$ $[C_{20}H_{15}F_3N_3O_2]^+$: calculated 386.1116, found 386.1124.

Synthesis of compound **20**.[5]

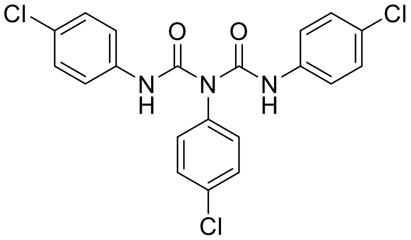

Synthesised in accordance with *General Procedure a* using BCl_3 solution (40 μL, 0.02 mmol), H_2O (3.6 μL, 0.20 mmol), 4-chlorophenyl isocyanate (31 mg, 0.20 mmol) in 1,2-$C_2H_4Cl_2$. Recrystallisation of the crude reaction mixture using an acetonitrile-pentane mixture (5:1 v/v) afforded compound **20** as a white solid. Yield: 21 mg, 0.05 mmol, 73%.

^{1}H NMR (500 MHz, $CDCl_3$, 298 K) δ: 8.86 (br. s, 2H, NH), 7.59–7.57 (m, 2H, Ar–CH), 7.39–7.38 (m, 2H, Ar–CH), 7.35–7.33 (m, 4H, Ar–CH), 7.30–7.27 (m, 4H, Ar–CH); ^{13}C NMR (126 MHz, $CDCl_3$, 298 K) δ: 153.1 (C=O), 136.5, 135.7, 134.7, 131.3, 131.1, 130.0, 129.3, 122.0; IR ν_{max} (cm^{-1}): 3055, 2987, 1718 (C=O), 1591, 1517, 1440, 1315, 1263, 1226, 1178, 1093, 1072; HRMS (ES+) $[M+H]^+$ $[C_{20}H_{15}Cl_3N_3O_2]^+$: calculated 434.0230, found 434.0231.

Synthesis of compound **20a**.

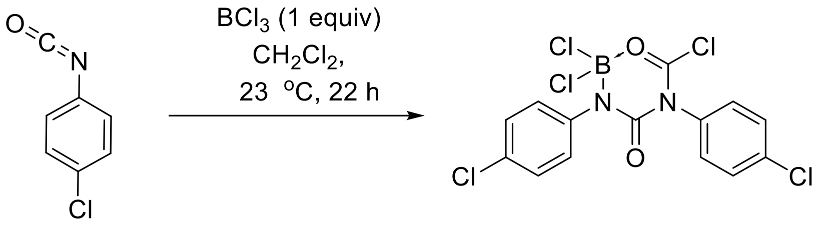

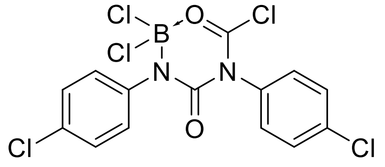

The above reaction was performed in a glovebox under a nitrogen atmosphere. Boron trichloride [BCl_3; 1 M in CH_2Cl_2] (1 equiv, 0.4 mmol, 0.4 mL) was added to a stirred solution of 4-chloro phenylisocyanate (1 equiv, 0.4 mmol, 61.4 mg) in dichloromethane (CH_2Cl_2, 2 mL). The sample vial was capped and stirred for 22 h at room temperature (23 °C). All volatiles were removed *in vacuo* giving a yellow solid. The product was purified by recrystallisation from slow evaporation of the redissolved solid in minimal $CDCl_3$. The product **20a** was obtained as a colourless crystalline solid. Yield: 47 mg, 0.19 mmol, 56%. Single crystals suitable for X-Ray diffraction were grown from $CDCl_3$ at -30 °C.

^{1}H NMR (500 MHz, $CDCl_3$, 298 K) δ: 7.57–7.54 (dt, *J* = 8.75, 3.06 Hz, 2H, Ar–CH), 7.40–7.35 (m, 4H, Ar–CH), 7.33–7.31 (dt, *J* = 8.75, 3.06 Hz, 2H, Ar–CH); ^{13}C NMR (126 MHz, $CDCl_3$, 298 K) δ: 165.8 (C=O), 146.3 (C=O), 138.1, 136.1, 134.3, 133.0, 130.7, 129.6, 129.5, 129.0; ^{11}B NMR (160 MHz, $CDCl_3$, 298 K) δ: 7.82.

Synthesis of compound **21**.

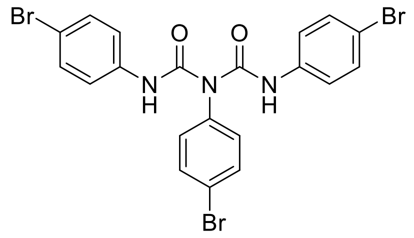

Synthesised in accordance with *General Procedure a* using BCl_3 solution (40 μL, 0.02 mmol), H_2O (3.6 μL, 0.20 mmol), 4-bromo phenyl isocyanate (40 mg, 0.20 mmol) in 1,2-$C_2H_4Cl_2$. All the solvents were removed under vacuum. The crude compound was purified by washing the crude white solid with diethyl ether (3 × 5 mL) to afford **21** as a pure compound. Yield: 15 mg, 0.05 mmol, 40%.

^{1}H NMR (500 MHz, DMSO-d_6, 298 K) δ: 9.44 (br. s, 2H, NH), 7.61 (d, *J* = 8.2 Hz, 3H, Ar–CH), 7.43 (s, 6H, Ar–CH), 7.23 (d, *J* = 8.3 Hz, 3H, Ar–CH); ^{13}C NMR (126 MHz, DMSO-d_6) δ 152.4 (C=O), 139.1, 133.9, 132.5, 131.5, 124.1, 119.9, 118.6, 113.2; IR ν_{max} (cm^{-1}): 3055, 2987, 1718 (C=O), 1591, 1517, 1440, 1315, 1263, 1226, 1178, 1093, 1072; HRMS (ES+) $[M+H]^+$ $[C_{20}H_{15}Br_3N_3O_2]^+$: calculated 565.8714, found 565.8718.

Synthesis of compound **22**.[6]

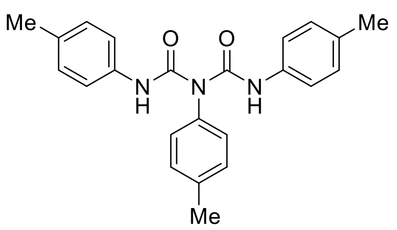

Synthesised in accordance with *General Procedure a* using BCl_3 solution (40 µL, 0.02 mmol), H_2O (3.6 µL, 0.20 mmol), 4-tolyl isocyanate (27 mg, 0.20 mmol) in 1,2-$C_2H_4Cl_2$. Recrystallisation of the crude reaction mixture using hot acetonitrile afforded compound **22** as a white solid. Yield: 16 mg, 0.05 mmol, 66%.

^{1}H NMR (500 MHz, $CDCl_3$, 298 K) δ: 8.90 (br. s, 2H, NH), 7.38–7.36 (m, 2H, Ar–CH), 7.32–7.30 (m, 2H, Ar–CH), 7.28–7.26 (m, 4H, Ar–CH), 7.12–7.08 (m, 4H, Ar–CH), 2.44 (s, 3H, Me), 2.30 (s, 6H, Me); ^{13}C NMR (126 MHz, $CDCl_3$, 298 K) δ: 153.8 (C=O), 140.1, 134.8, 134.2, 134.0, 131.3, 129.9, 129.63, 129.59, 120.8, 21.5, 21.0; IR ν_{max} (cm^{-1}): 3005, 2989, 1716 (C=O), 1595, 1543, 1508, 1490, 1400, 1274, 1261, 1228, 1166, 1089; HRMS (ES+) $[M+H]^+$ $[C_{23}H_{24}N_3O_2]^+$: calculated 374.1869, found 374.1864.

Synthesis of compound **23**.

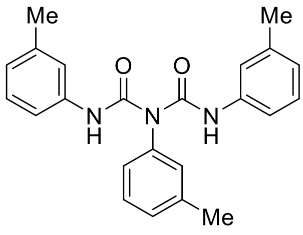

Synthesised in accordance with *General Procedure a* using BCl_3 solution (40 µL, 0.02 mmol), H_2O (3.6 µL, 0.20 mmol), 3-tolyl isocyanate (27 mg, 0.20 mmol) in 1,2-$C_2H_4Cl_2$. Recrystallisation of the crude reaction mixture using hot acetonitrile afforded compound **22** as a white solid. Yield: 15 mg, 0.04 mmol, 63%.

^{1}H NMR (500 MHz, $CDCl_3$, 298 K) δ: 8.91 (br. s, 2H, N–H), 7.47 (t, J = 7.8 Hz, 1H, Ar–CH), 7.35–7.33 (m, 1H, Ar–CH), 7.25–7.23 (m, 4H, Ar–CH), 7.21–7.17 (m, 4H, Ar–CH), 6.94–6.92 (m, 2H, Ar–CH), 2.45 (s, 3H, Me), 2.32 (s, 6H, Me); ^{13}C NMR (126 MHz, $CDCl_3$, 298 K) δ: 153.7 (C=O), 140.9, 139.1, 137.3, 136.6, 130.8, 130.5, 130.4, 129.0, 126.7, 125.4, 121.4, 117.8, 21.6 (Me), 21.5 (Me); IR ν_{max} (cm^{-1}): 3414, 3053, 2922, 1716 (C=O), 1670, 1606, 1569, 1489, 1265, 1195, 1149, 1089; HRMS (ES+) $[M+H]^+$ $[C_{23}H_{24}N_3O_2]^+$: calculated 374.1869, found 374.1868.

Synthesis of compound **24**.

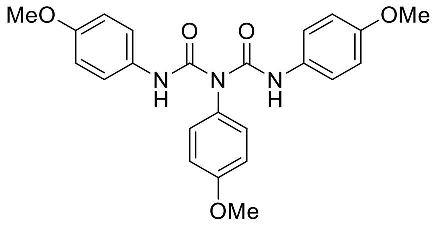

Synthesised in accordance with *General Procedure a* using BCl_3 solution (40 µL, 0.02 mmol), H_2O (3.6 µL, 0.20 mmol), 4-methoxyphenyl isocyanate (30 mg, 0.20 mmol) in 1,2-$C_2H_4Cl_2$. Recrystallisation of the crude reaction mixture using acetonitrile-pentane mixture (7:3 v/v) afforded compound **24** as an off-white solid. Yield: 19 mg, 0.05 mmol, 67%.

^{1}H NMR (500 MHz, $CDCl_3$, 298 K) δ: 8.85 (br. s, 2H, NH), 7.36–7.34 (m, 2H, Ar–CH), 7.30 (d, *J* = 9.0 Hz, 4H, Ar–CH), 7.08–7.06 (m, 2H, Ar–CH), 6.85–6.83 (m, 4H, Ar–CH), 3.87 (s, 3H, OMe), 3.78 (s, 6H, OMe); ^{13}C NMR (126 MHz, $CDCl_3$, 298 K) δ: 160.4 (C=O), 156.7, 154.2, 131.0, 130.4, 129.0, 122.7, 115.8, 114.3, 55.8 (OMe), 55.6 (OMe); IR ν_{max} (cm^{-1}): 3055, 2987, 1718 (C=O), 1591, 1517, 1440, 1315, 1263, 1226, 1178, 1093, 1072; HRMS (ES+) $[M+H]^+$ $[C_{23}H_{24}N_3O_5]^+$: calculated 422.1709, found 422.1716.

Synthesis of compound **25**.[7]

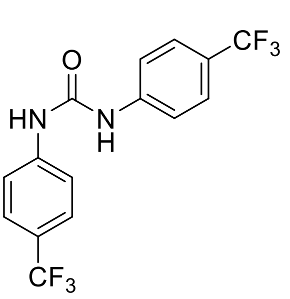

Synthesised in accordance with *General Procedure a* using BCl_3 solution (40 µL, 0.02 mmol), H_2O (3.6 µL, 0.20 mmol), 4-(trifluoromethyl)phenyl isocyanate (37 mg, 0.20 mmol) in 1,2-$C_2H_4Cl_2$. Recrystallisation of the crude reaction mixture using hot acetonitrile afforded compound **25** as a pale yellow solid. Yield: 17 mg, 0.05 mmol, 72%.

^{1}H NMR (500 MHz, CD_3CN, 298 K) δ: 7.76 (br. s, 2H, N–H), 7.64 (d, *J* = 9.2 Hz, 4H, Ar–CH), 7.59 (d, *J* = 8.7 Hz, 4H, Ar–CH); ^{13}C NMR (126 MHz, CD_3CN, 298 K) δ: 153.1 (C=O), 143.8, 127.0 (q, *J* = 3.8 Hz, CF_3), 119.6, 118.3; ^{19}F NMR (471 MHz, CD_3CN, 298 K) δ: -62.34 (s, 6F, Ar–CF_3); IR ν_{max} (cm^{-1}): 3005, 2989, 1715 (C=O), 1639, 1608, 1535, 1521, 1448, 1408, 1386, 1326, 1274, 1261, 1182, 1161, 1109, 1064; HRMS (ES+) $[M+H]^+$ $[C_{15}H_{11}F_6N_2O]^+$: calculated 349.0776, found 349.0779.

Synthesis of compound **26**.[8]

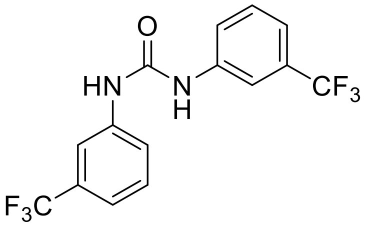

Synthesised in accordance with *General Procedure a* using BCl_3 solution (40 µL, 0.02 mmol), H_2O (3.6 µL, 0.20 mmol), 3-(trifluoromethyl)phenyl isocyanate (37 mg, 0.20 mmol) in 1,2-$C_2H_4Cl_2$. recrystallisation of the crude reaction mixture using hot acetonitrile afforded compound **26** as an off white solid. Yield: 18 mg, 0.05 mmol, 76%.

^{1}H NMR (400 MHz, CD_3OD, 298 K) δ: 7.80 (s, 2H, Ar–CH), 7.53 (d, *J* = 7.7 Hz, 2H, Ar–CH), 7.38 (t, *J* = 8.0 Hz, 2H, Ar–CH), 7.21 (d, *J* = 7.8 Hz, 2H, Ar–CH); ^{13}C NMR (101 MHz, CD_3OD, 298 K) δ: 155.0 (C=O) 141.4, 132.4, 132.1, 130.7, 127.0, 124.3, 123.4, 120.1 (q, *J* = 3.9, Ar–CF_3), 116.5 (q, *J* = 3.9 Hz, Ar–CF_3); ^{19}F NMR (376 MHz, CD_3OD, 298 K) δ: -64.28 (s, 6F, Ar–CF_3); IR ν_{max} (cm^{-1}): 3005, 2929, 1710 (C=O), 1656, 1571, 1492, 1467, 1448, 1425, 1328, 1311, 1274, 1259, 1217, 1156, 1120, 1095, 1068; HRMS (ES+) $[M+H]^+$ $[C_{15}H_{11}F_6N_2O]^+$: calculated 349.0776, found 349.0769. We failed to detect any N-H proton in the ^{1}H NMR spectra.

Synthesis of compound **27**.[9]

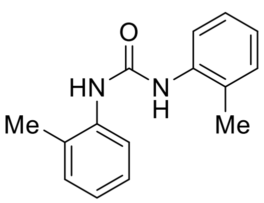

Synthesised in accordance with *General Procedure a* using BCl_3 solution (40 μL, 0.02 mmol), H_2O (3.6 μL, 0.20 mmol), 2-tolyl isocyanate (27 mg, 0.20 mmol) in 1,2-$C_2H_4Cl_2$. All the solvents were removed under vacuum. The crude compound was purified by washing the crude white solid with diethyl ether (3 × 5 mL) to afforded **27** as a pure compound. Yield: 7 mg, 0.03 mmol, 30%.

^{1}H NMR (500 MHz, DMSO-d_6, 298 K) δ: 8.25 (br. s, 2H, N–H), 7.79 (d, *J* = 9.3 Hz, 2H), 7.17 (d, *J* = 7.5 Hz, 2H), 7.13 (t, *J* = 7.7 Hz, 2H), 6.94 (t, *J* = 7.4 Hz, 2H), 2.26 (s, 6H, Me); ^{13}C NMR (126 MHz, DMSO-d_6, 298 K) δ: 153.0 (C=O), 137.5, 130.2, 127.8, 126.1, 122.7, 121.5, 18.1 (Me); IR ν_{max} (cm^{-1}): 3031, 2984, 1721 (C=O), 1606, 1544, 1510, 1257, 1221, 1156, 1088; HRMS (ES+) $[M+H]^+$ $[C_{15}H_{17}N_2O]^+$: calculated 241.1341, found 241.1350.

Synthesis of compound **27a**.

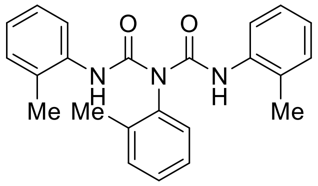

Synthesised in accordance with *General Procedure a* using BCl_3 solution (40 μL, 0.02 mmol), H_2O (3.6 μL, 0.20 mmol), 2-tolyl isocyanate (27 mg, 0.20 mmol) in 1,2-$C_2H_4Cl_2$. All the solvents were removed and a white solid was obtained. The white solid was washed several times with diethyl ether and the desired compound crystallised out from the ether when using pentane as a anti solvent afforded pure compound **27a**. Yield: 3 mg, 0.01 mmol, 10%.

^{1}H NMR (500 MHz, CD_2Cl_2, 298 K) δ: 8.85 (br. s, 2H, N–H), 7.80 (d, *J* = 8.0 Hz, 2H, Ar–CH), 7.47–7.42 (m, 4H, Ar–CH), 7.19–7.17 (m, 4H, Ar–CH), 7.05 (td, *J* = 7.4, 1.4 Hz, 2H, Ar–CH), 2.36 (d, *J* = 1.3 Hz, 3H, Me), 2.14 (s, 6H, Me); ^{13}C NMR (126 MHz, CD_2Cl_2, 298 K) δ: 153.6 (C=O), 138.3, 136.3, 132.3, 130.7, 130.4, 130.1, 129.9, 128.4, 127.0, 125.2, 122.8, 17.9 (Me), 17.6 (Me); IR ν_{max} (cm^{-1}): 3031, 2984, 1721 (C=O), 1606, 1544, 1510, 1257, 1221, 1156, 1088; HRMS (ES+) $[M+H]^+$ $[C_{23}H_{24}N_3O_2]^+$: calculated 349.1869, found 349.1868.

Synthesis of compound **28**.[10]

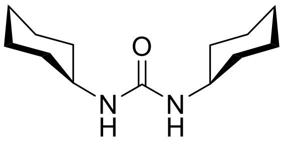

Synthesised in accordance with *General Procedure a* using BCl_3 solution (40 μL, 0.02 mmol), H_2O (3.6 μL, 0.20 mmol), cyclohexyl isocyanate (25 mg, 0.20 mmol) in 1,2-$C_2H_4Cl_2$. recrystallisation of the crude reaction mixture using hot acetonitrile-methanol (9:1) afforded compound **28** as a colourless crystalline solid. Yield: 11 mg, 0.05 mmol, 71%.

^{1}H NMR (500 MHz, CD_3OD, 298 K) δ: 3.43–3.39 (m, 4H, cyclohexyl C–H), 3.30 (dt, *J* = 13.1, 3.8 Hz, 4H, cyclohexyl C–H), 3.16 (dt, *J* = 12.8, 3.9 Hz, 2H, cyclohexyl C–H), 2.96–2.87 (m, 4H,

cyclohexyl C–H), 2.79–2.66 (m, 1H, cyclohexyl C–H); ^{13}C NMR (126 MHz, CD_3OD, 298 K) δ: 159.9 (C=O), 49.7, 34.8, 26.8, 26.1; IR ν_{max} (cm^{-1}): 3005, 2989, 1641, 1608, 1537, 1521, 1446, 1409, 1386, 1274, 1261, 1184, 1161, 1109, 1064, 1016; HRMS (ES+) $[M+H]^+$ $[C_{13}H_{25}N_2O]^+$: calculated 225.1967, found 225.1972.

We failed to detect any N-H proton in ^{1}H NMR spectra.

Synthesis of compound **29**.

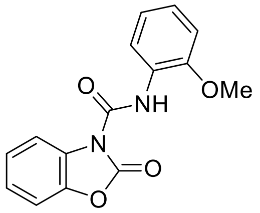

Synthesised in accordance with *General Procedure a* using BCl_3 solution (40 µL, 0.02 mmol), H_2O (3.6 µL, 0.20 mmol), 2-tolyl isocyanate (30 mg, 0.20 mmol) in 1,2-$C_2H_4Cl_2$. The crude reaction mixture was purified *via* preparative thin layer chromatography using hexane/ethyl acetate (75:25 v/v) as eluent. The desired compound **29** was obtained as a white solid. Yield: 18 mg, 0.06 mmol, 64%.

^{1}H NMR (500 MHz, $CDCl_3$, 298 K) δ: 10.50 (br. s, 1H, N–H), 8.22 (dd, *J* = 8.0, 1.6 Hz, 1H, Ar–CH), 8.10–8.08 (m, 1H, Ar–CH), 7.25–7.18 (m, 3H, Ar–CH), 7.07–7.04 (m, 1H, Ar–CH), 6.97–6.93 (m, 1H, Ar–CH), 6.88 (dd, *J* = 8.2, 1.3 Hz, 1H, Ar–CH), 3.89 (s, 3H, OMe); ^{13}C NMR (126 MHz, $CDCl_3$, 298 K) δ: 153.1 (C=O), 149.1, 147.1, 142.0, 128.1, 126.5, 125.2, 124.9, 124.8, 121.2, 120.1, 115.9, 110.5, 110.2, 56.1 (OMe); IR ν_{max} (cm^{-1}): 3250, 1772, (C=O), 1724 (C=O), 1602, 1490, 1469, 1354, 1321, 1253, 1220, 1178, 1147, 1120, 1072, 1022; HRMS (ES+) $[M+Na]^+$ $[C_{15}H_{12}N_2O_4Na]^+$: calculated 307.0695, found 307.0696.

Synthesis of compound **30**.

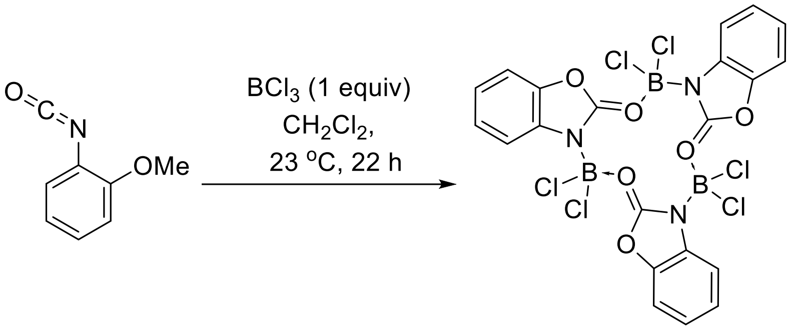

30

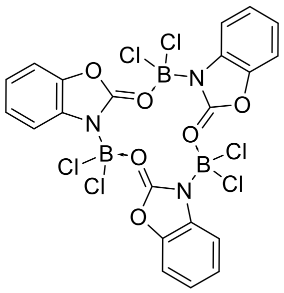

The above reaction was performed in a glovebox under a nitrogen atmosphere. Boron trichloride [BCl_3; 1 M in CH_2Cl_2] (1 equiv, 0.4 mmol, 0.4 mL) was added to a stirred solution of 2-methoxy phenylisocyanate (1 equiv, 0.4 mmol, 53 μL) in CH_2Cl_2 (2 mL). A white precipitate formed upon the addition of BCl_3. The sample vial was capped and stirred for 18 h at room temperature (23 °C). The mother liquor was removed, the precipitate washed with pentane (3 × 1 mL) and all volatiles were removed *in vacuo*. The product was obtained as a white solid. Yield: 53 mg, 0.08 mmol, 61%. Single crystals suitable for X-Ray diffraction were grown from slow evaporation of redissolved precipitate under gentle heating in minimal CH_2Cl_2, at -30 °C.

^{1}H NMR (500 MHz, $CDCl_3$, 298 K) δ: 8.15 (d, *J* = 8.1, 1H, Ar-CH), 7.73 (d, *J* = 8.0, 1H, Ar-CH), 7.55 (m, 2H, Ar-CH); ^{13}C NMR (126 MHz, $CDCl_3$, 298 K) δ: 156.1 (C=O), 145.2, 130.4, 126.8, 126.5, 119.0, 111.6; ^{11}B NMR (160 MHz, $CDCl_3$, 298 K) δ: 5.22.

Synthesis of compound **30a**.[11]

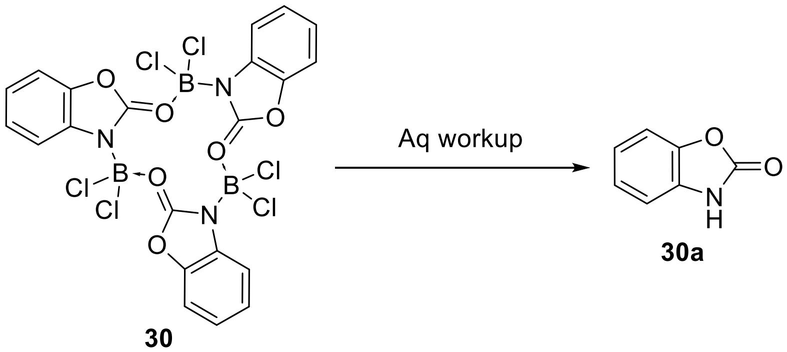

30a

30

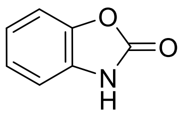

A saturated aqueous solution of ammonium chloride (20 mL) was added to a suspension of **30** (1 equiv, 0.16 mmol, 107.9 mg) in acetonitrile (20 mL) and stirred at 40 °C for 1 h. The compound dissolved after 20 minutes of heating. The mixture was allowed to cool to room temperature, and the organic compounds were extracted with

CH_2Cl_2 (3 × 10 mL). The combined organic fractions were was washed with brine (1 × 20 mL), dried over $NaSO_4$, and the volatiles were removed *in vacuo*. The product was purified *via* recrystallisation from CH_2Cl_2. The desired product was obtained as a colourless crystalline solid. Yield: 48 mg, 0.36 mmol, 71%.

^{1}H NMR (500 MHz, $CDCl_3$, 298 K) δ: 10.00 (br. s, 1H, N–H), 7.22–7.10 (m, 4H, Ar–CH); ^{13}C NMR (126 MHz, $CDCl_3$, 298 K) δ: 156.6 (C=O), 144.0, 129.6, 124.4, 122.8, 110.4, 110.2; IR ν_{max} (cm^{-1}): 3201 (N–H), 2920, 1882, 1730 (C=O), 1624, 1560, 1477, 1398, 1305, 1251, 1145; HRMS (ES–) $[M-H]^-$ $[C_7H_4NO_2]^-$: calculated 134.0242, found 134.0239.

Control reaction: formation of aniline:

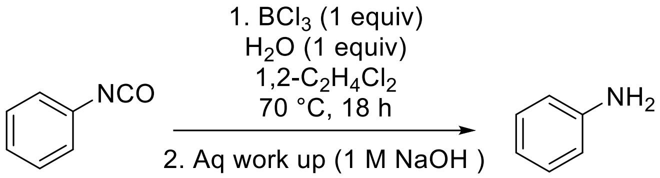

Water (1 equiv., 1.00 mmol, 18 μL) was added into to a stirred solution of phenyl isocyanate (1 equiv., 1.00 mmol, 109 μL) in 1,2-$C_2H_4Cl_2$ (2 mL) in a Schlenk tube and was stirred for 15 minutes at room temperature, under a nitrogen atmosphere. Boron trichloride [BCl_3; 1 M in heptane] (1 equiv. 1.00 mmol, 1.00 mL) was added into the reaction mixture and an immediate formation of a precipitate was observed. The Schlenk tube was equipped with a condenser and heated at 70 ^{o}C for 18 h. The following work up was completed under ambient conditions. The volatiles were removed *in vacuo* and the resultant precipitate was suspended in acetonitrile (5 mL), treated with an NaOH solution (1 M in water, 30 mL) and stirred for 1 h. The organic compounds were extracted with 1,2-$C_2H_4Cl_2$ (3 × 5 mL), the combined organic fractions were washed with brine solution (1 × 20 mL), dried over $NaSO_4$, and concentrated. All volatiles were removed *in vacuo* and an NMR spectrum was obtained from the crude reaction mixture in $CDCl_3$ (Figure S39) which clearly confirmed the formation of aniline.

3. NMR Spectra

Figure S1: ^{1}**H NMR** (500 MHz, $CDCl_3$, 298 K) spectrum of **18**.

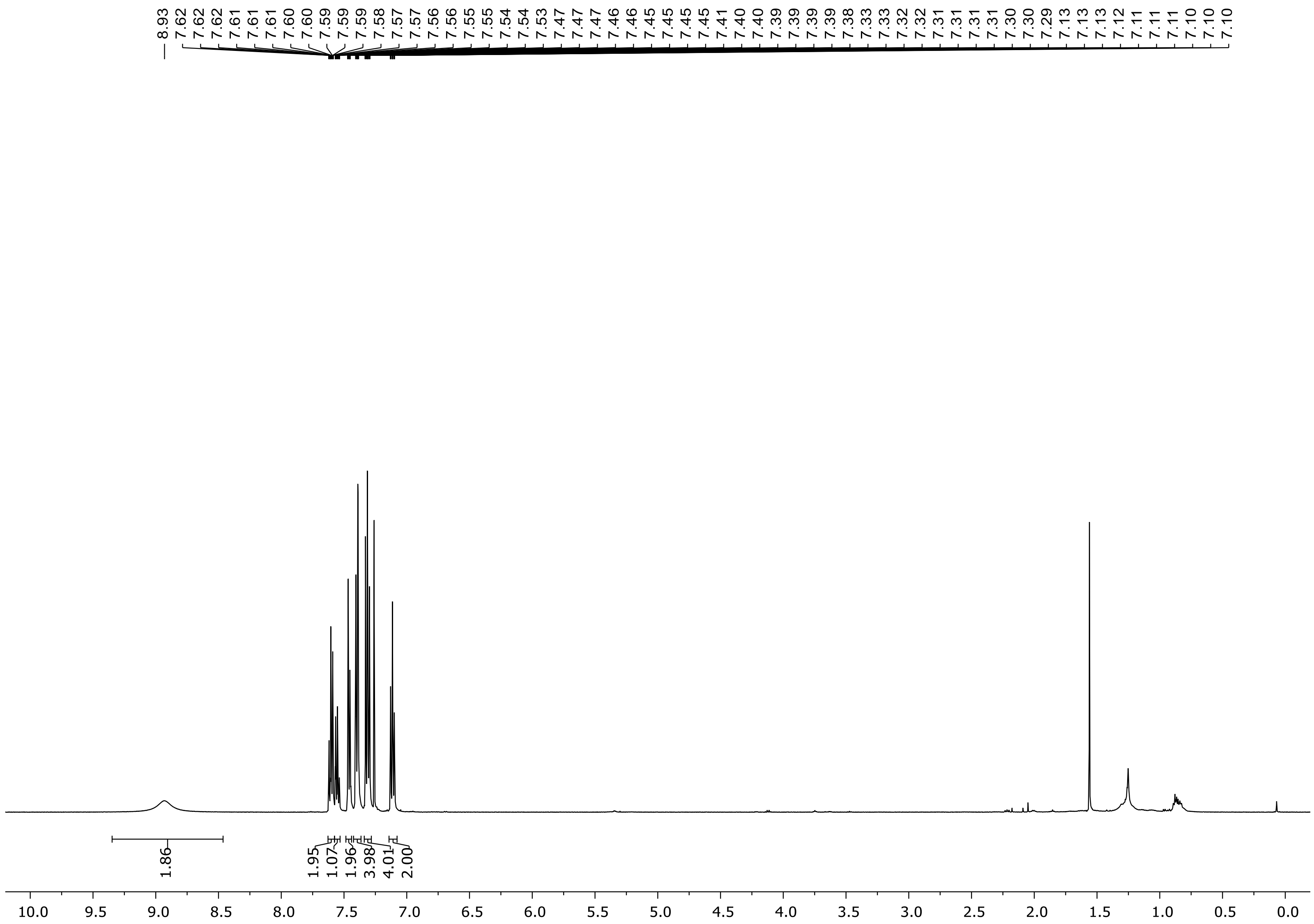

Figure S2: **^{13}C NMR** (126 MHz, $CDCl_3$, 298 K) spectrum of **18**.

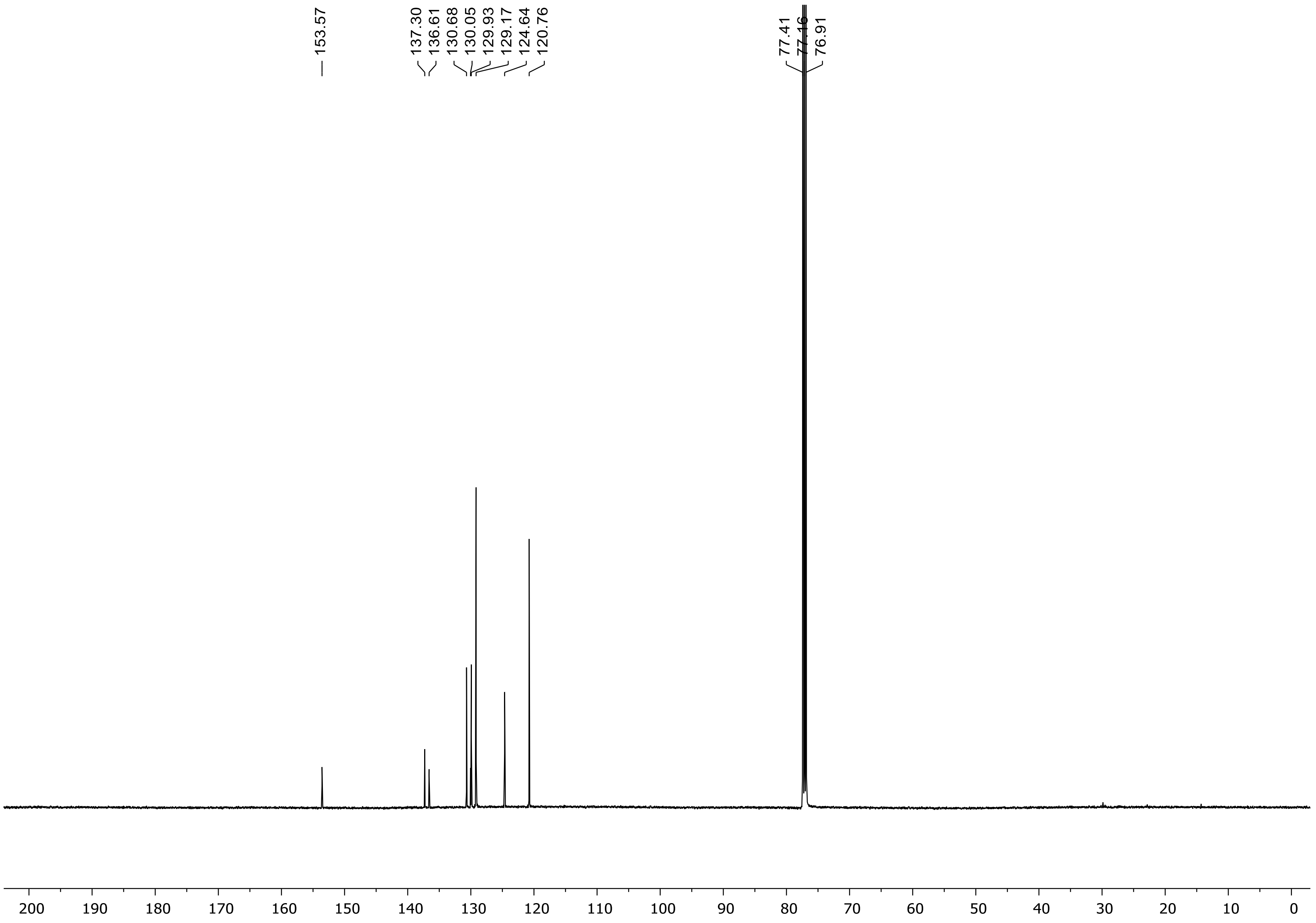

Figure S3: ^{1}**H NMR** (500 MHz, CD_2Cl_2, 298 K) spectrum of **19**.

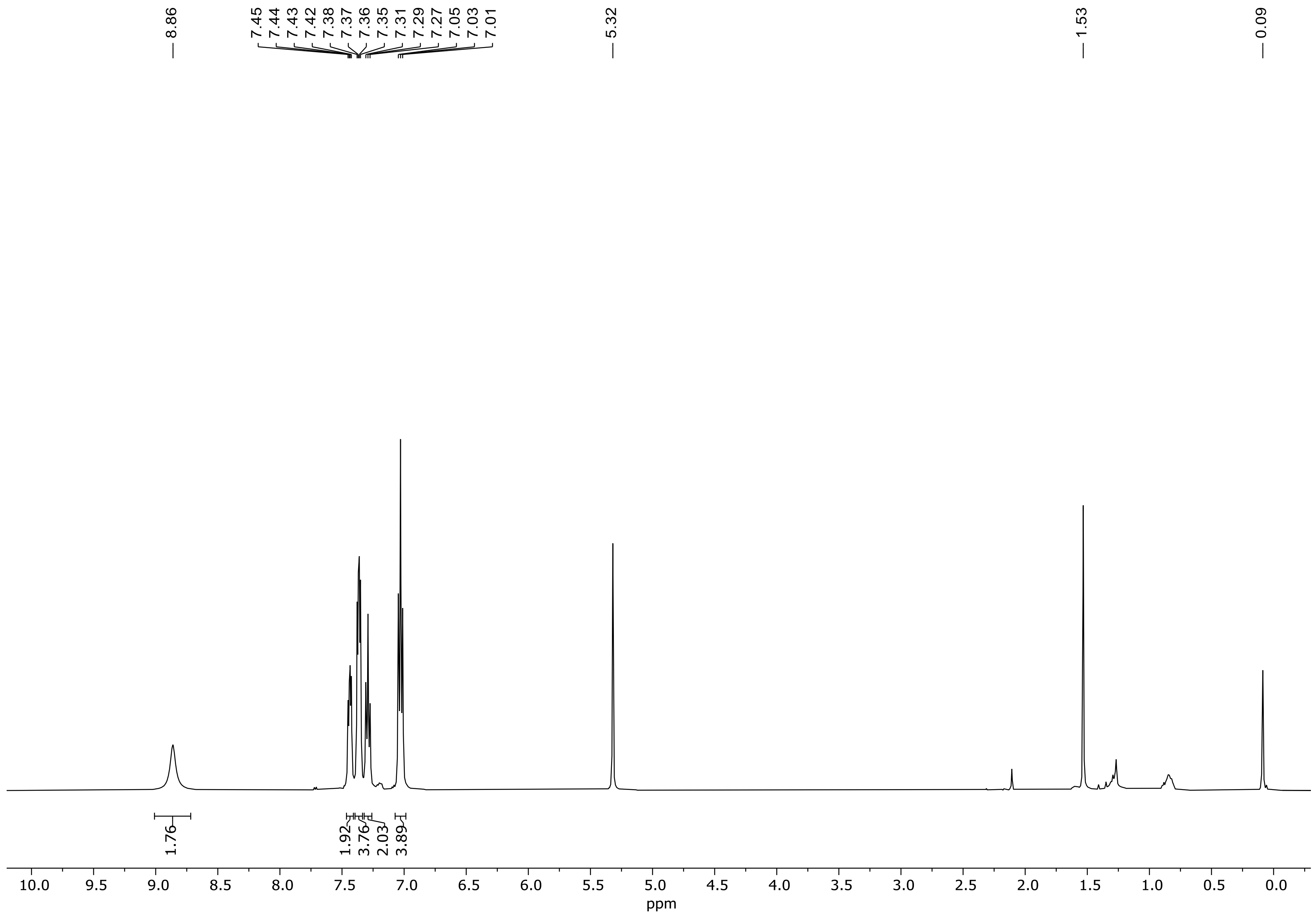

Figure S4: **^{13}C NMR** (126 MHz, CD_2Cl_2, 298 K) spectrum of **19**.

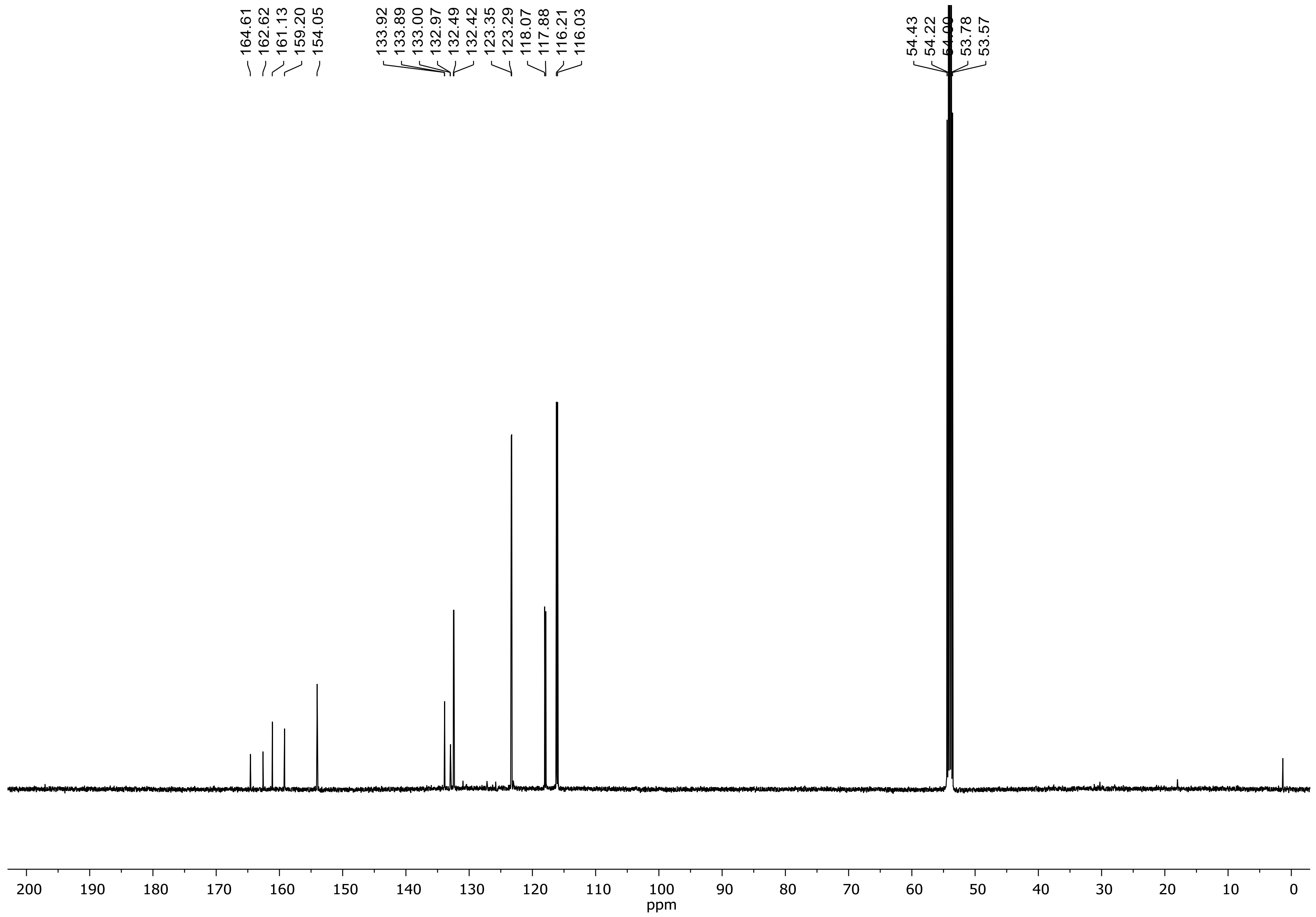

Figure S5: ^{19}**F NMR** (471 MHz, $CDCl_3$, 298 K) spectrum of **19**.

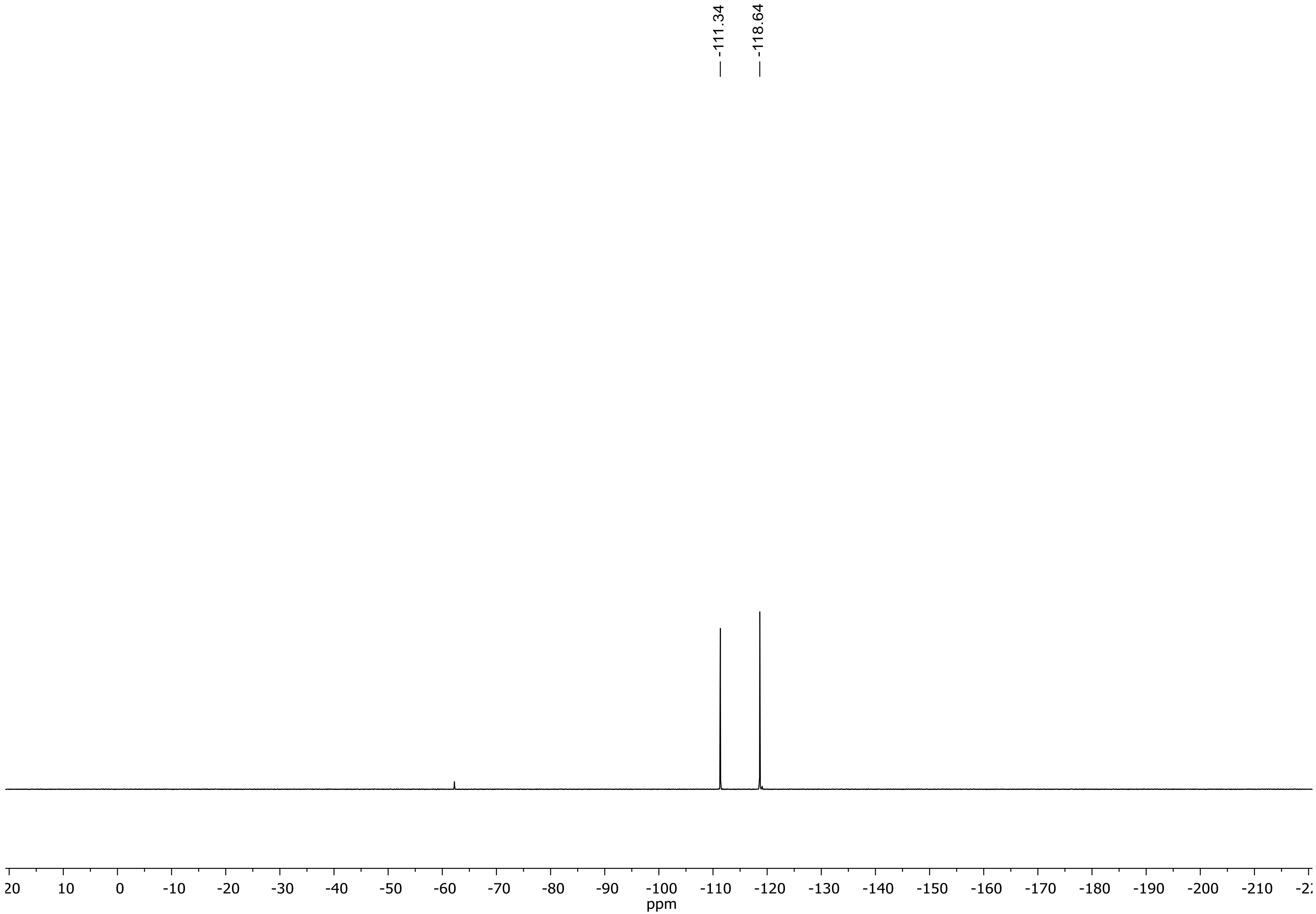

Figure S6: **^{1}H NMR** (500 MHz, $CDCl_3$, 298 K) spectrum of **20**.

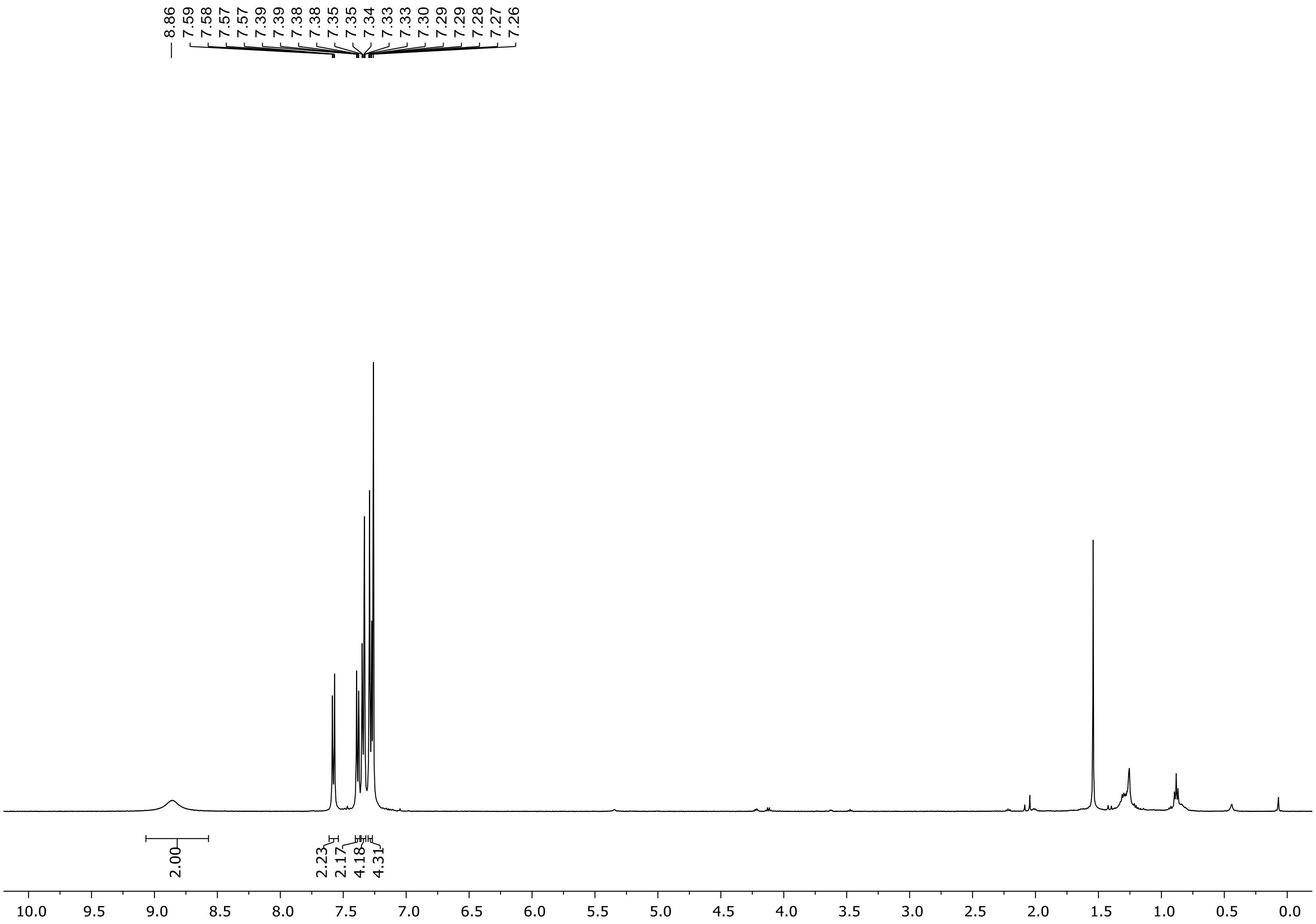

Figure S7: **^{13}C NMR** (126 MHz, $CDCl_3$, 298 K) spectrum of **20**.

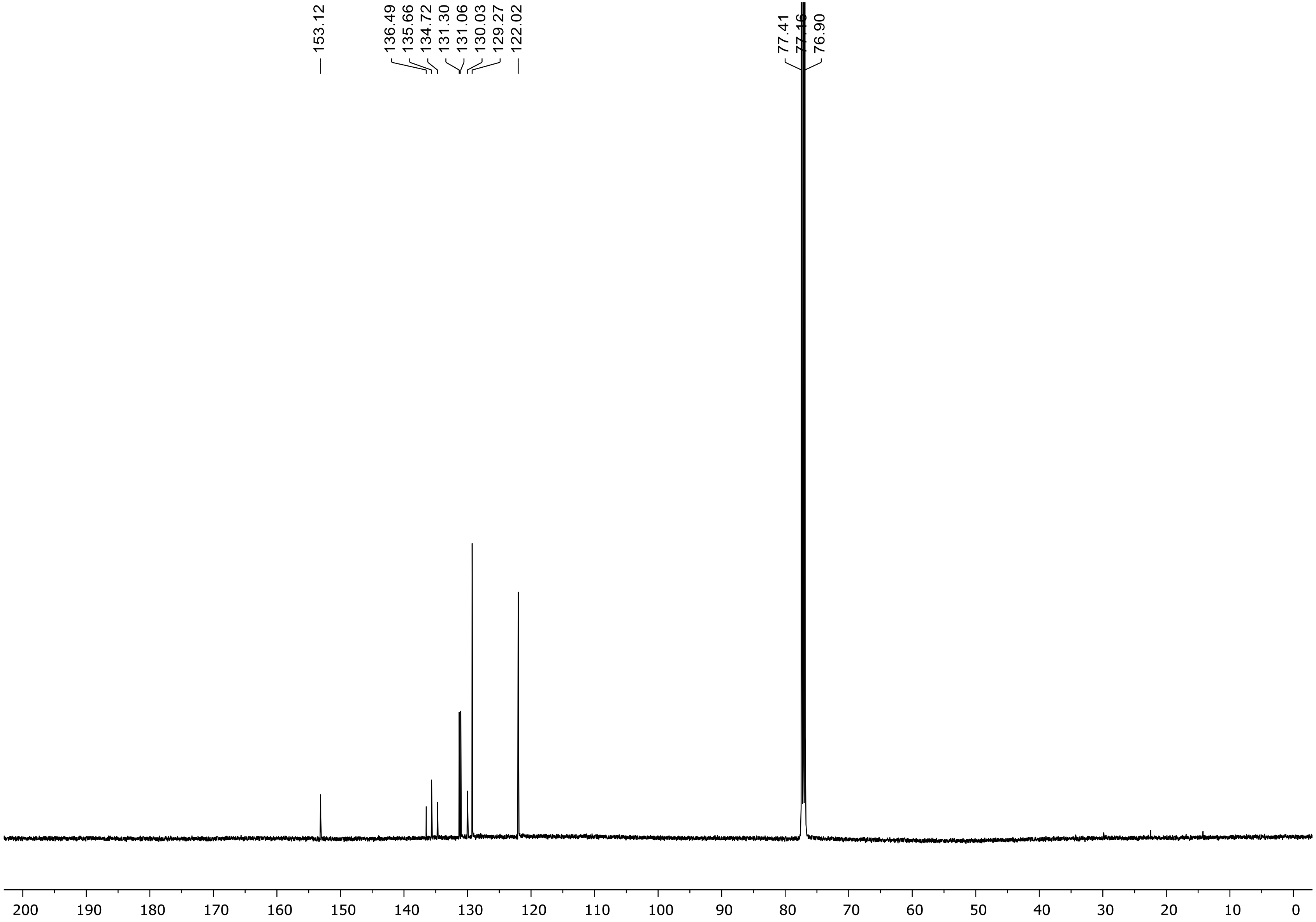

Figure S8: **^{1}H NMR** (500 MHz, $CDCl_3$, 298 K) spectrum of **20a**.

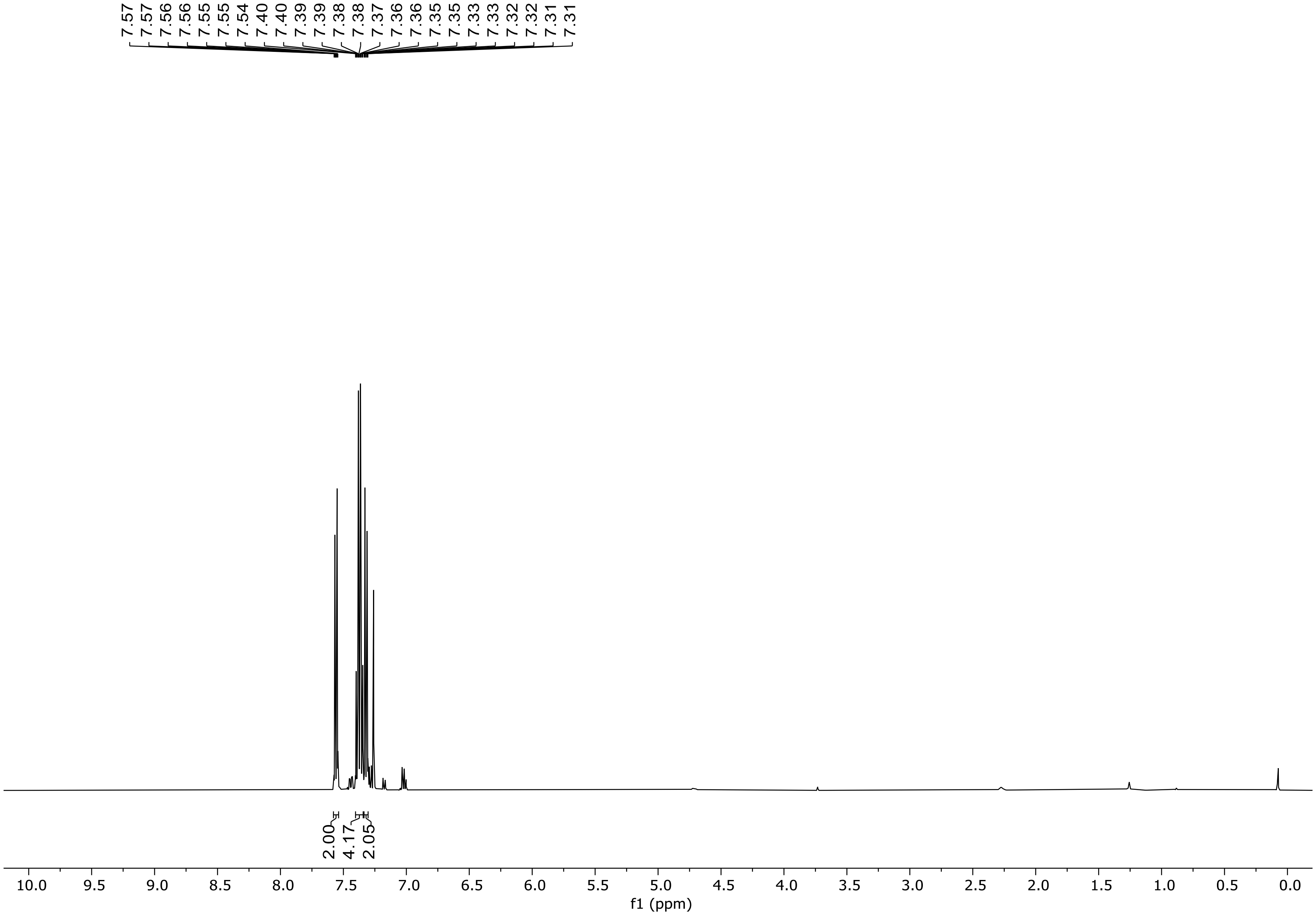

Figure S9: ^{13}**C NMR** (126 MHz, $CDCl_3$, 298 K) spectrum of **20a**.

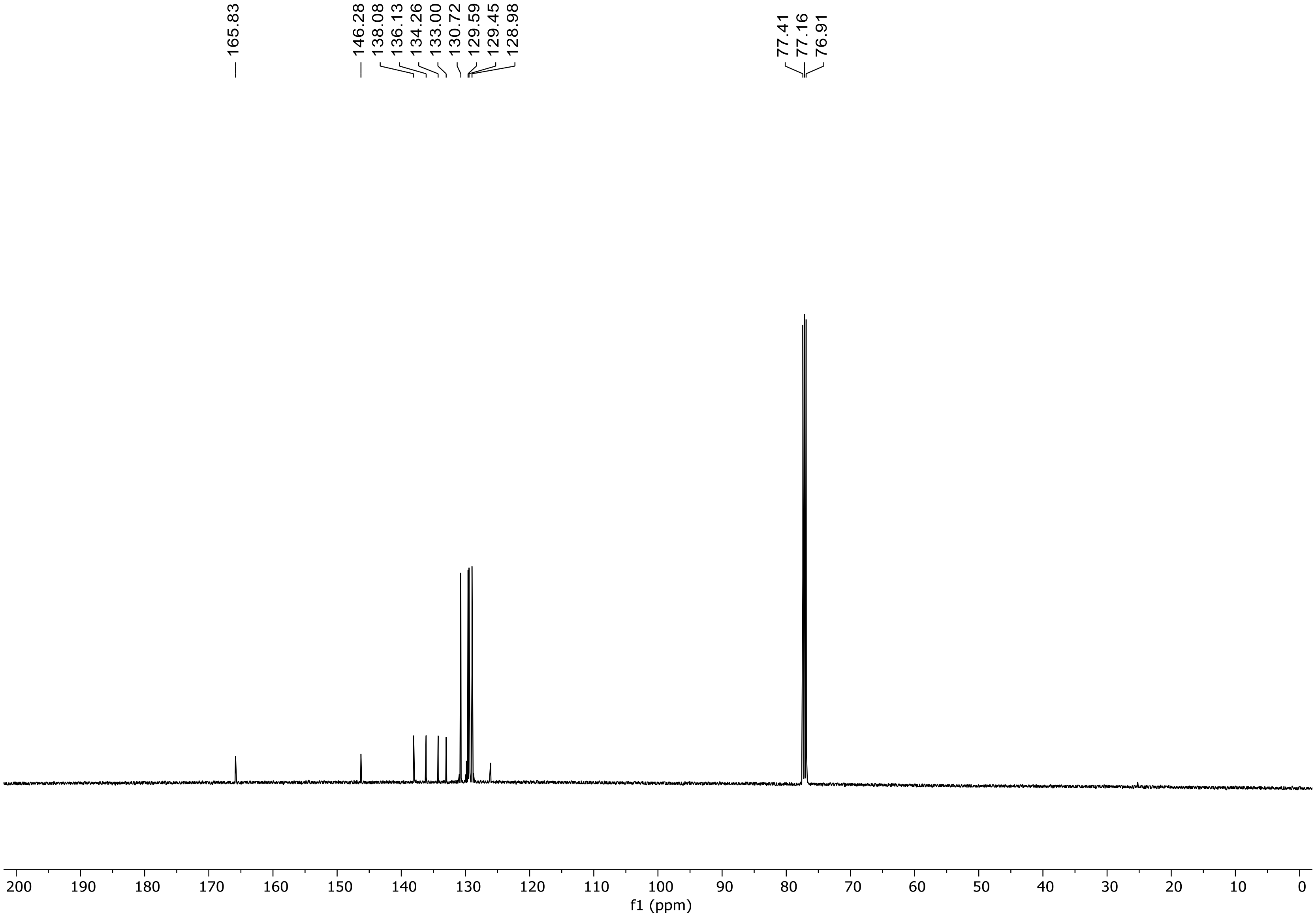

Figure S10: **^{11}B NMR** (160 MHz, $CDCl_3$, 298 K) spectrum of **20a**.

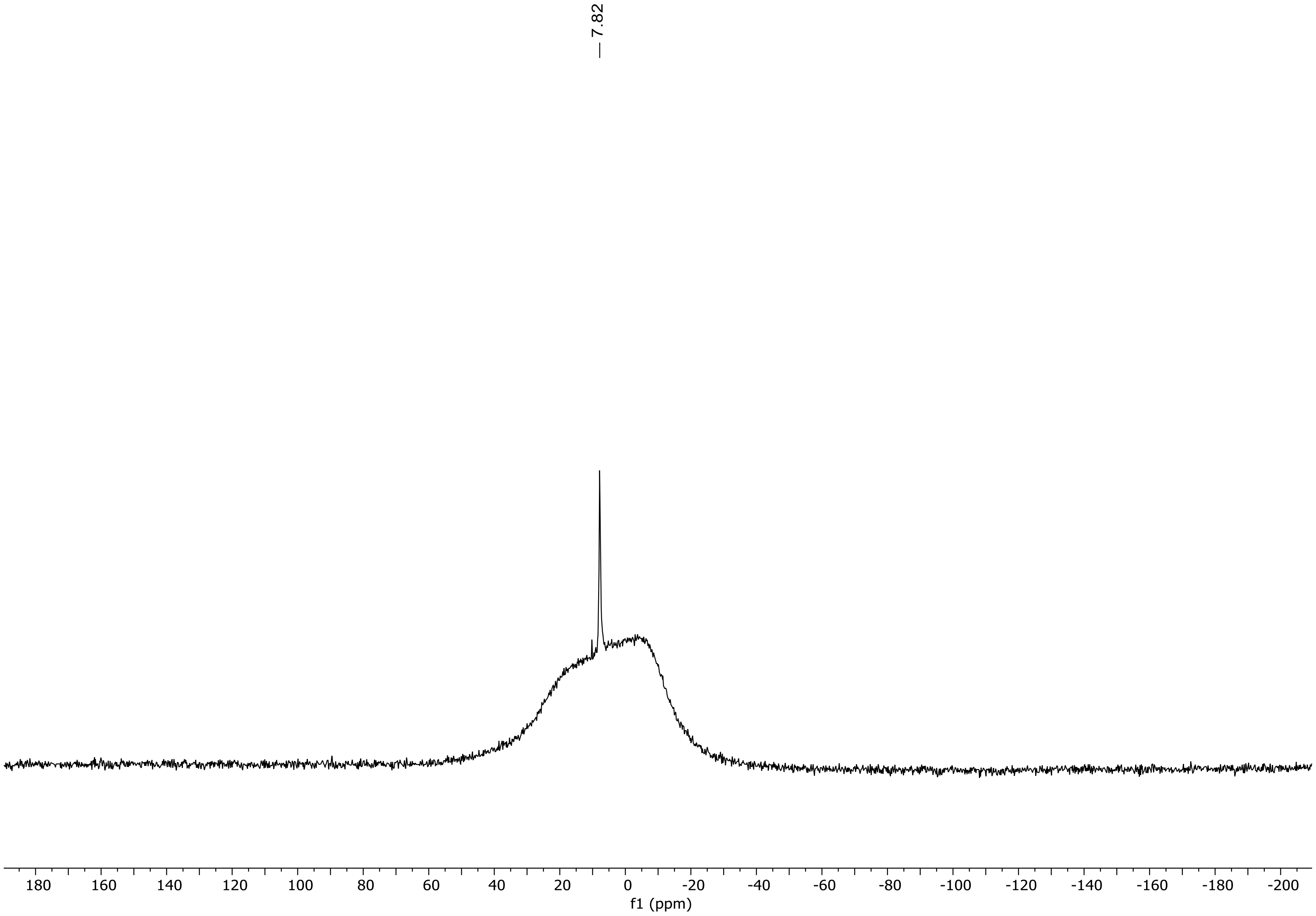

Figure S11: **^{1}H NMR** (500 MHz, DMSO-d_6, 298 K) spectrum of **21**.

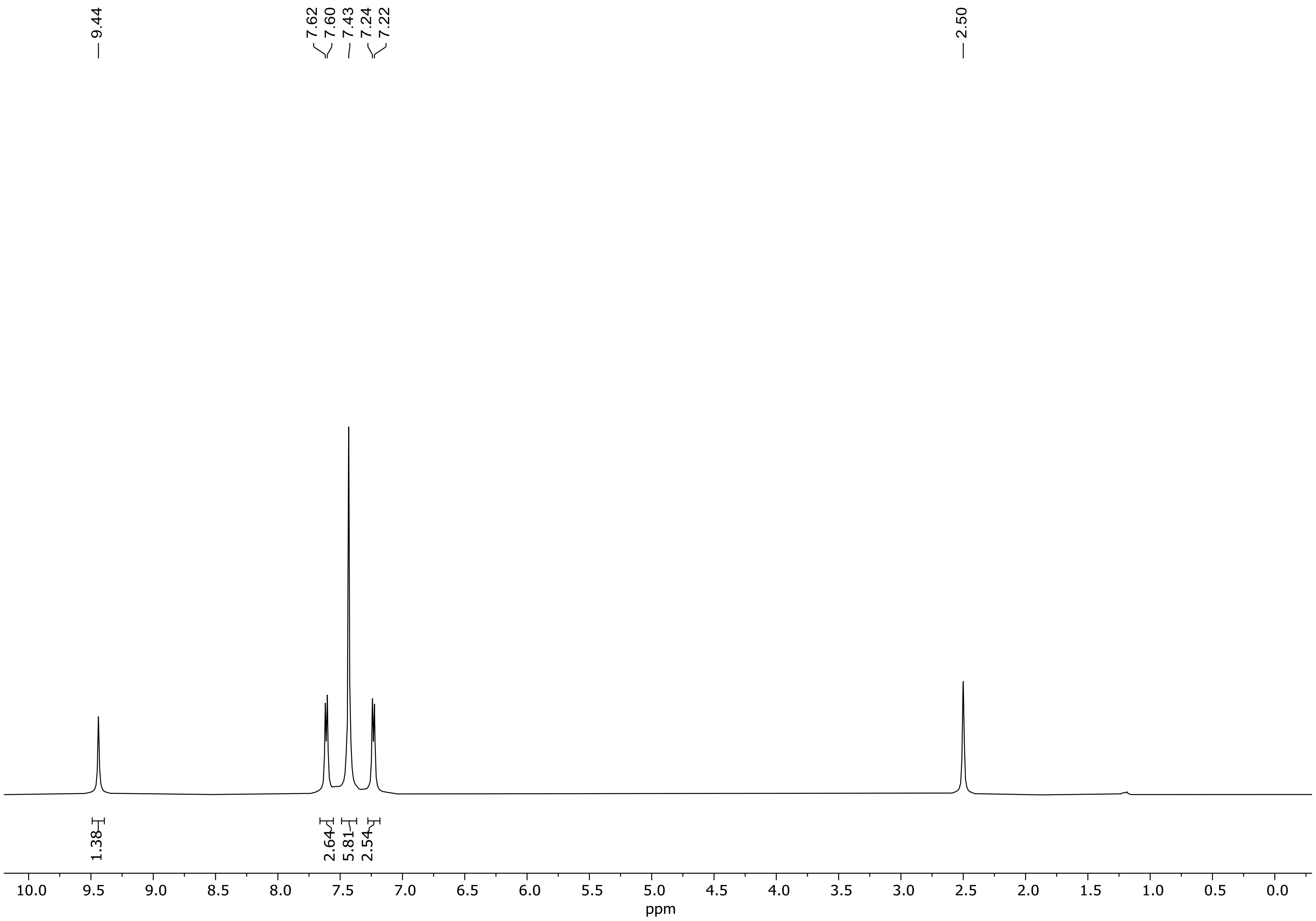

Figure S12: ^{13}**C NMR** (126 MHz, DMSO-d_6, 298 K) spectrum of **21**.

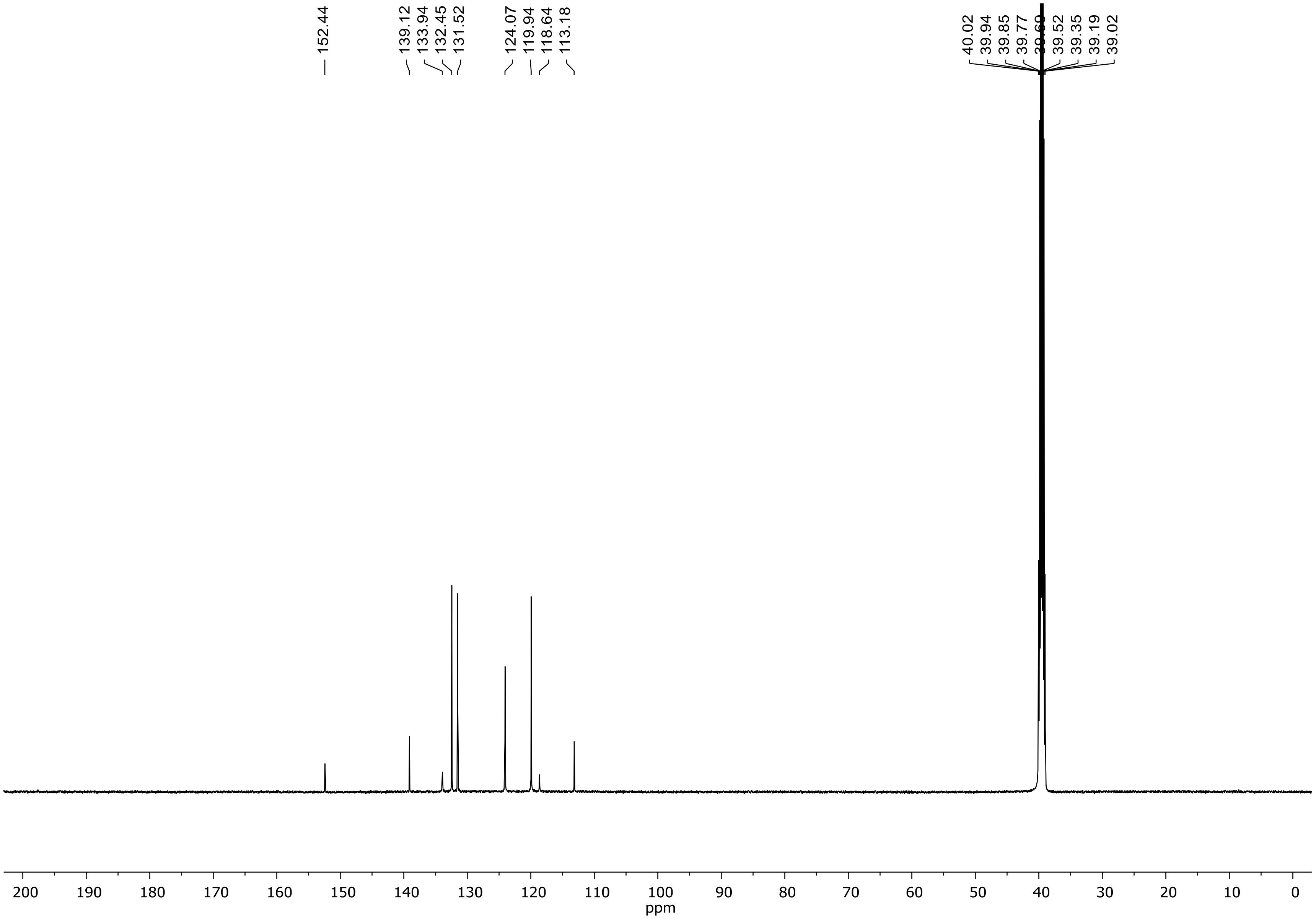

Figure S13: **^{1}H NMR** (500 MHz, $CDCl_3$, 298 K) spectrum of **22**.

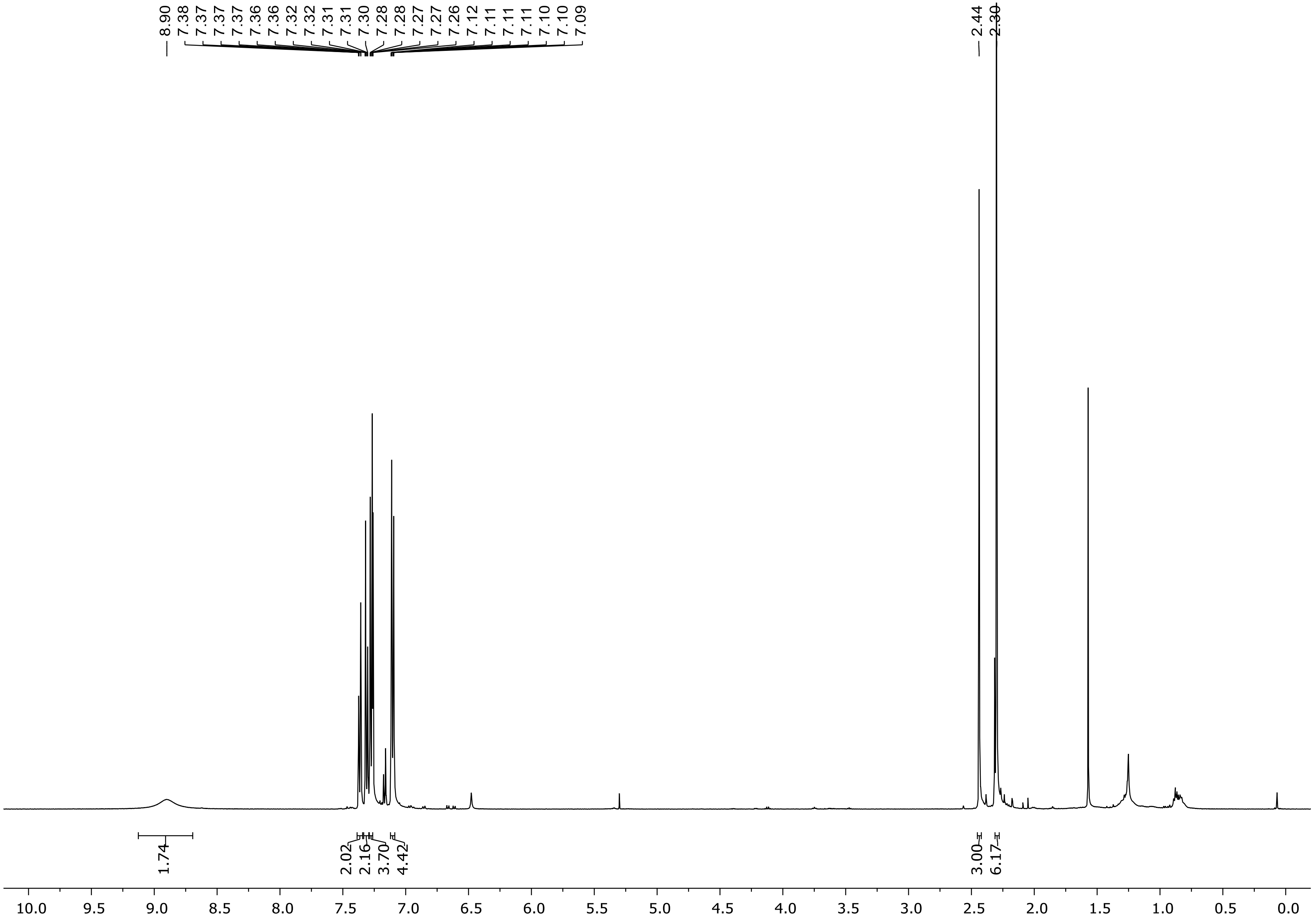

Figure S14: **^{13}C NMR** (126 MHz, $CDCl_3$, 298 K) spectrum of **22**.

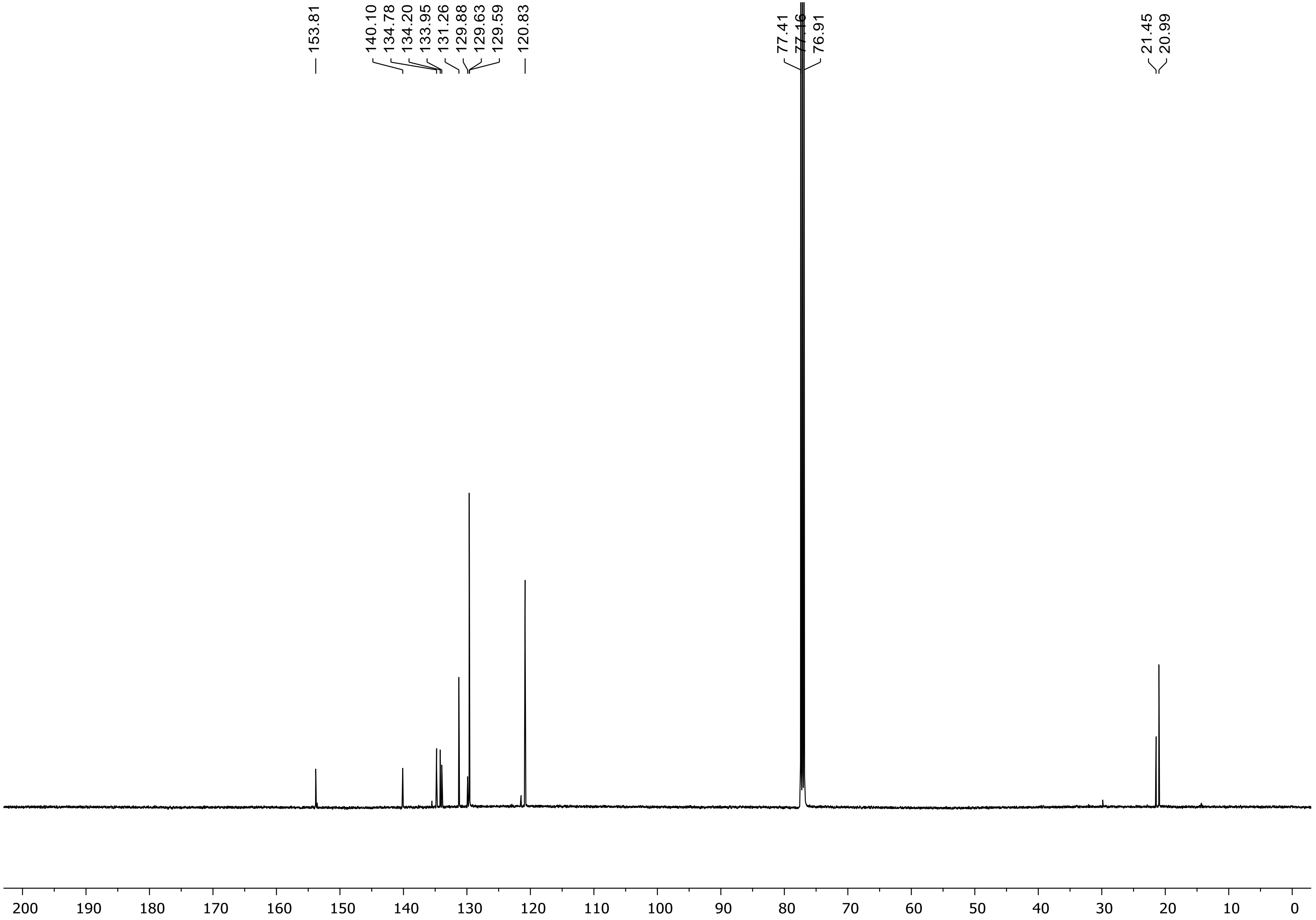

Figure S15: **^{1}H NMR** (500 MHz, $CDCl_3$, 298 K) spectrum of **23**.

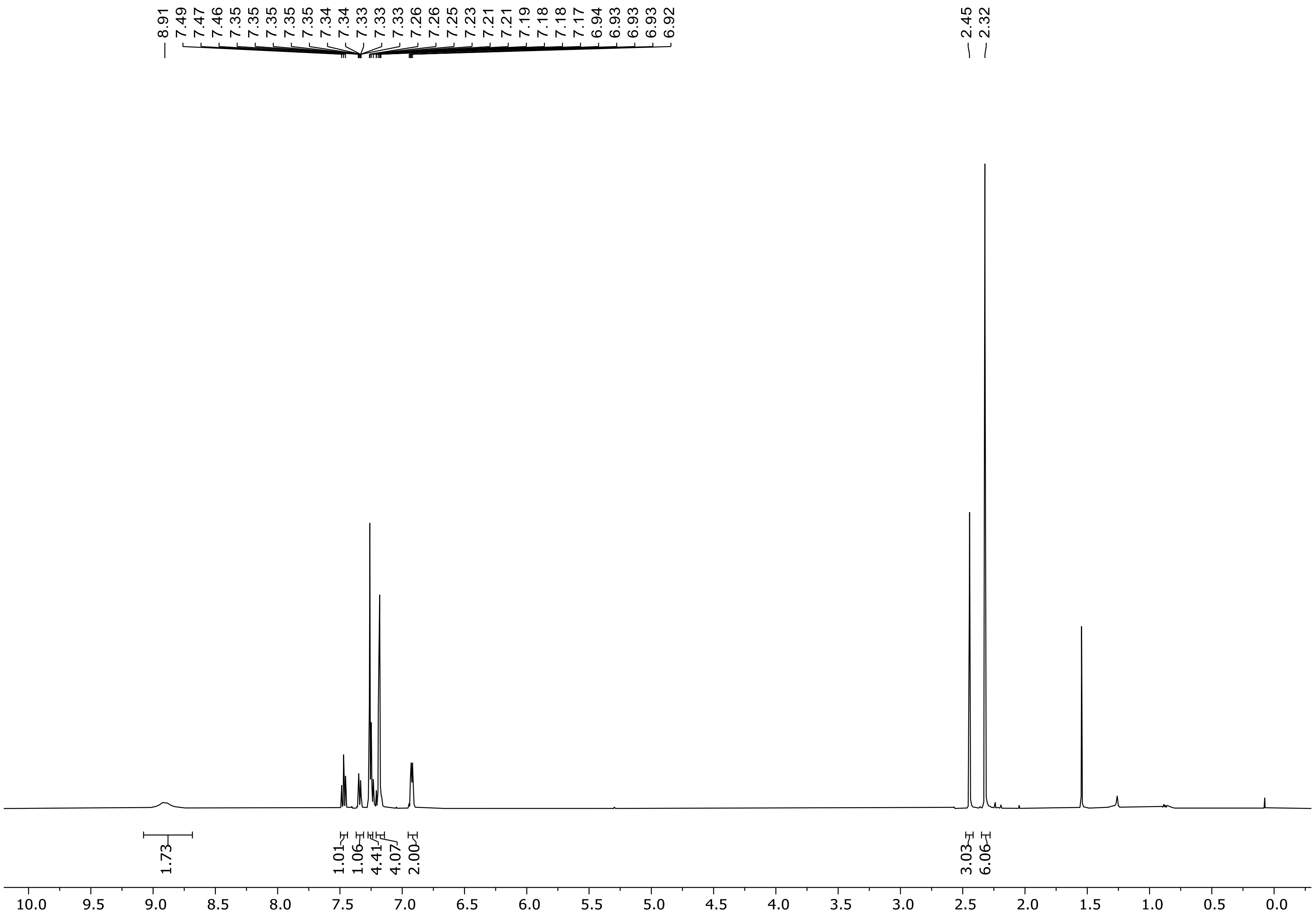

Figure S16: **^{13}C NMR** (126 MHz, $CDCl_3$, 298 K) spectrum of **23**.

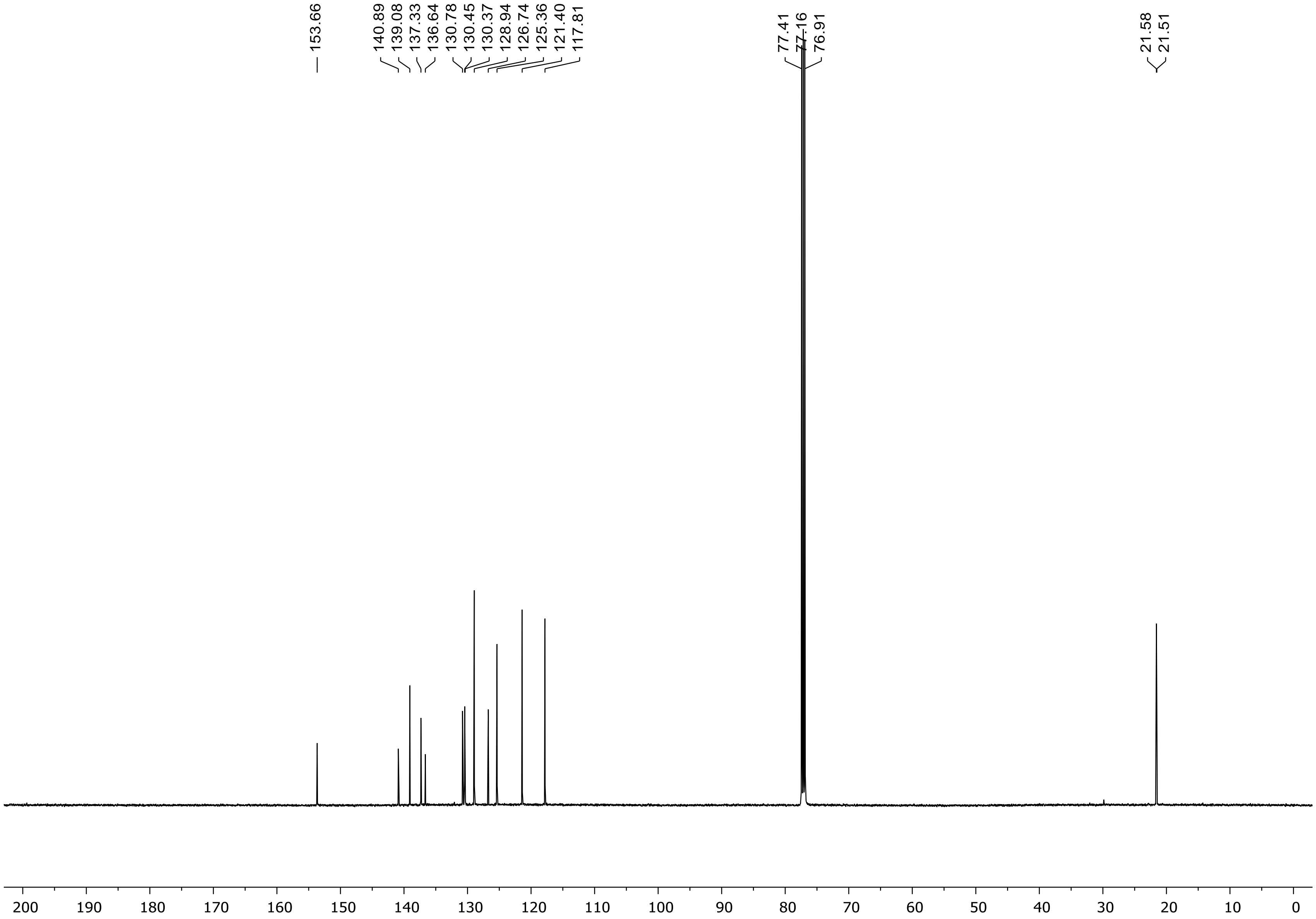

Figure S17: ^{1}**H NMR** (500 MHz, $CDCl_3$, 298 K) spectrum of **24**.

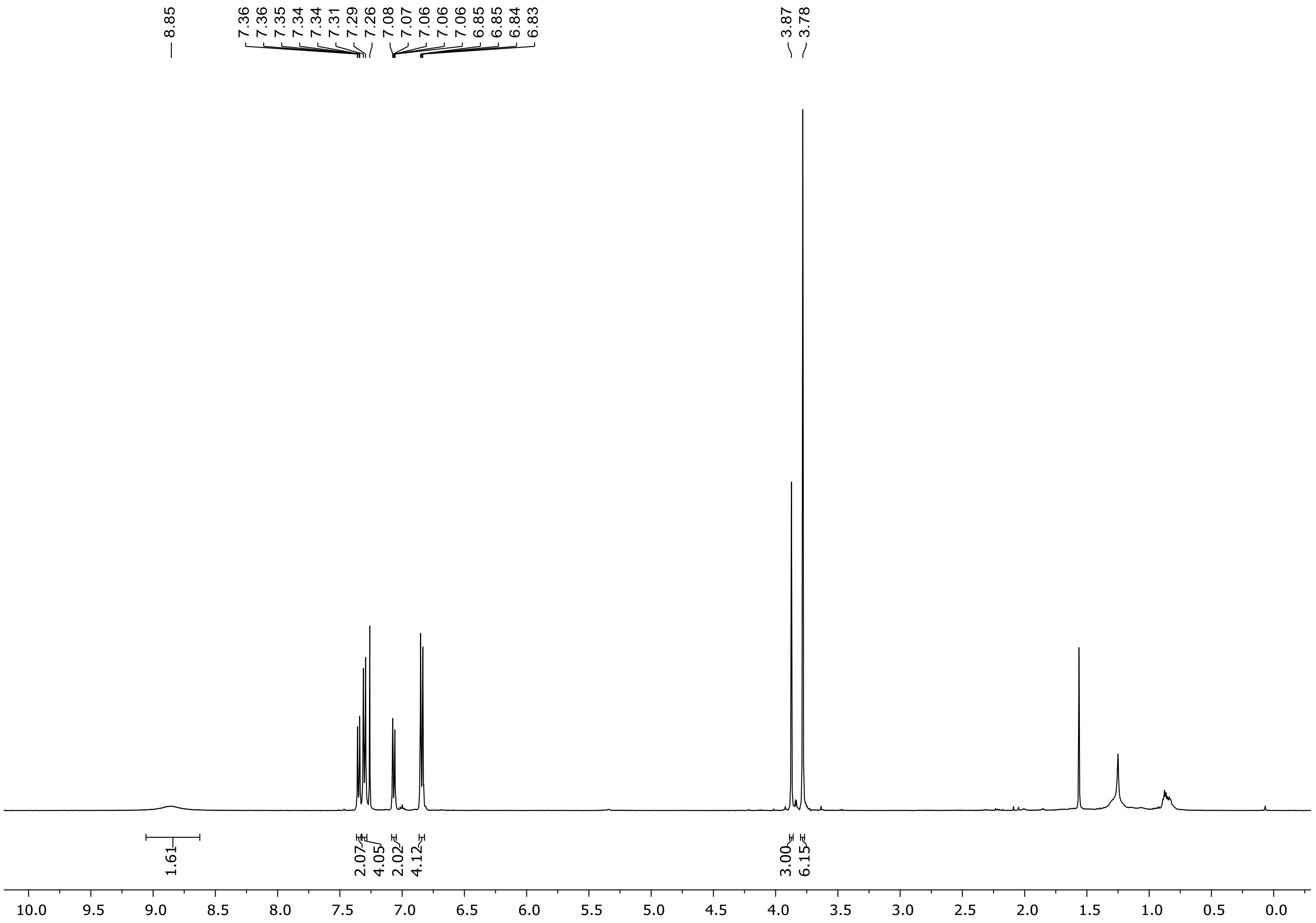

Figure S18: ^{13}**C NMR** (126 MHz, $CDCl_3$, 298 K) spectrum of **24**.

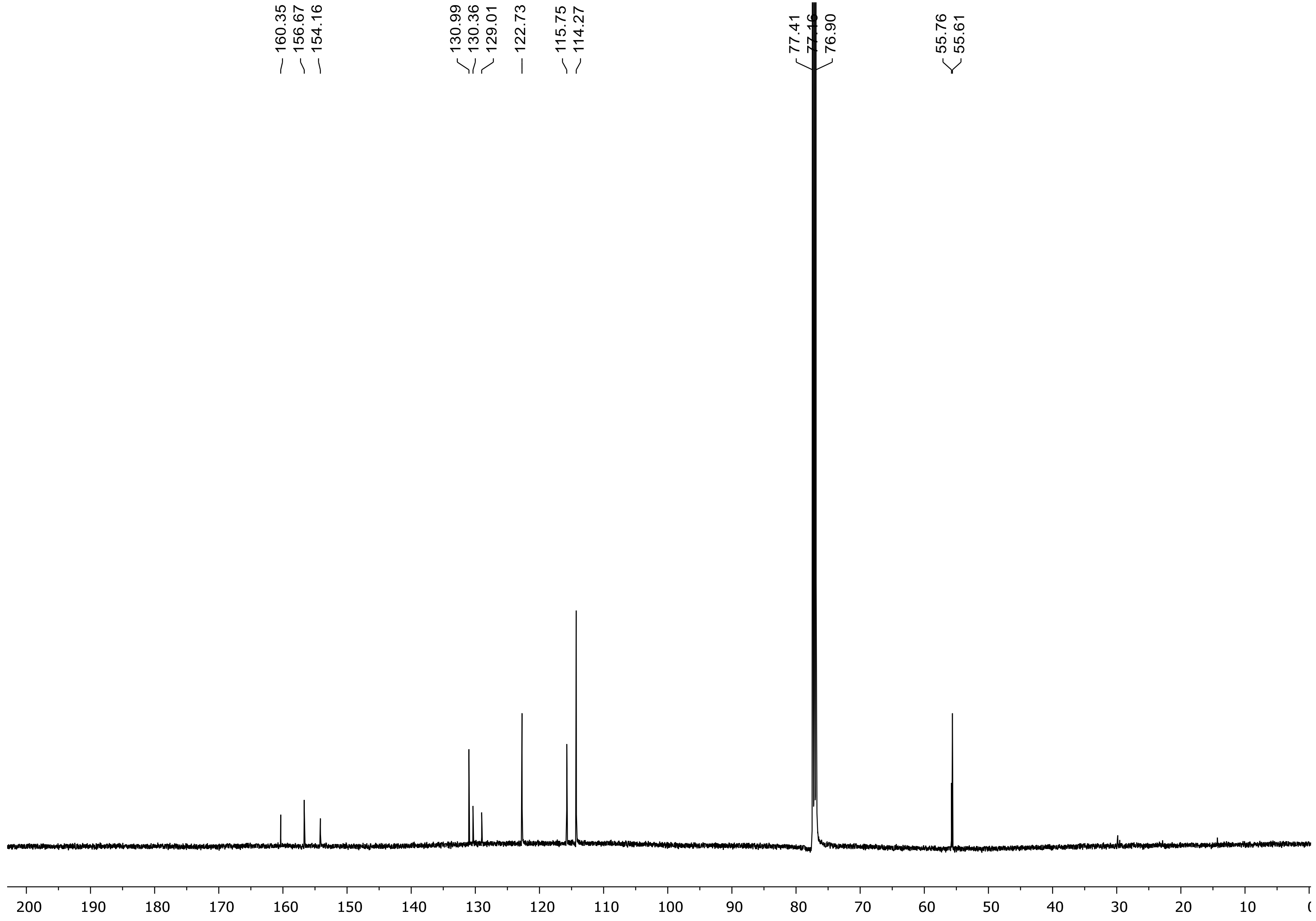

Figure S19: **^{1}H NMR** (500 MHz, CD_3CN, 298 K) spectrum of **25**.

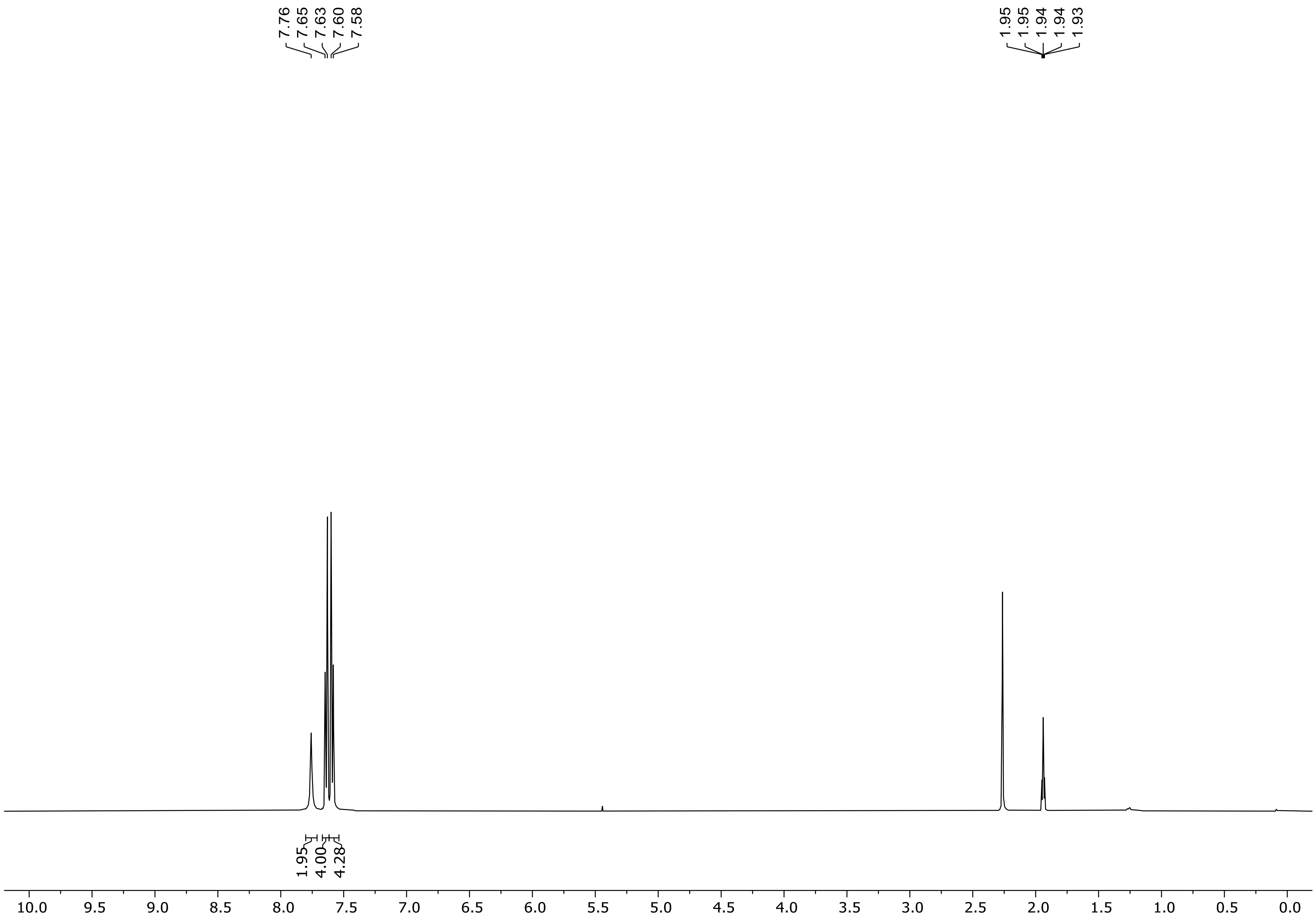

Figure S20: **^{13}C NMR** (126 MHz, CD_3CN, 298 K) spectrum of **25**.

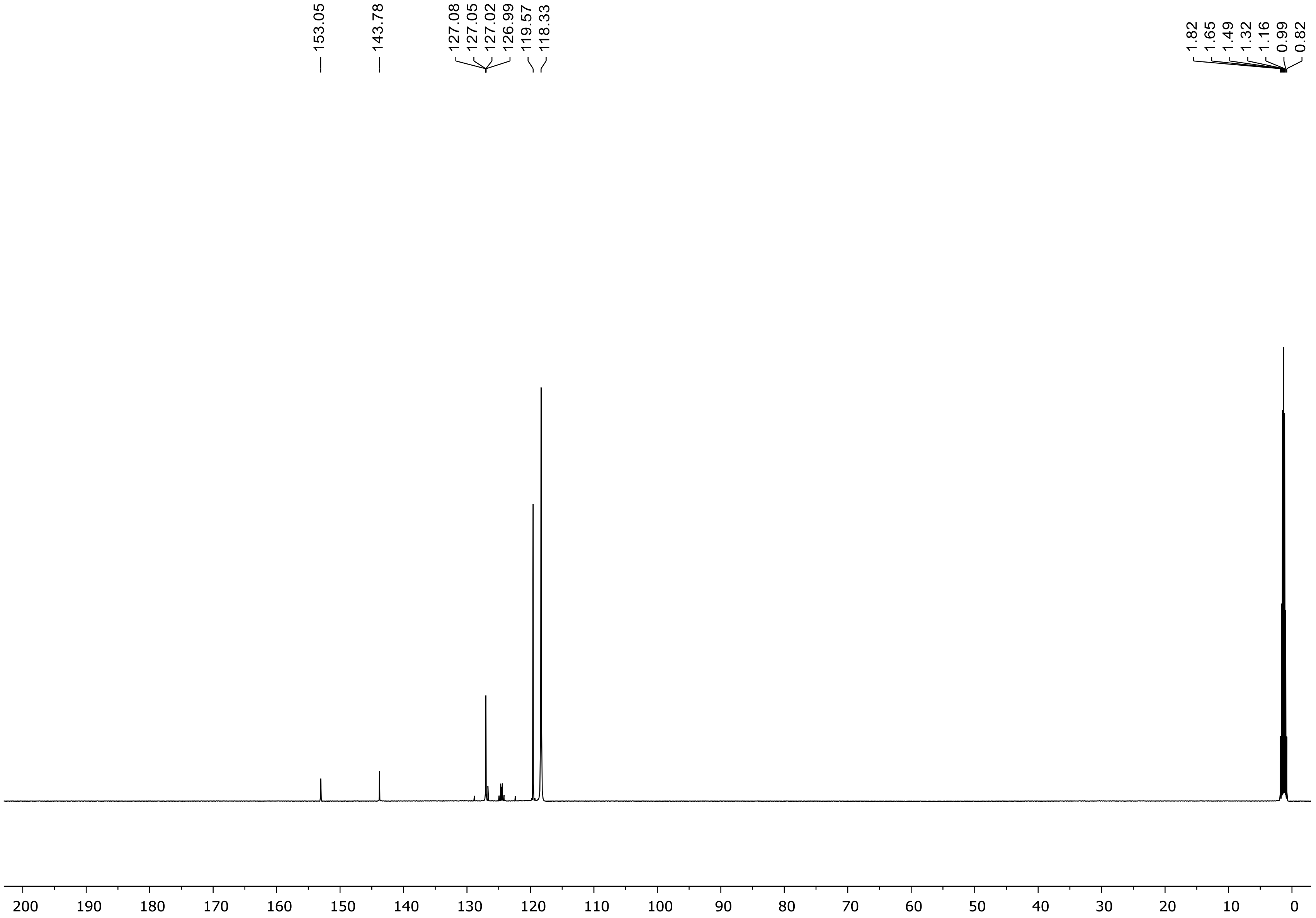

Figure S21: **^{19}F NMR** (471 MHz, CD_3CN, 298 K) spectrum of **25**.

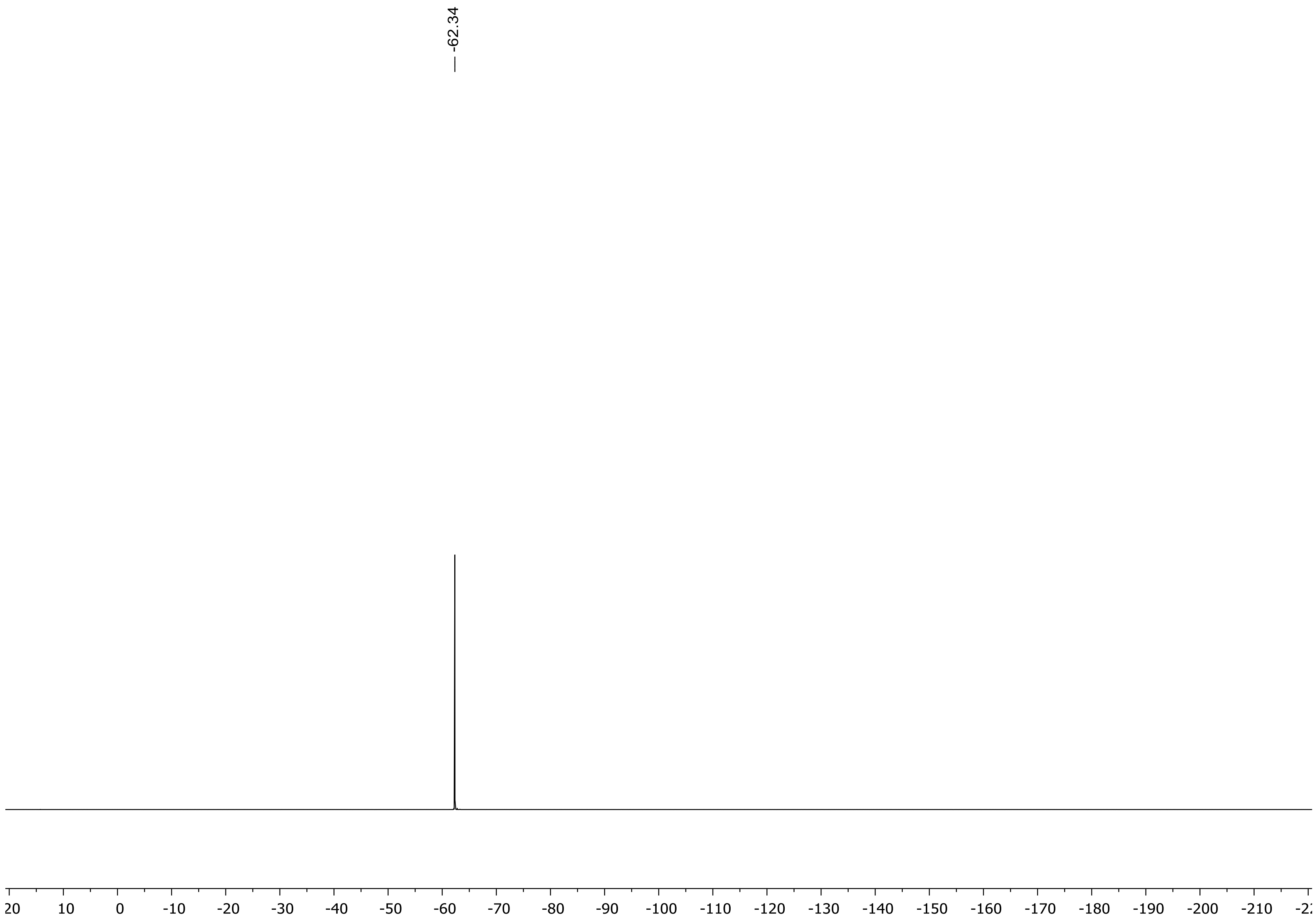

Figure S22: ^{1}**H NMR** (400 MHz, CD_3OD, 298 K) spectrum of **26**.

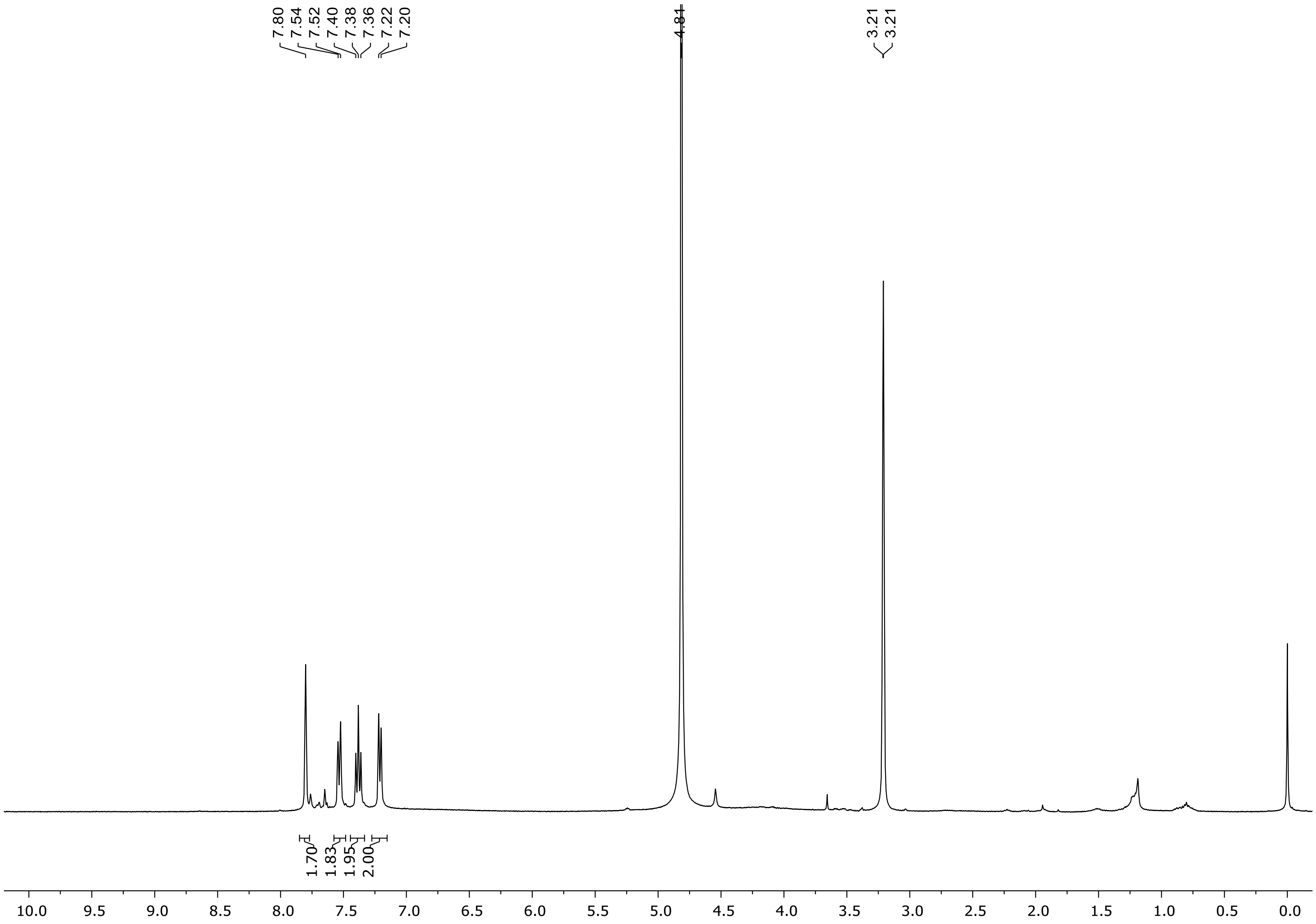

Figure S23: **^{13}C NMR** (126 MHz, CD_3OD, 298 K) spectrum of **26**.

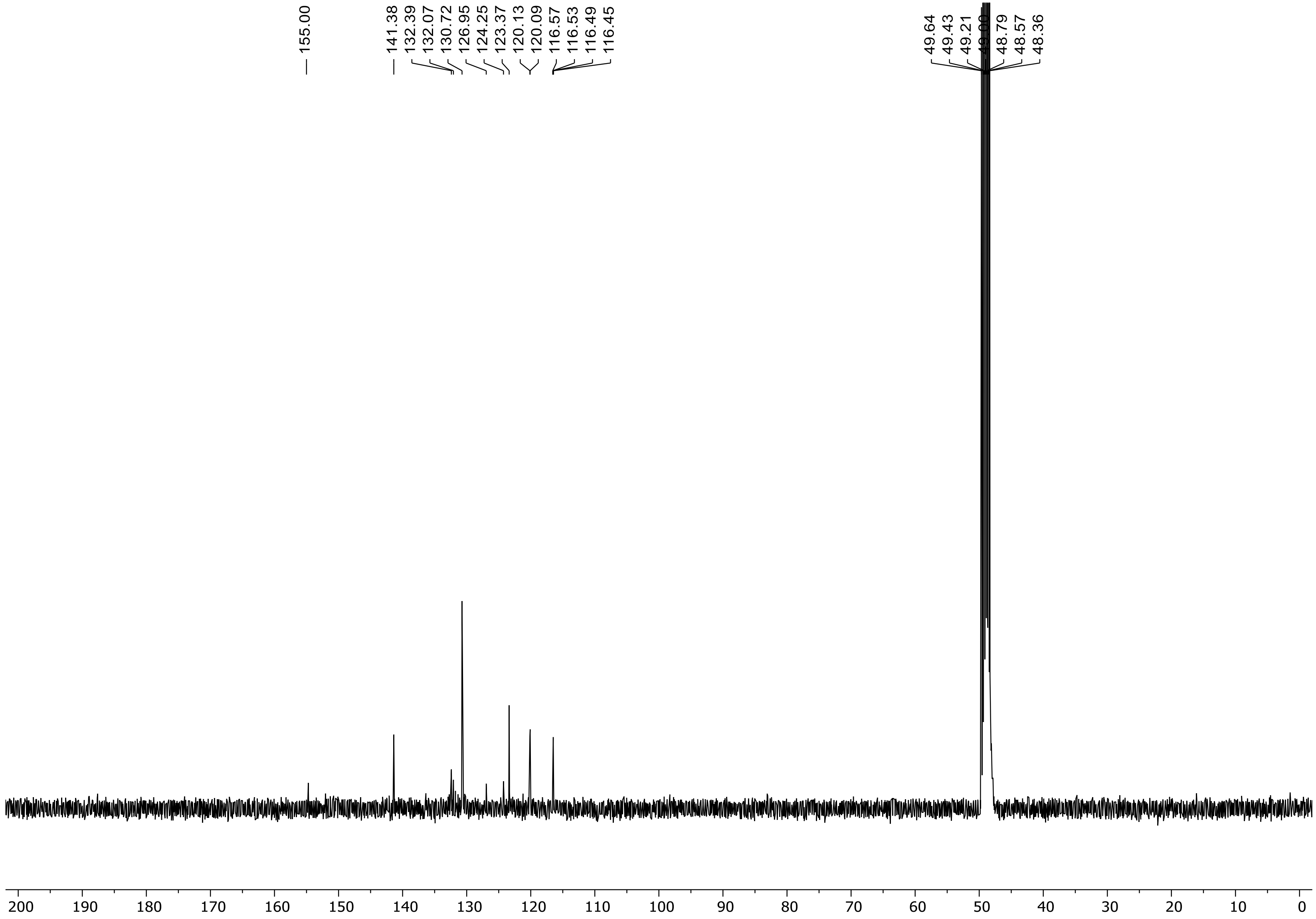

Figure S24: **^{19}F NMR** (376 MHz, CD_3OD, 298 K) spectrum of **26**.

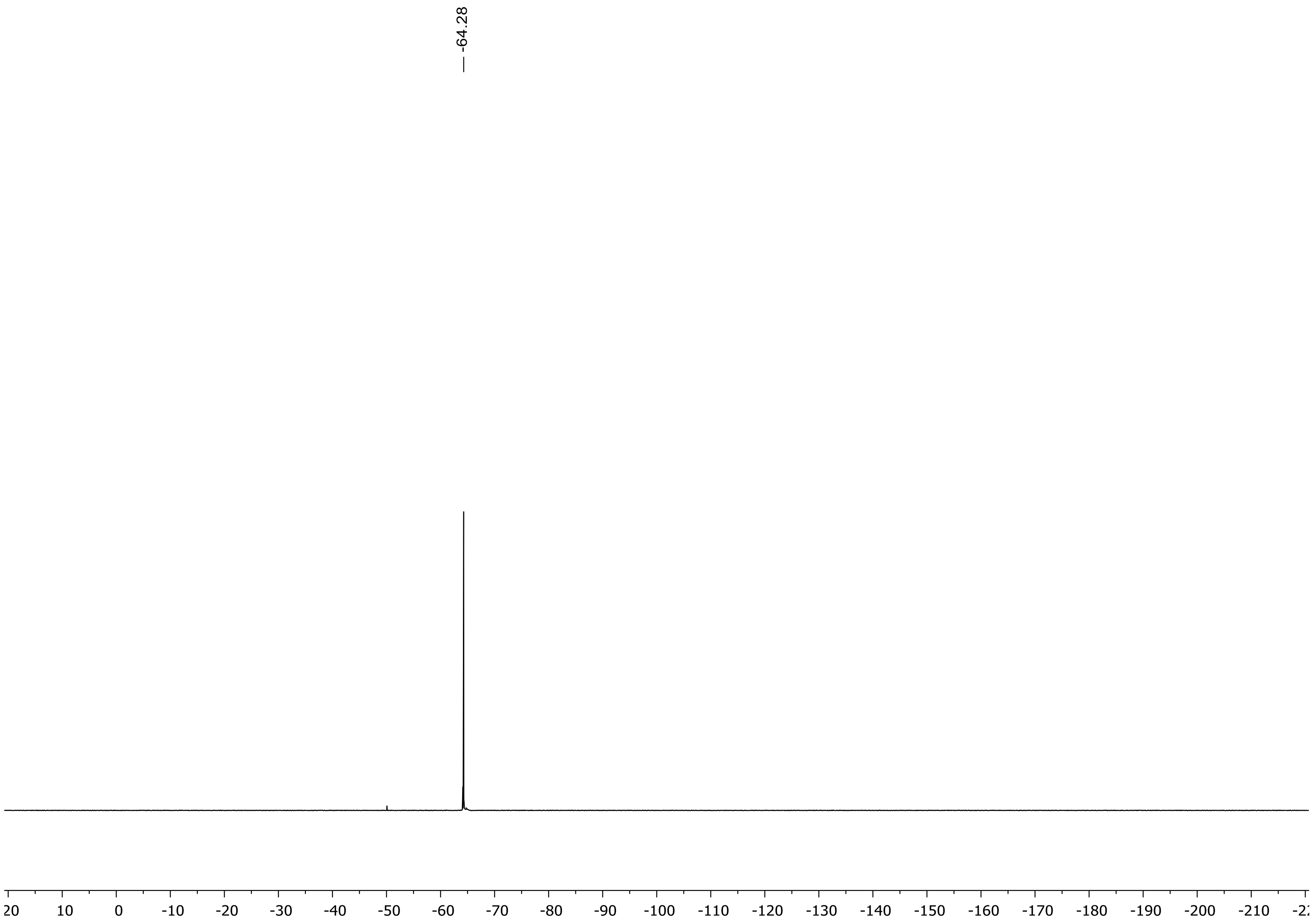

Figure S25: **^{1}H NMR** (500 MHz, DMSO-d_6, 298 K) spectrum of **27**.

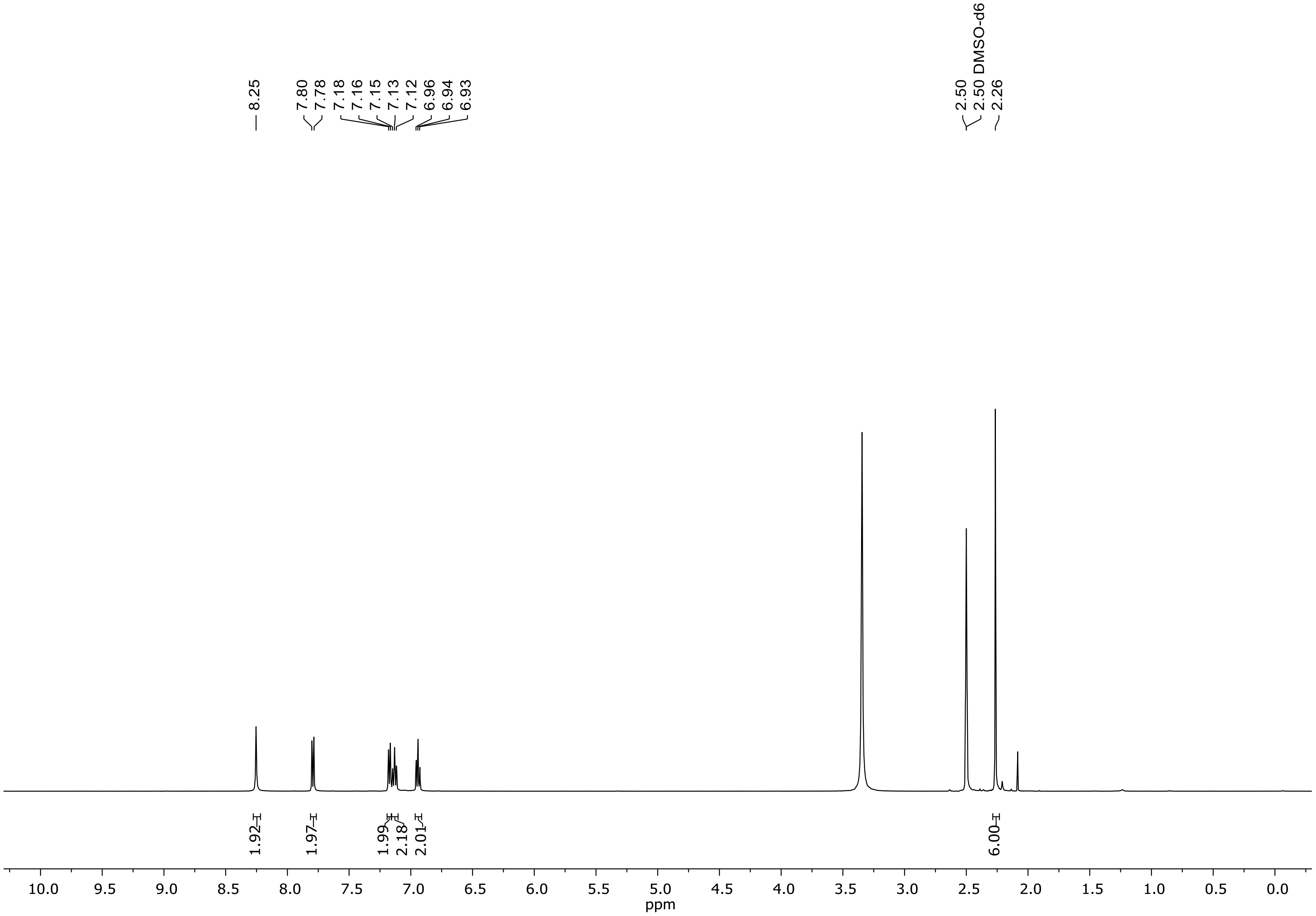

Figure S26: **^{13}C NMR** (126 MHz, DMSO-d_6, 298 K) spectrum of **27**.

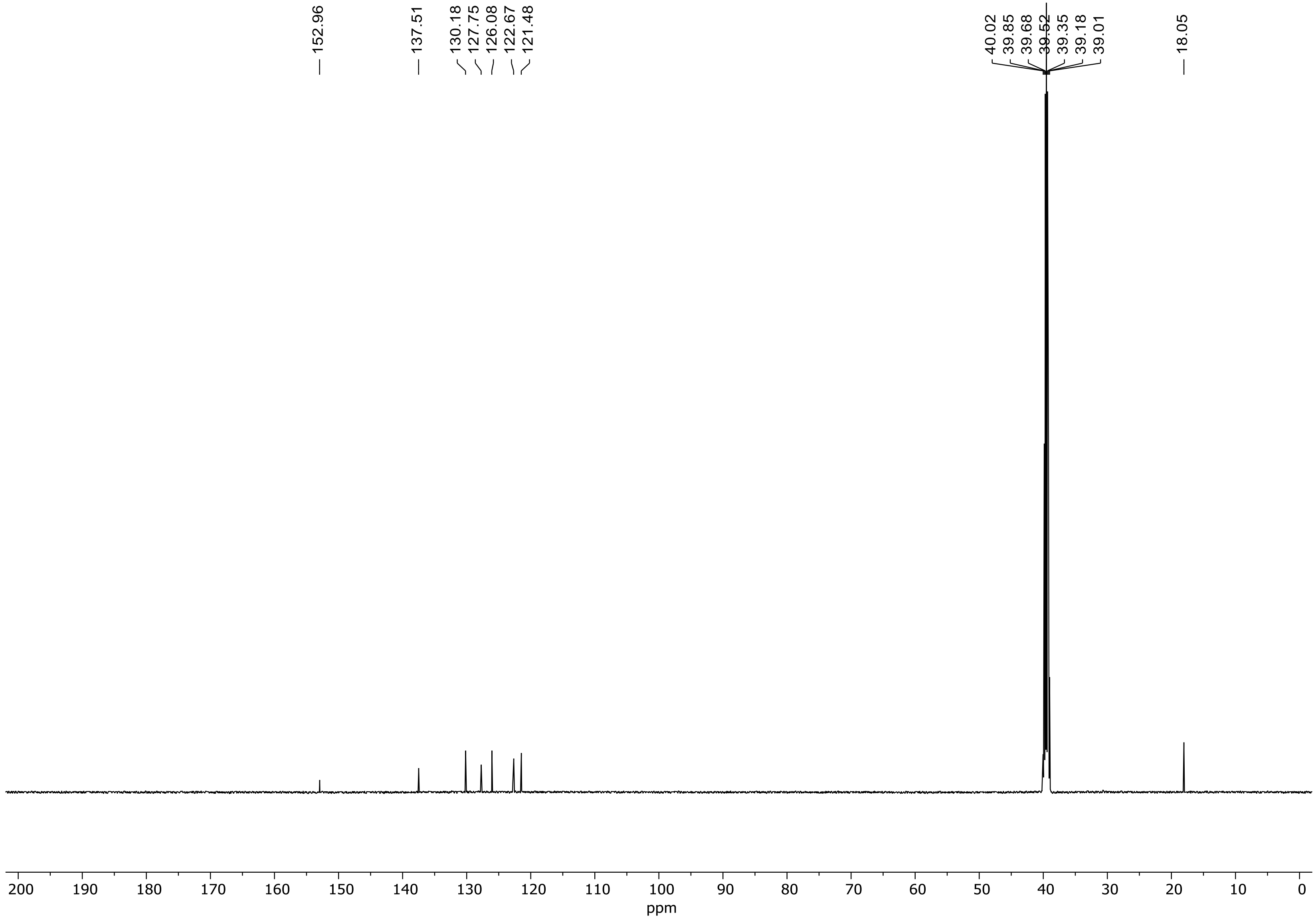

Figure S27: **^{1}H NMR** (500 MHz, CD_2Cl_2, 298 K) spectrum of **27a**.

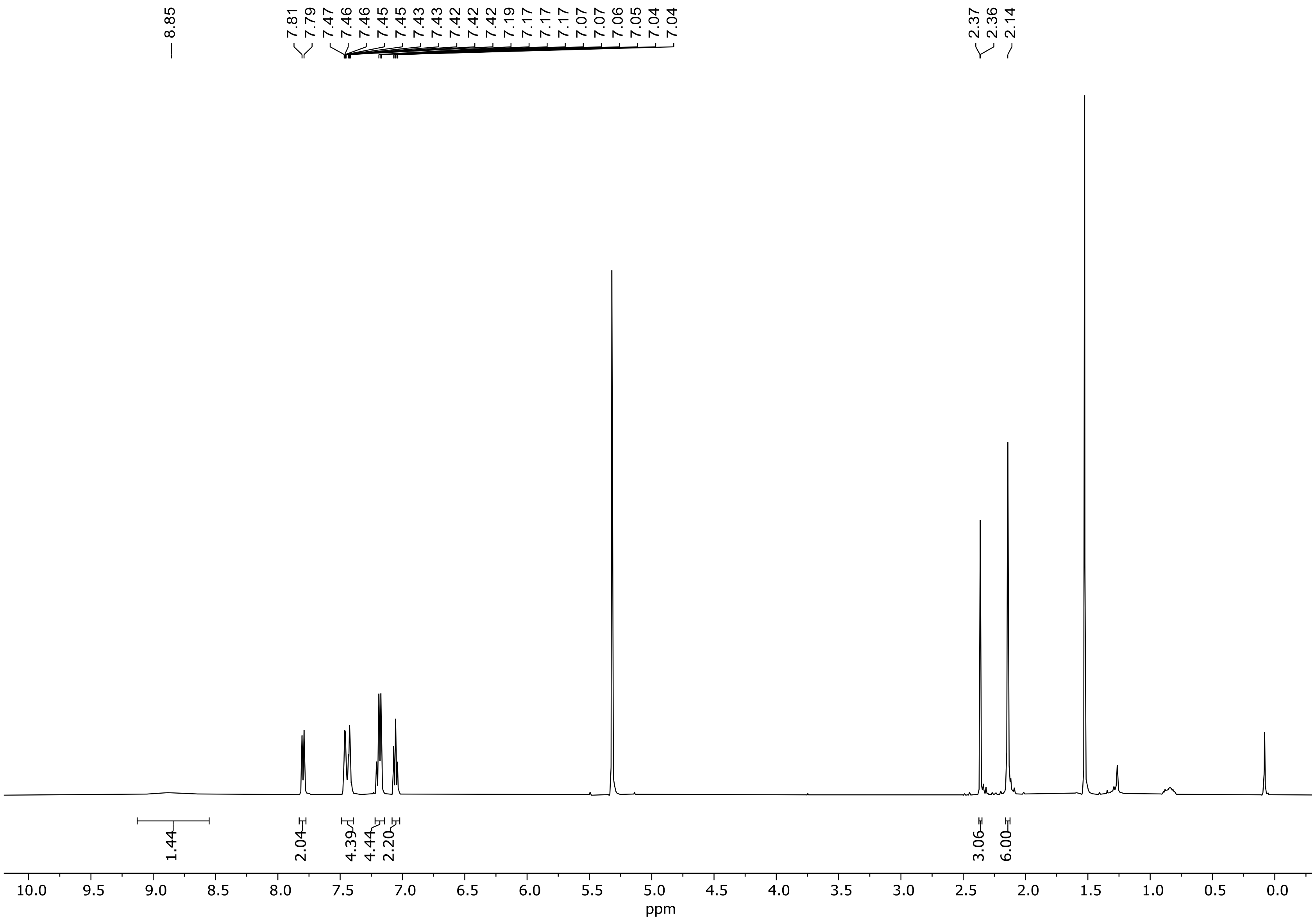

Figure S28: **^{13}C NMR** (126 MHz, CD_2Cl_2, 298 K) spectrum of **27a**.

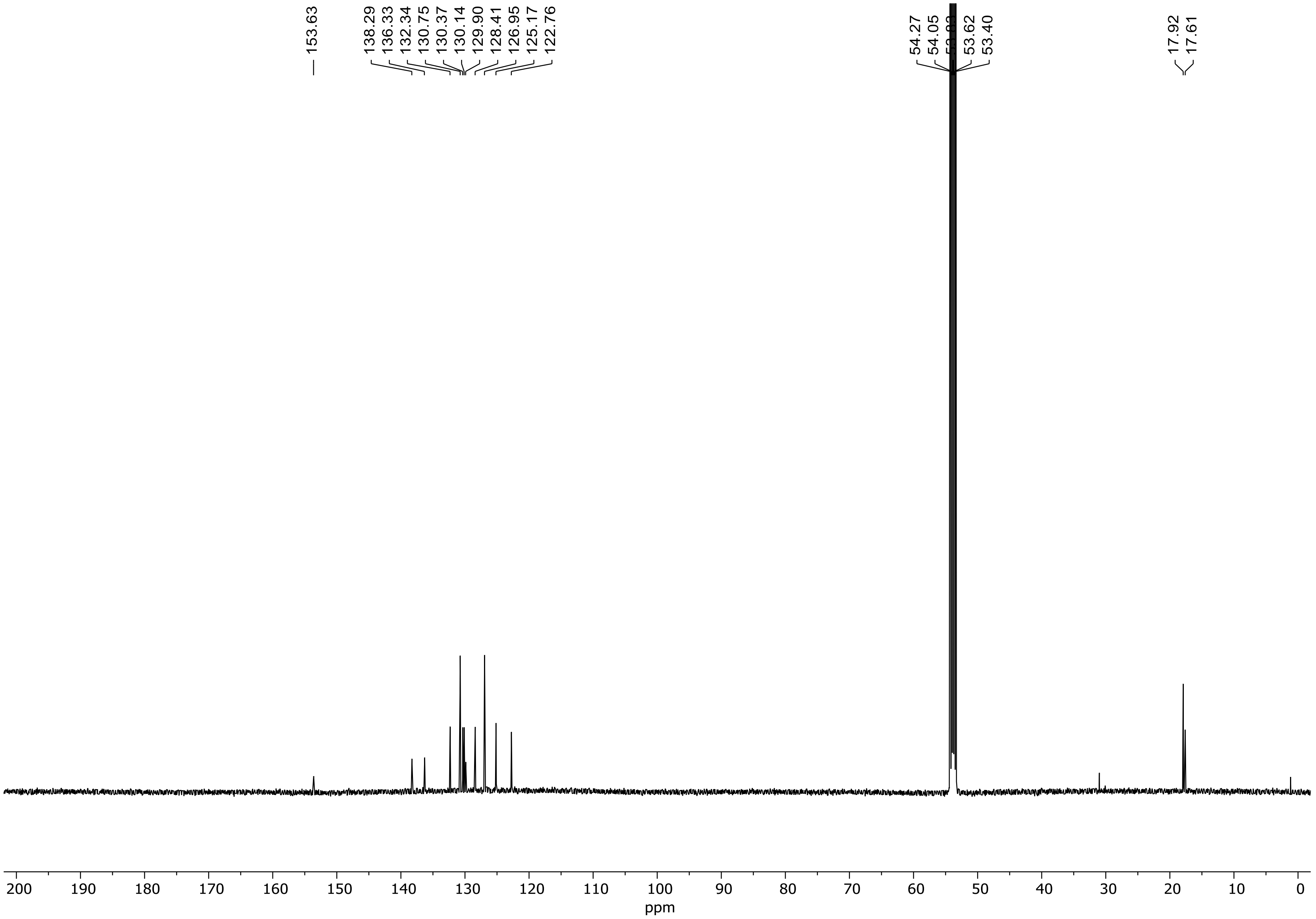

Figure S29: **^{1}H NMR** (500 MHz, CD_3OD, 298 K) spectrum of **28**.

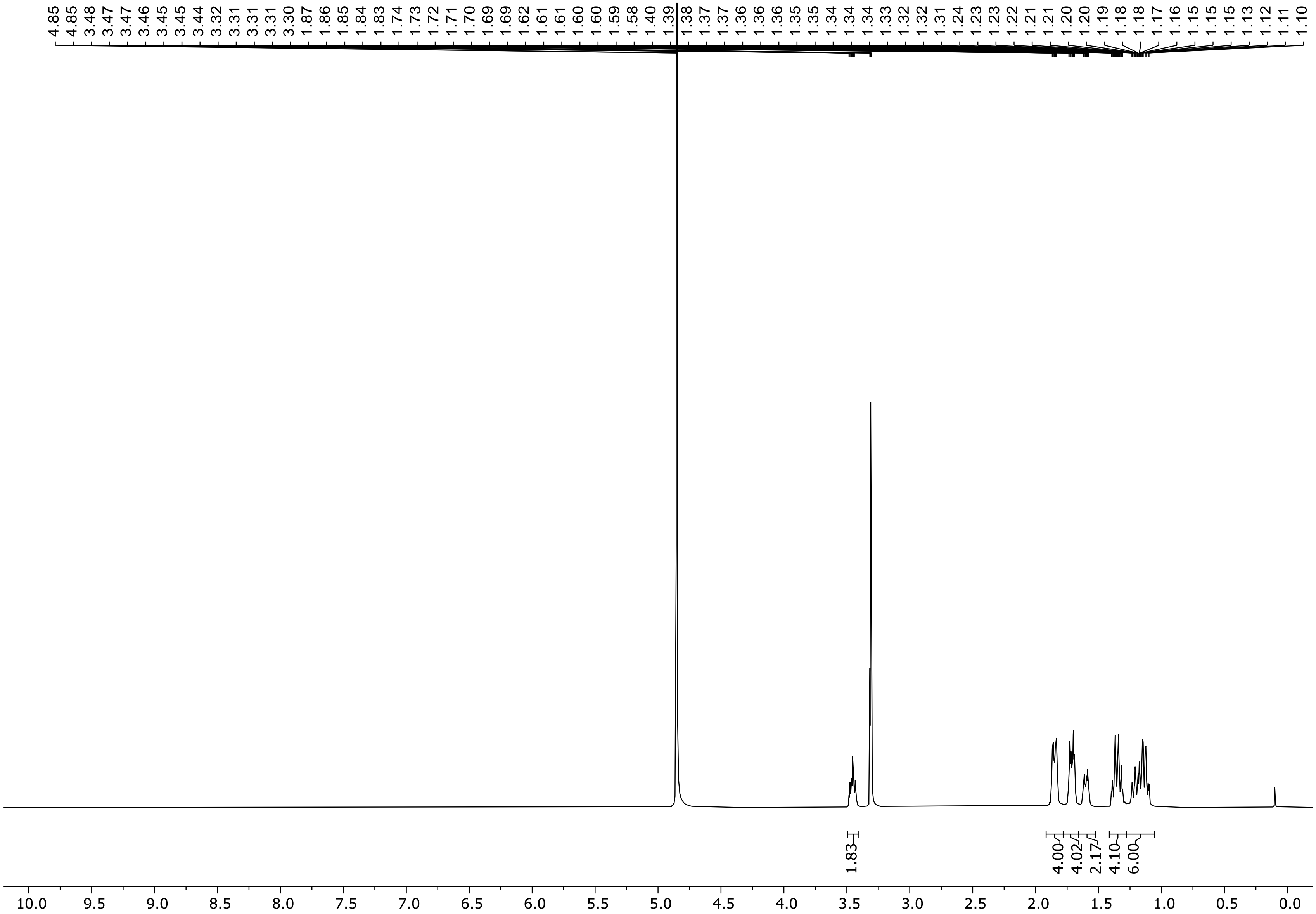

Figure S30: **^{13}C NMR** (126 MHz, CD_3OD, 298 K) spectrum of **28**.

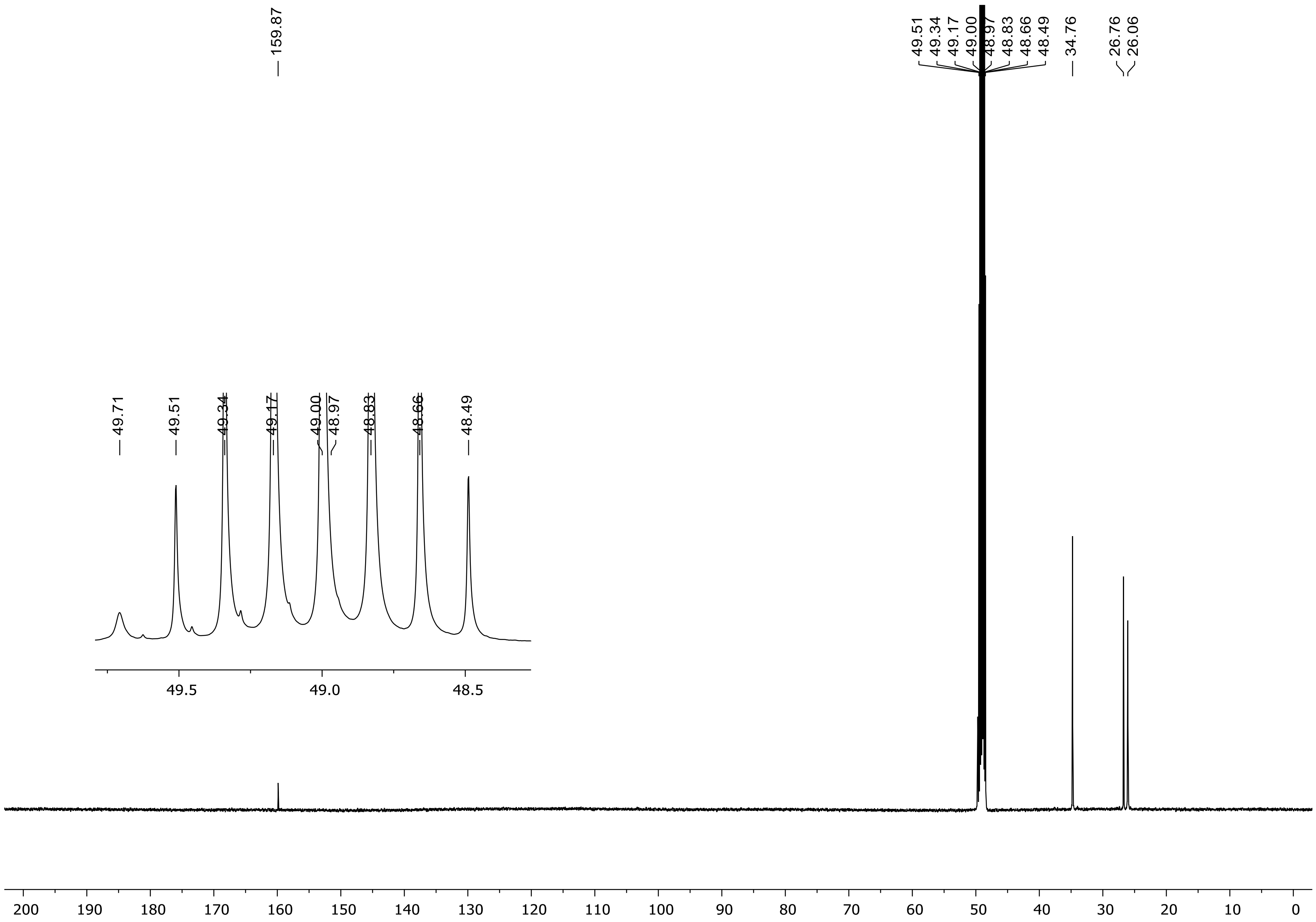

Figure S31: **^{1}H NMR** (500 MHz, $CDCl_3$, 298 K) spectrum of **29**.

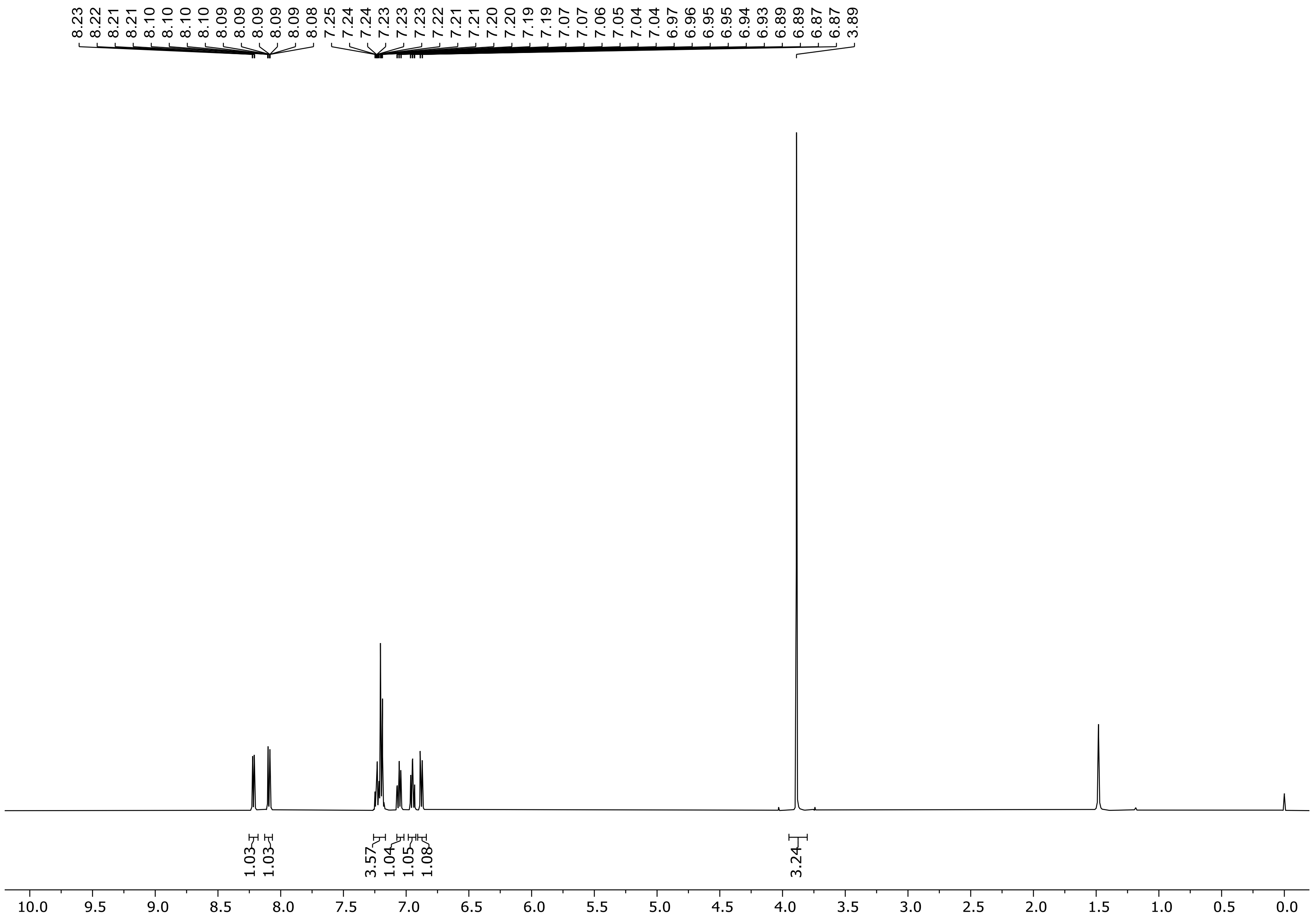

Figure S32: **^{13}C NMR** (126 MHz, $CDCl_3$, 298 K) spectrum of **29**.

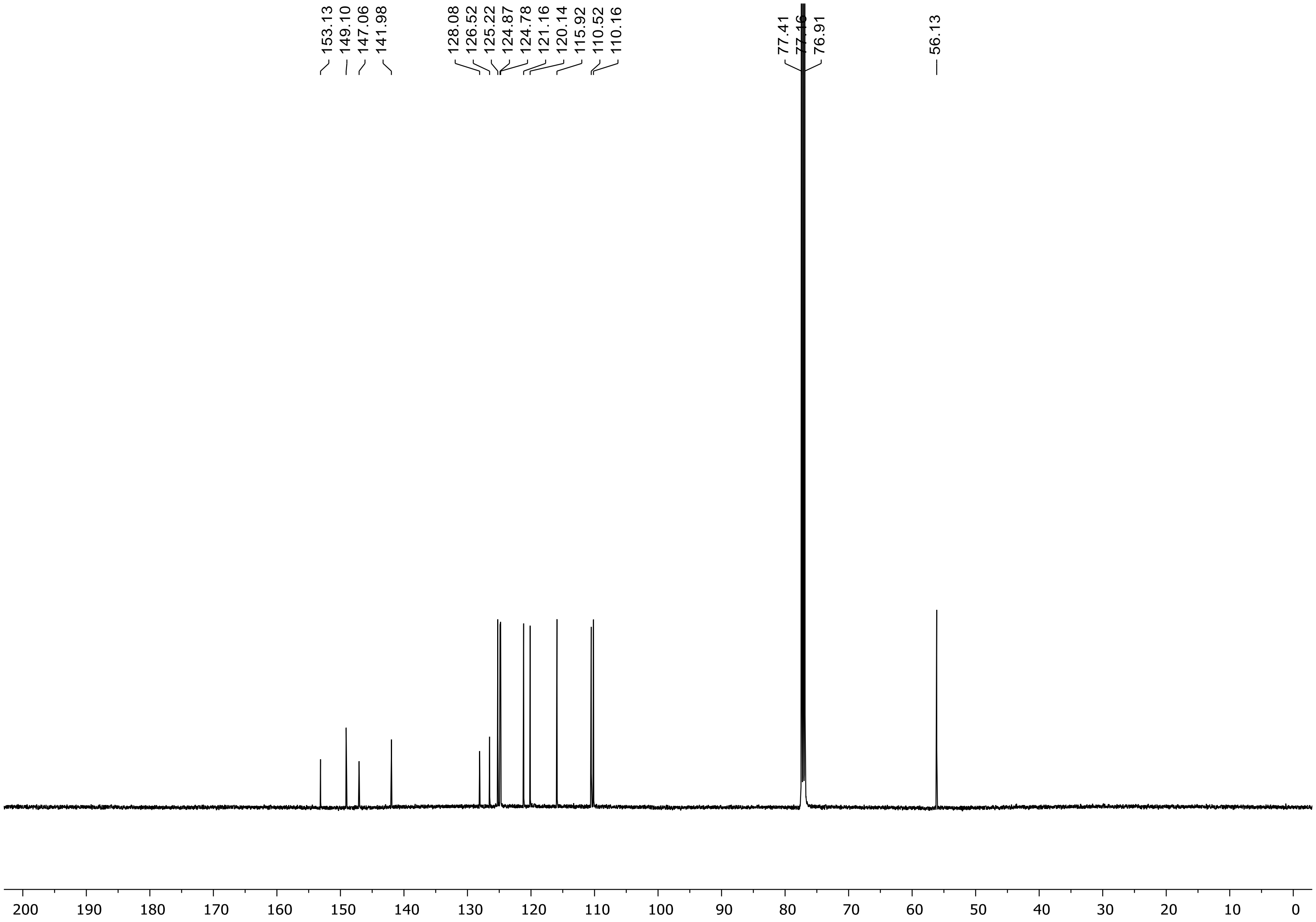

Figure S33: **^{1}H NMR** (500 MHz, $CDCl_3$, 298 K) spectrum of **30a**.

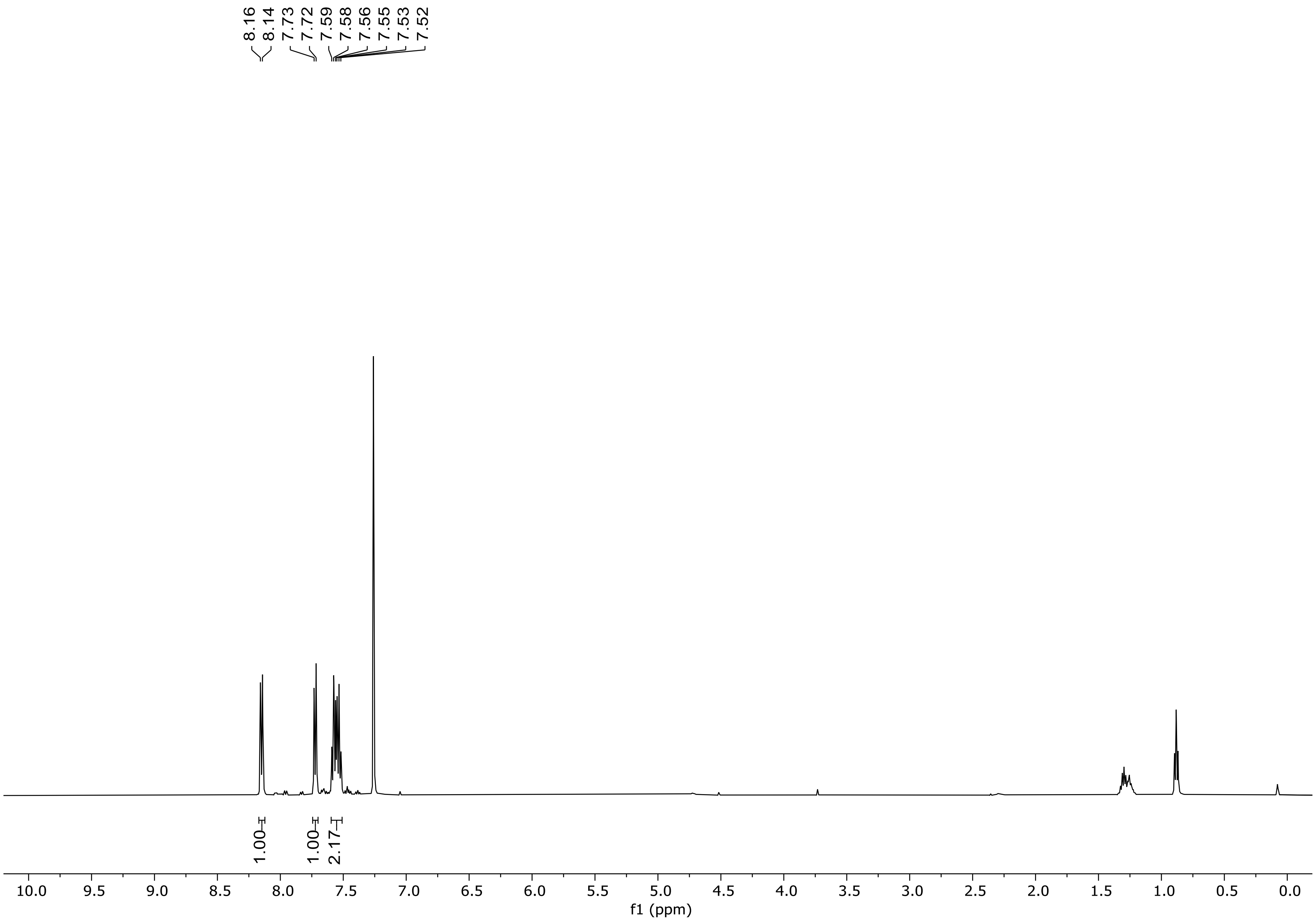

Figure S34: **^{13}C NMR** (126 MHz, $CDCl_3$, 298 K) spectrum of **30a**.*

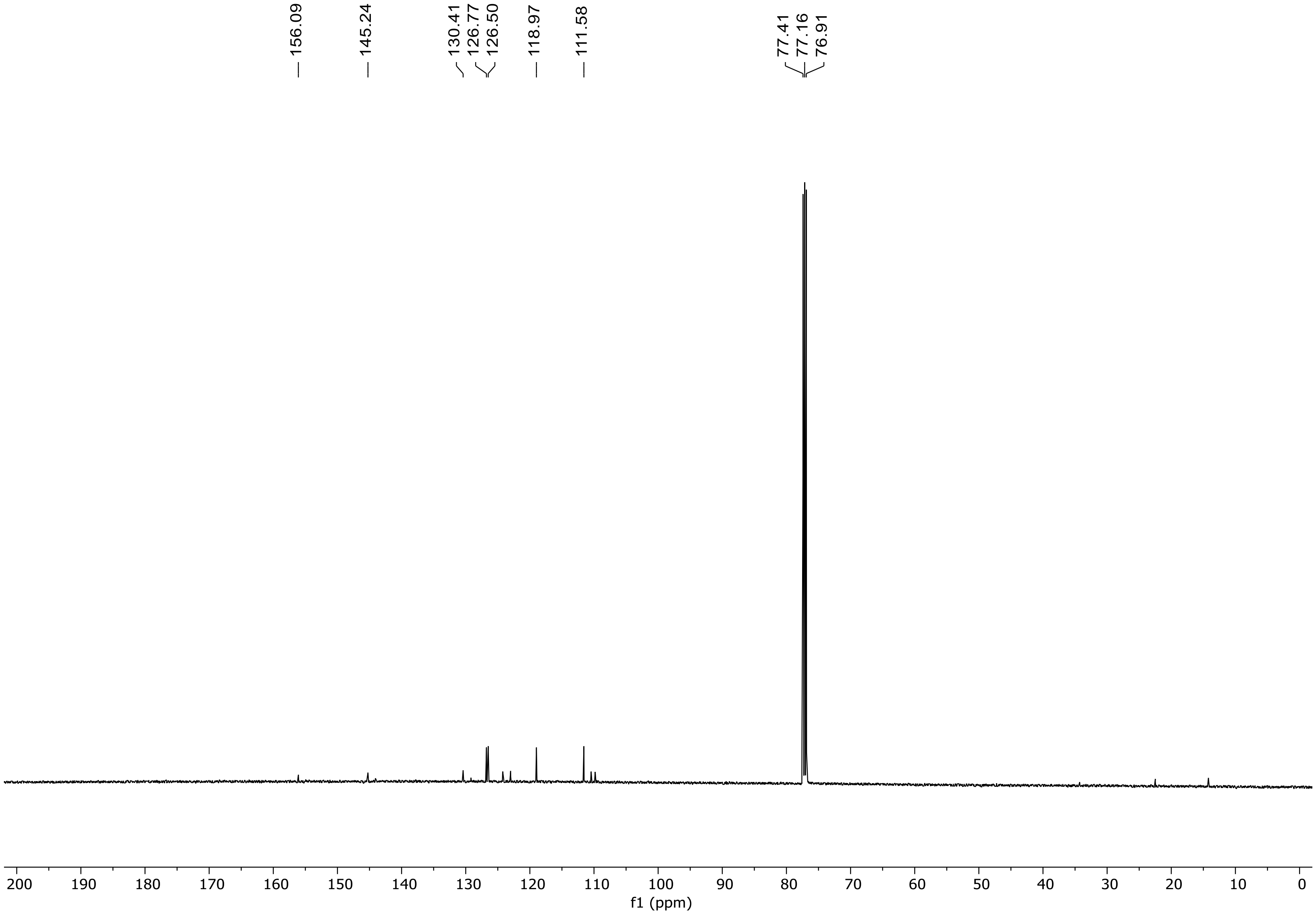

* **30a** was found to be unstable in solution, decomposing to form hydrolysed product **30** as evidenced from Figure S35 below.

Figure S35. Stacked **^{13}C NMR** (126 MHz, $CDCl_3$, 298 K) of **30a** (top) and **30** (bottom).

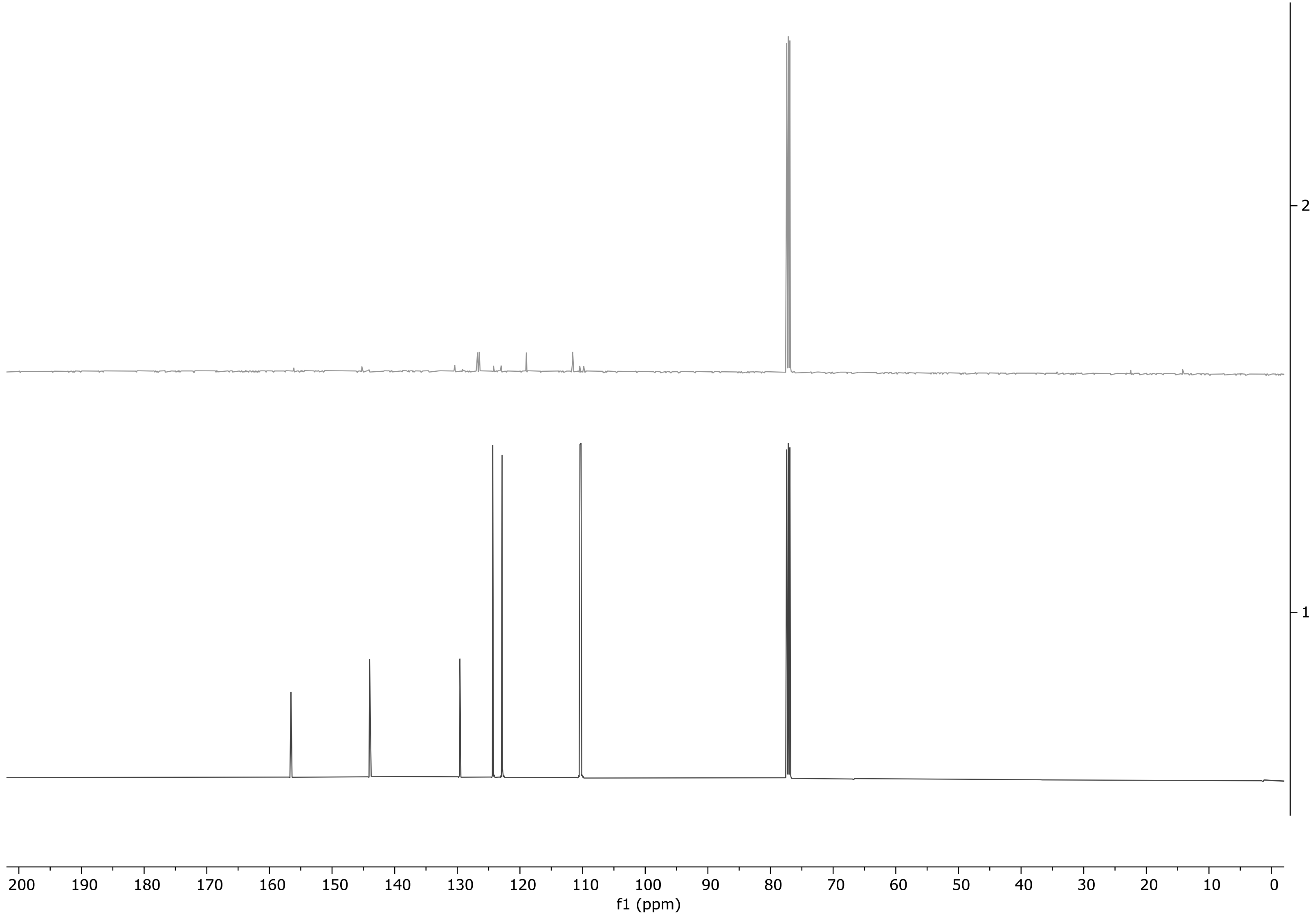

Figure S36: **^{11}B NMR** (160 MHz, $CDCl_3$, 298 K) spectrum of **30a**.

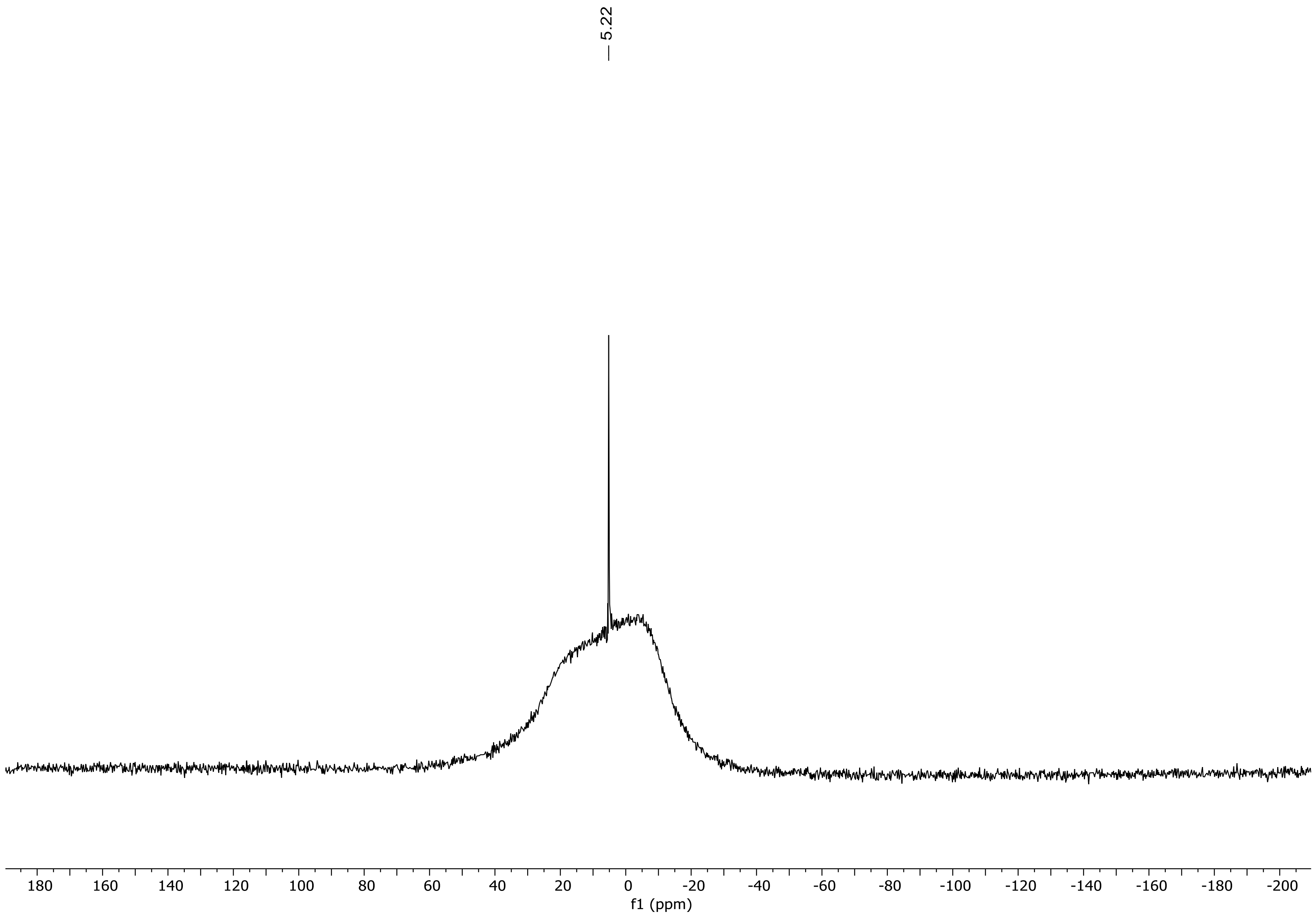

Figure S37: **^{1}H NMR** (500 MHz, $CDCl_3$, 298 K) spectrum of **30**.

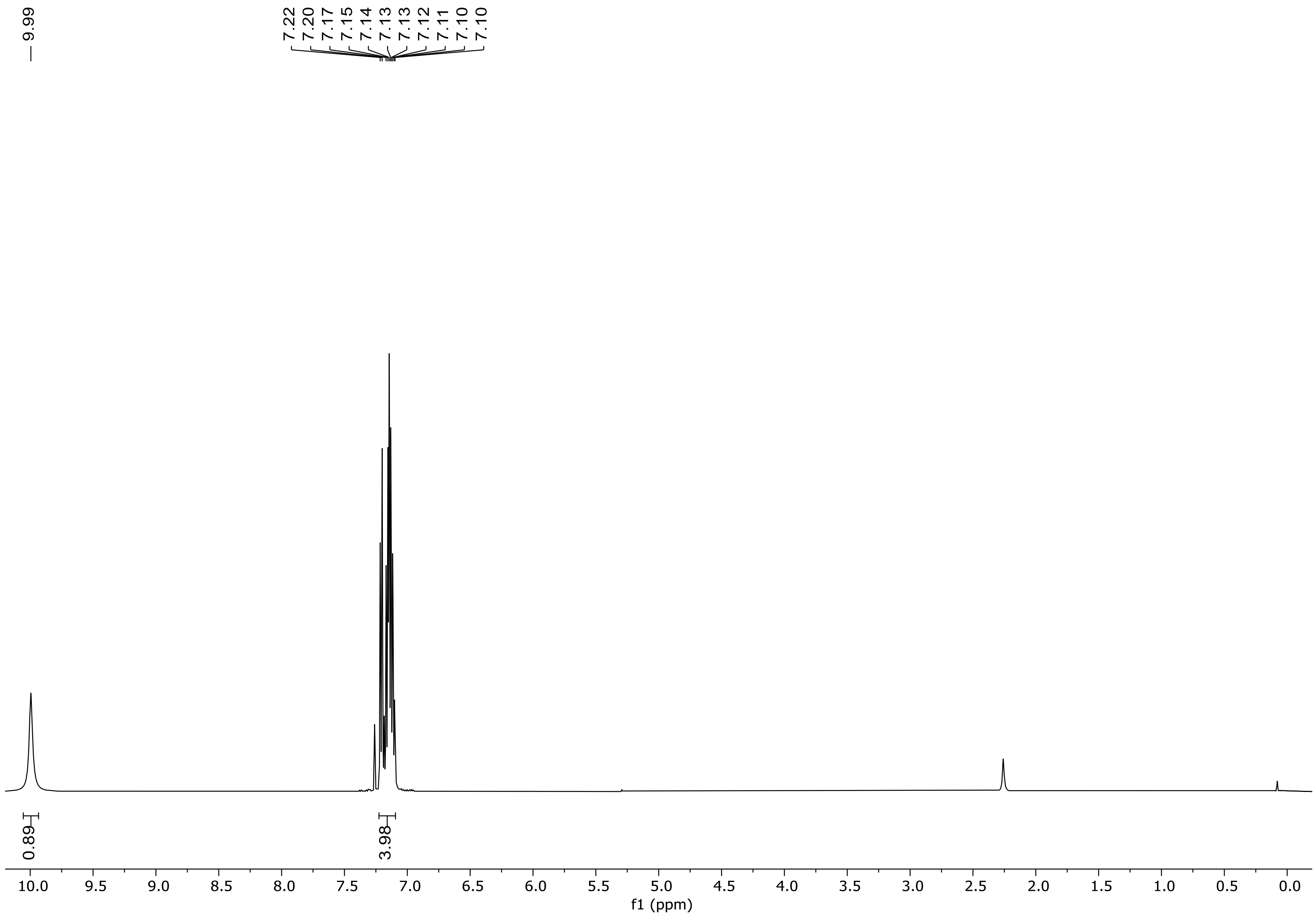

Figure S38: **^{13}C NMR** (126 MHz, $CDCl_3$, 298 K) spectrum of **30**.

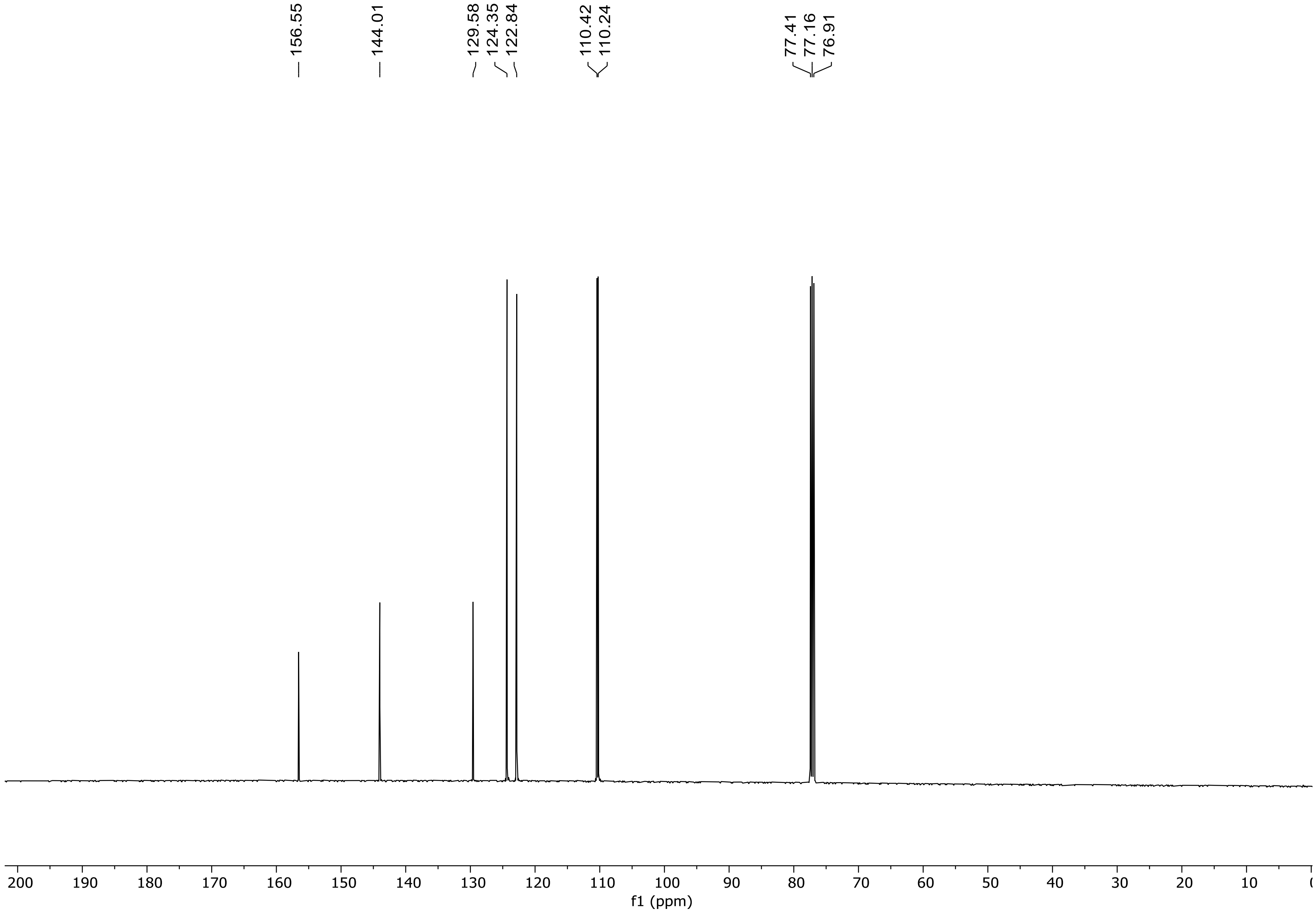

Figure S39: **^{1}H NMR** (500 MHz, $CDCl_3$, 298 K) spectrum of crude aniline reaction.

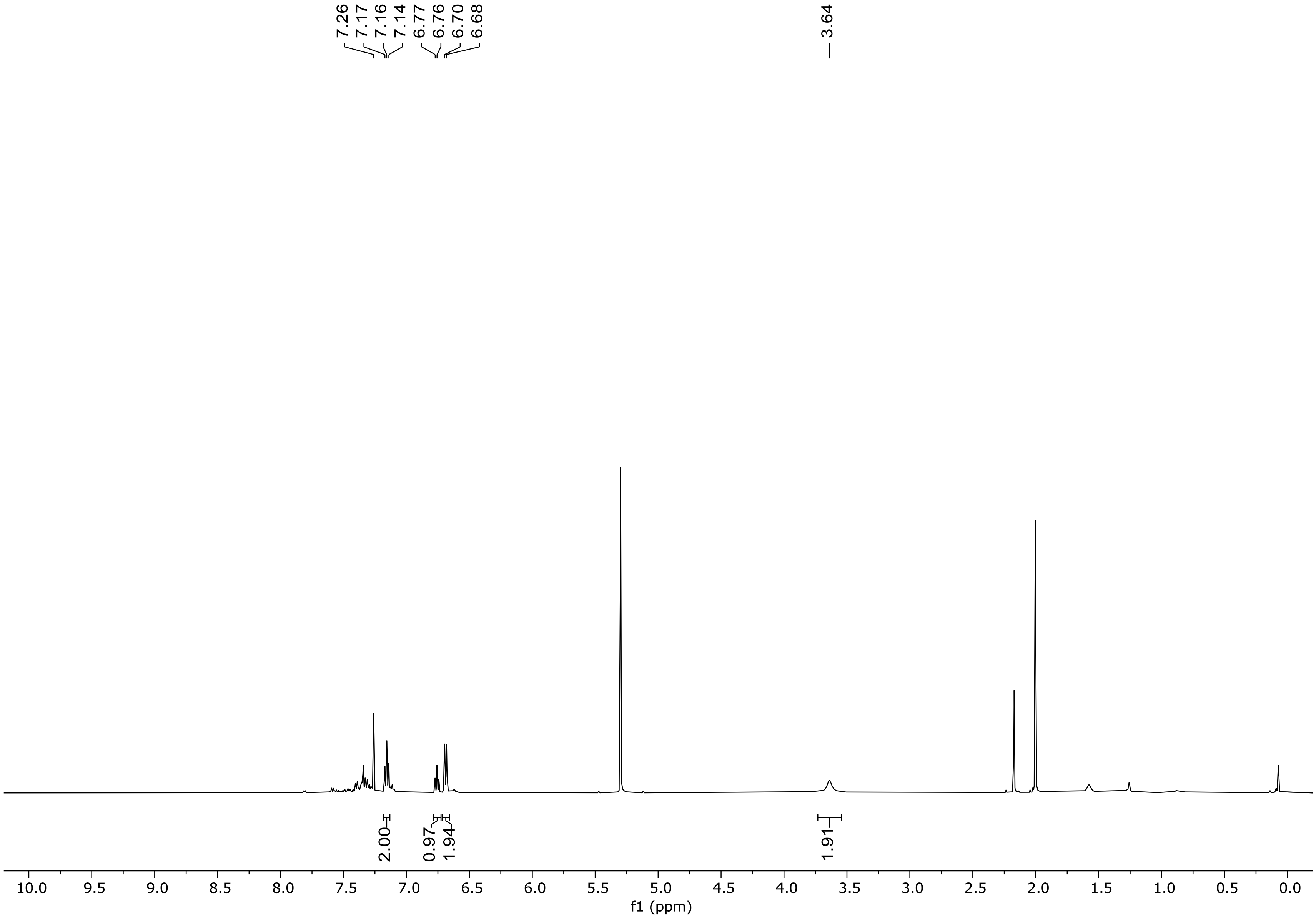

4. Crystallographic Data

4.1 Single crystal X-ray diffraction experimental:

Single crystals of **18, 20, 22**, and **25** were grown in a fume hood by slow evaporation or vapour diffusion. **20a** and **30a** were grown in a glovebox under a nitrogen atmosphere by slow evaporation at -30 ^{o}C. Crystallographic studies were undertaken on single crystal mounted in paratone and studied on an Agilent SuperNova Dual Atlas three-circle diffractometer using Mo- or Cu-Kα radiation and a CCD detector. Measurements were taken at 180(1) K with temperatures maintained using an Oxford cryostream. Data were collected and integrated and data corrected for absorption using a numerical absorption correction based on Gaussian integration over a multifaceted crystal model within CrysAlisPro.[12] The structure was solved by direct methods and refined against F^2 within SHELXL-2013.[13] The structure has been deposited with the Cambridge Structural Database [CCDC deposition numbers **2125084** (**18**), **2128581** (**20**), **2160532** (**20a**), **2128580** (**22**), **2128579** (**25**), **2157033** (**29**), **2157032** (**30a**)]. This can be obtained free of charge from the Cambridge Crystallographic Data Centre via www.ccdc.cam.ac.uk/data_request/cif

4.2 Crystal structures

Figure S40: Crystal structure of compound **18**. Thermal ellipsoids drawn at 50% probability.[3] H atoms omitted for clarity. C atoms in black, N in blue, O in red.

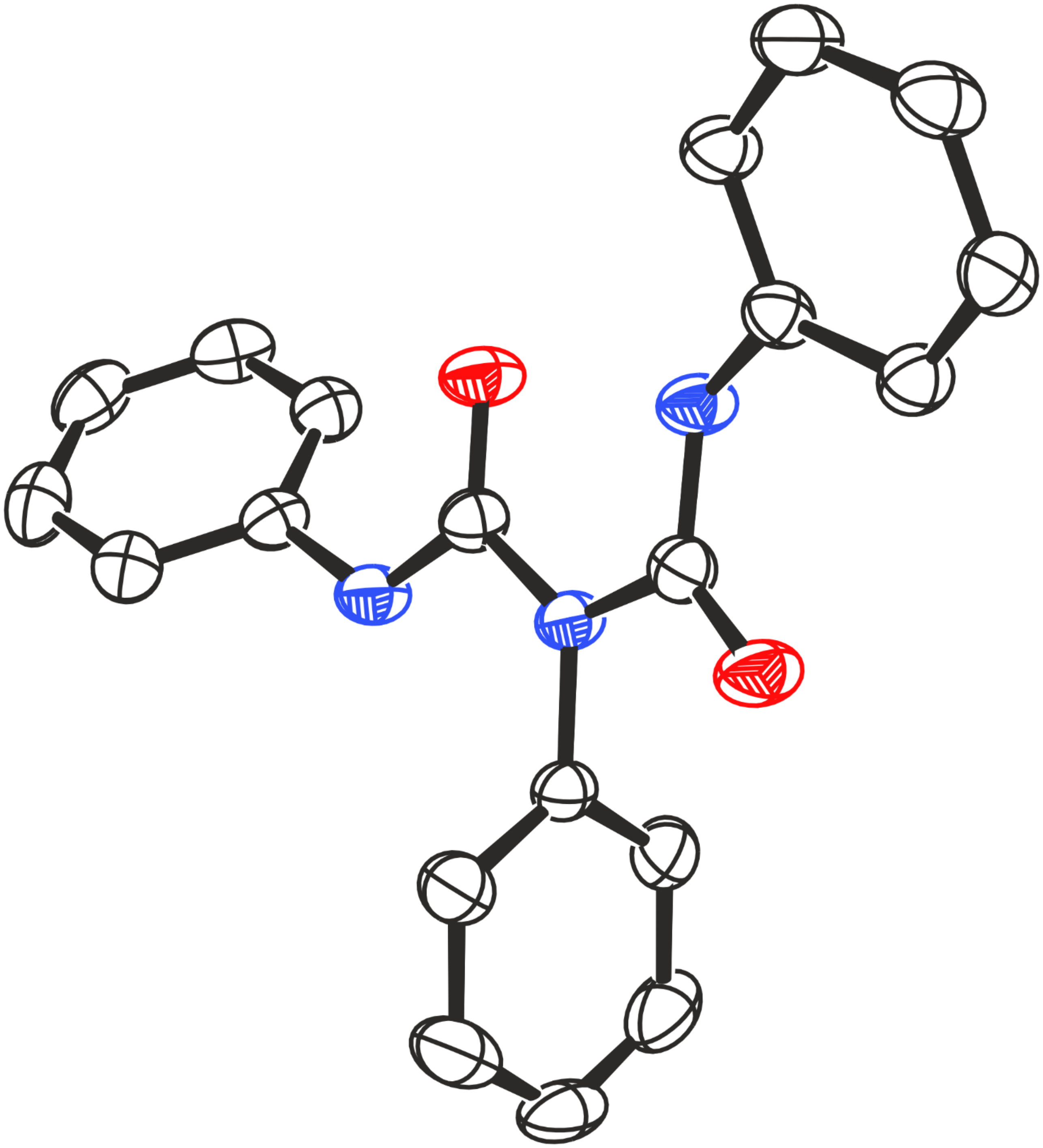

Figure S41: Crystal structure of compound **20**. Thermal ellipsoids drawn at 50% probability.[14] H atoms omitted for clarity. C atoms in black, N in blue, O in red, Cl in green.

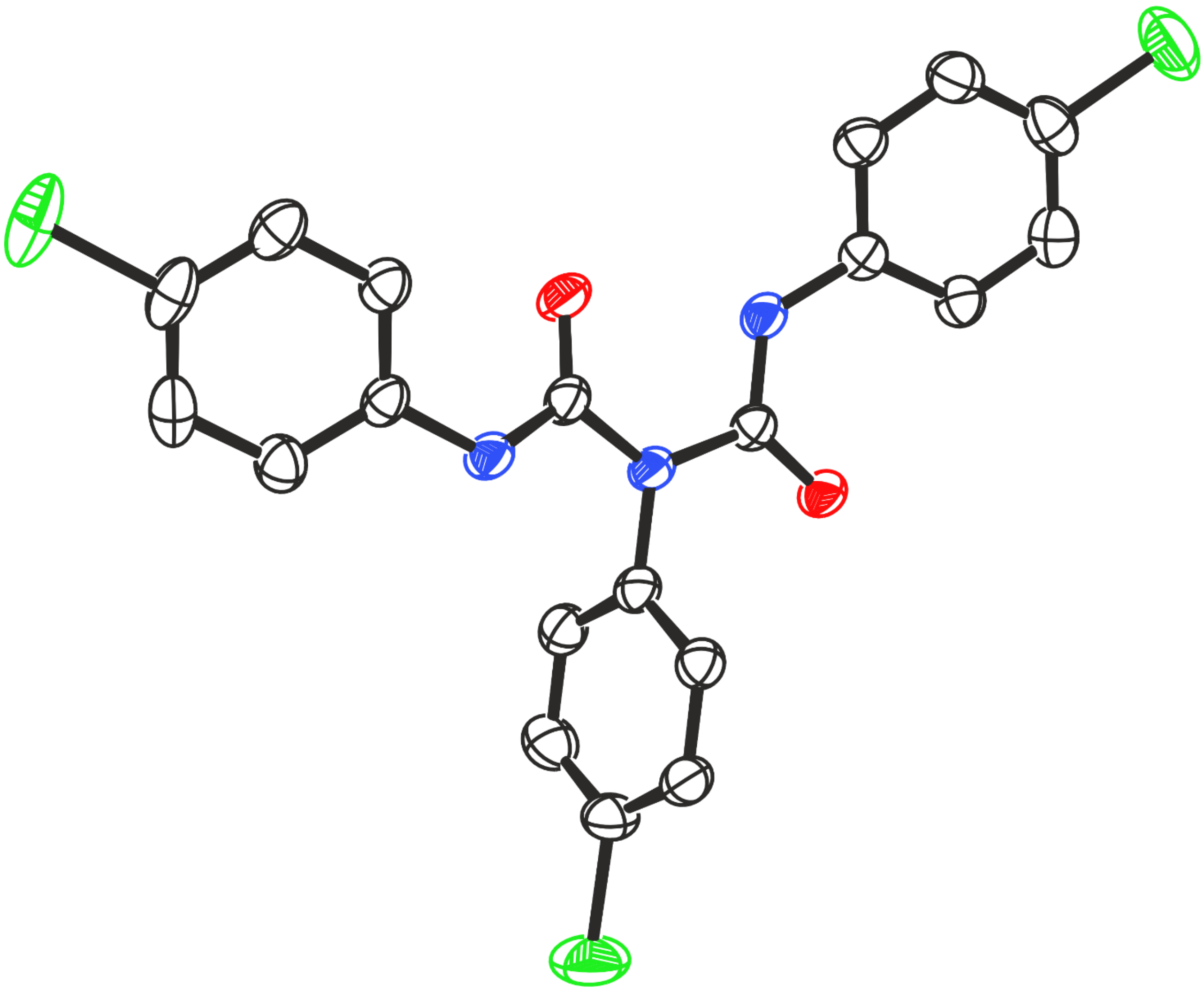

Figure S42: Crystal structure of compound **20a**. Thermal ellipsoids drawn at 50% probability. H atoms omitted for clarity. C atoms in black, N in blue, O in red, Cl in green, B in pink.

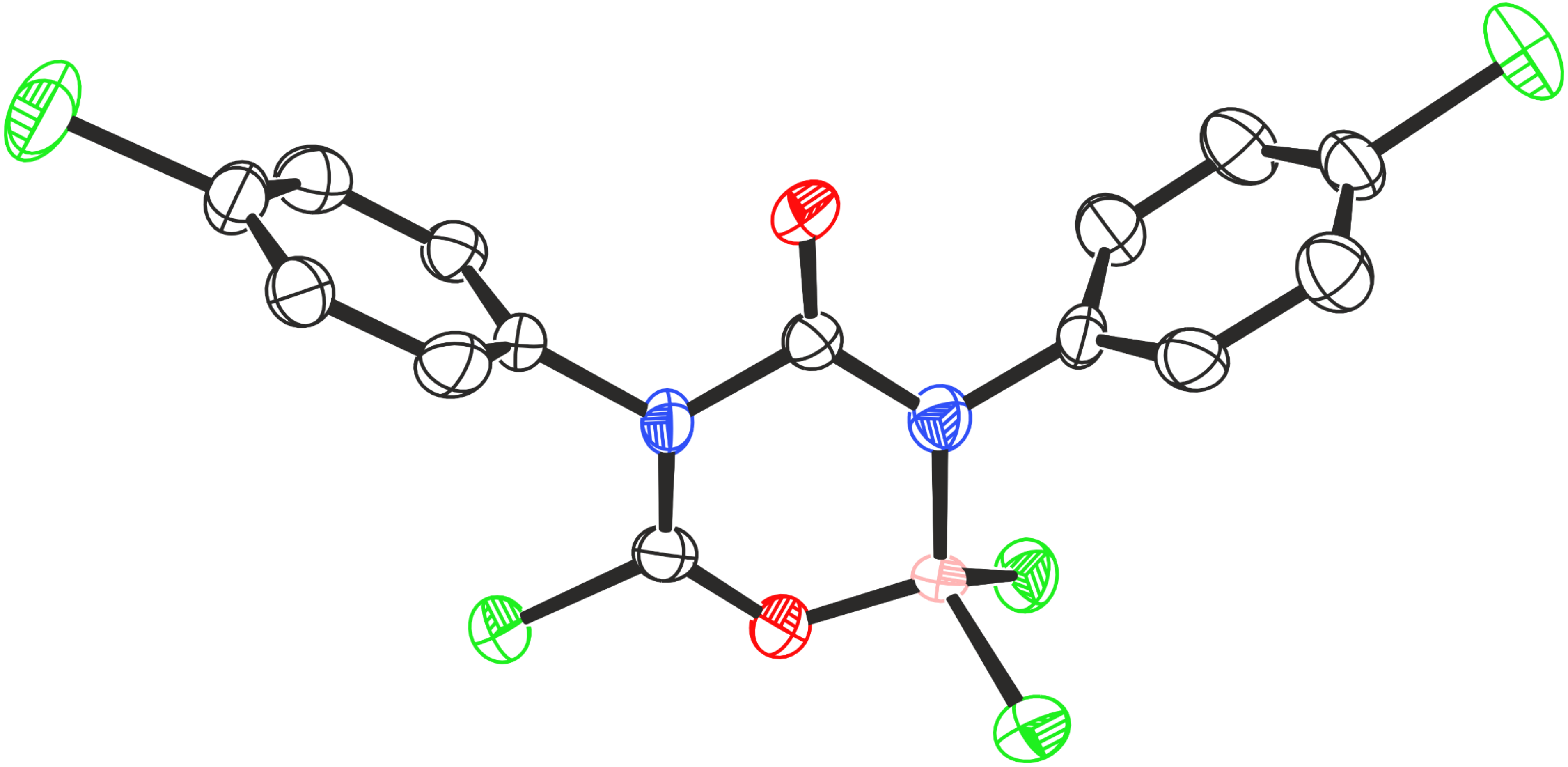

Figure S43: Crystal structure of compound **22**. Thermal ellipsoids drawn at 50% probability.[15] H atoms omitted for clarity. C atoms in black, N in blue, O in red.

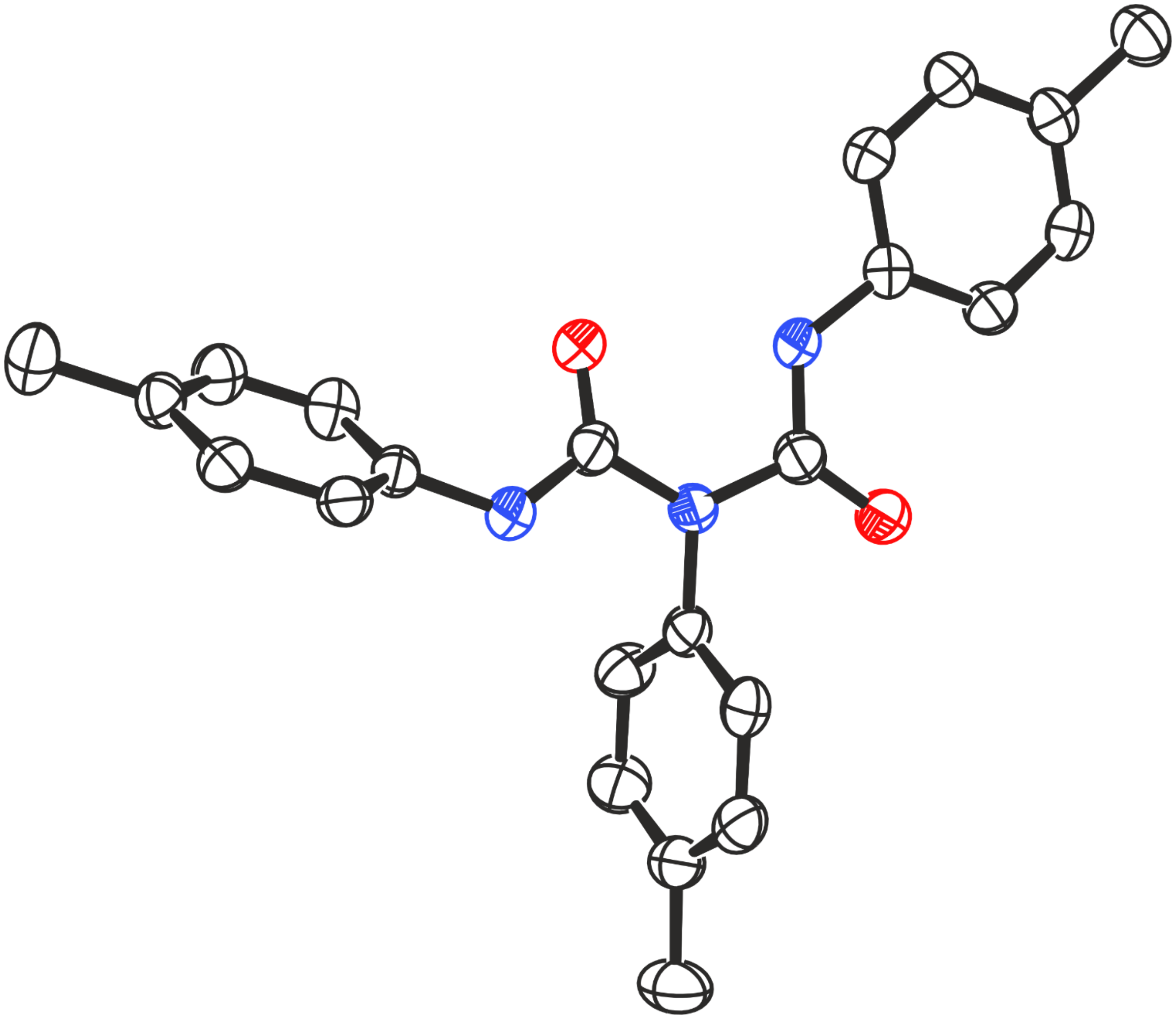

Figure S44: Crystal structure of compound **25**. Thermal ellipsoids drawn at 50% probability.[16] H atoms omitted for clarity. C atoms in black, N in blue, O in red, F in green.

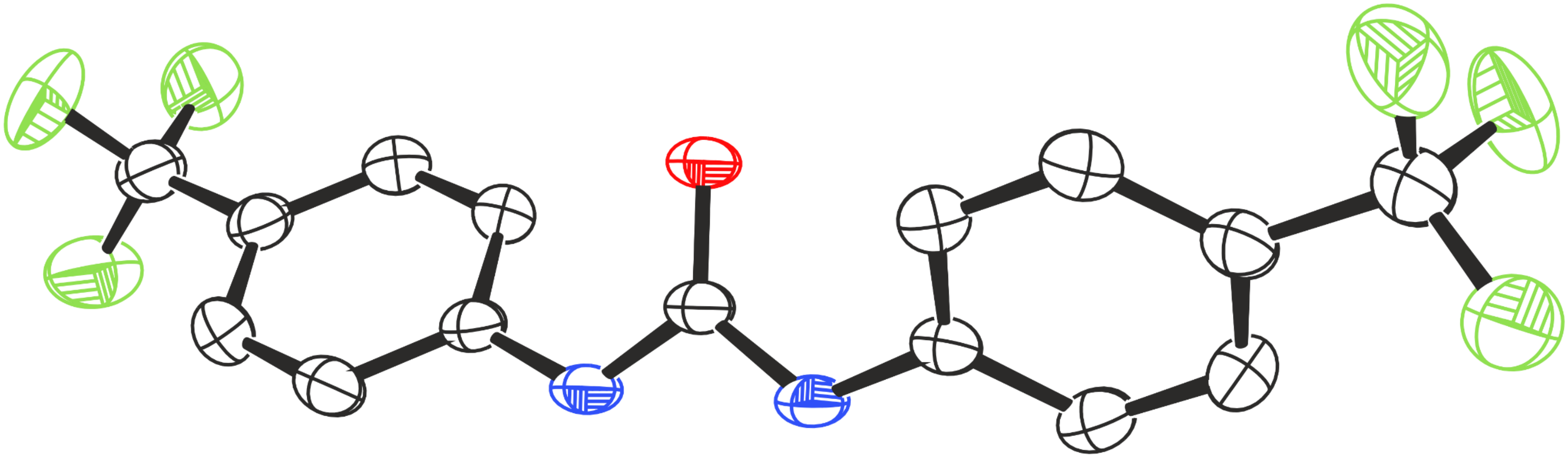

Figure S45: Crystal structure of compound **29**. Thermal ellipsoids drawn at 35% probability. H atoms omitted for clarity. C atoms in black, N in blue, O in red.

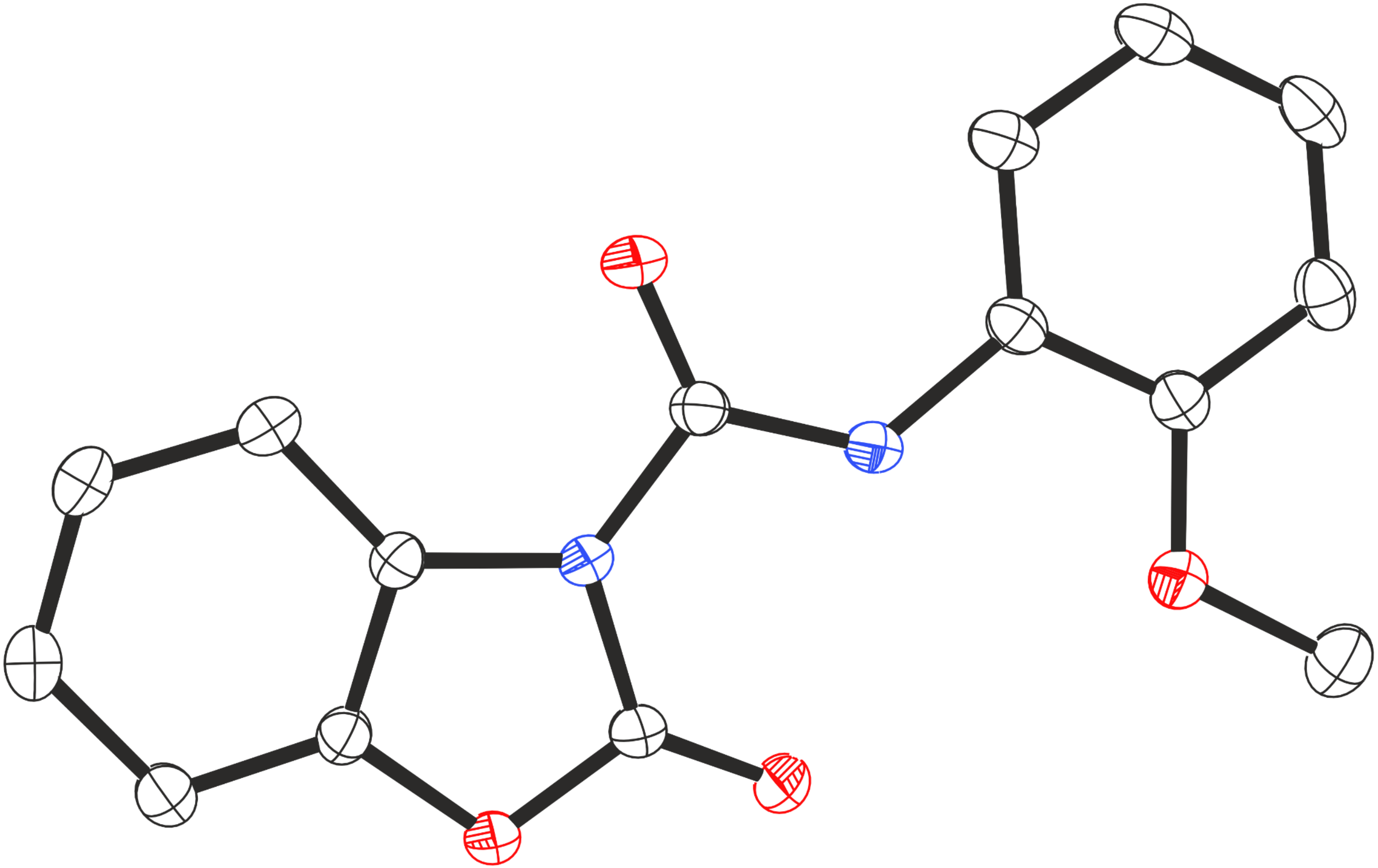

Figure S45: Crystal structure of compound **30a**. Thermal ellipsoids drawn at 35% probability. H atoms omitted for clarity. C atoms in black, N in blue, O in red, B in pink, Cl in green.

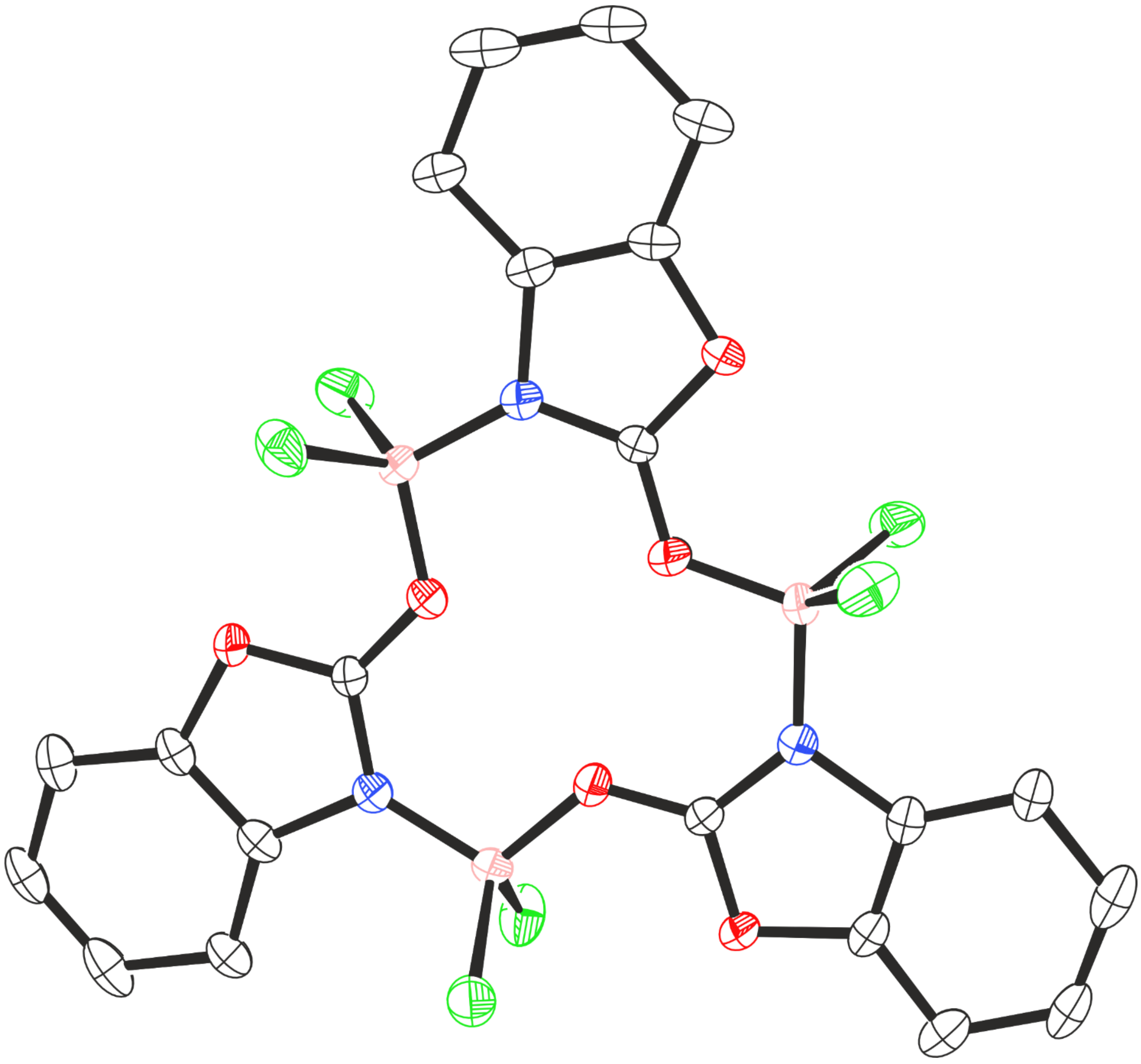

4.3 X-Ray refinement data

Table S1: Crystal data and structure refinement for compound **18**.

**Compound**	**18**
Empirical formula	$C_{20}H_{17}N_3O_2$
M_r	331.36
Crystal system	Monoclinic
Space group	$P2_1/n$
Temperature (K)	180
a, *b*, *c* (Å)	7.6357(5), 12.5253(6), 17.6918(11)
α, β, γ (°)	90, 97.275(6), 90
Volume, V ($Å^3$)	1678.41(17)
Z	4
Density, calc (g cm^{-3})	1.311
Absorption coefficient, μ (mm^{-1})	0.087
Crystal size (mm)	0.456 x 0.171 x 0.097
Radiation type	Mo K\a
Wavelength (Å)	0.71073
θ range (°)	3.454-29.488
Index ranges	-9 ≤ h ≤ 9
	-13 ≤ k ≤17
	-24 ≤ l ≤ 17
Reflections collected	9011
Independent reflections	3966
R(int)	0.0255
Absorption correction	Gaussian
Data / restraints / parameters	3966 / 0 / 234
Goodness of fit, S	1.111
Final R indices [I>2σ(I)]	R_1 = 0.0514
	wR_2 = 0.1250
R indices (all data)	R_1 = 0.0759
	wR_2 = 0.1401
Max residual electron density ($e^-Å^{-3}$)	0.28
Min residual electron density ($e^-Å^{-3}$)	-0.23

Table S2: Crystal data and structure refinement for compound **20**.

**Compound**	**20**
Empirical formula	$C_{20} H_{14} Cl_3 N_3 O_2$
M_r	434.69
Crystal system	Monoclinic
Space group	P 21/n
Temperature (K)	180
a, *b*, *c* (Å)	12.1805(4), 21.0663(8), 15.4545(6)
α, β, γ (°)	90, 95.059(3), 90
Volume, V (Å^3)	3950.2(3)
Z	8
Density, calc (g cm^{-3})	1.462
Absorption coefficient, μ (mm^{-1})	0.485
Crystal size (mm)	0.473, 0.396, 0.131
Radiation type	Mo K\a
Wavelength (Å)	0.71073
θ range (°)	3.352, 29.977
Index ranges	–16≤ h ≤ 15
	-28≤ k ≤29
	-19 ≤ l ≤ 21
Reflections collected	42453
Independent reflections	10219
R(int)	0.0478
Absorption correction	Gaussian
Data / restraints / parameters	10219 / 0 / 509
Goodness of fit, S	1.041
Final R indices [I>2σ(I)]	R_1 = 0.0500
	wR_2 = 0.1117
R indices (all data)	R_1 = 0.0973
	wR_2 = 0.1421
Max residual electron density (e^-Å^{-3})	0.35
Min residual electron density (e^-Å^{-3})	-0.47

Table S3: Crystal data and structure refinement for compound **20a**.

**Compound**	**20a**
Empirical formula	$C_{14}H_8Cl_5N_2O_2$, 1.25($CHCl_3$)
M_r	573.50
Crystal system	Monoclinic
Space group	$P2_1/c$
Temperature (K)	180
a, *b*, *c* (Å)	19.9429(3), 18.1345(5), 25.6229 (5)
α, β, γ (°)	90, 93.877(2), 90
Volume, V (Å^3)	9245.4(3)
Z	16
Density, calc (g cm^{-3})	1.648
Absorption coefficient, μ (mm^{-1})	0.986
Crystal size (mm)	0.61, 0.24, 0.10
Radiation type	Cu K\a
Wavelength (Å)	1.54184
θ range (°)	3.6-72.6
Index ranges	$-14 \leq h \leq 20$
	$-17 \leq k \leq 18$
	$-25 \leq l \leq 26$
Reflections collected	26367
Independent reflections	10525
R(int)	0.0950
Absorption correction	Gaussian
Data / restraints / parameters	10525 / 0 /1045
Goodness of fit, S	1.032
Final R indices [I>2σ(I)]	R_1 = 0.0976
	wR_2 = 0.2399
R indices (all data)	R_1 = 0.1300
	wR_2 = 0.2856
Max residual electron density (e^-Å^{-3})	1.08
Min residual electron density (e^-Å^{-3})	-0.95

Table S4: Crystal data and structure refinement for compound **22**.

**Compound**	**22**
Empirical formula	$C_{23} H_{23} N_3 O_2$
M_r	373.44
Crystal system	Orthorhombic
Space group	P 21 21 21
Temperature (K)	180
a, *b*, *c* (Å)	9.6994(8), 12.3251(8), 16.4781(11)
α, β, γ (°)	90, 90, 90
Volume, V (Å^3)	1969.9(2)
Z	4
Density, calc (g cm^{-3})	1.259
Absorption coefficient, μ (mm^{-1})	0.082
Crystal size (mm)	0.226, 0.120, 0.076
Radiation type	Mo K\a
Wavelength (Å)	0.71073
θ range (°)	3.530-29.799
Index ranges	$–13 \leq h \leq 9$
	$-16 \leq k \leq 17$
	$-22 \leq l \leq 21$
Reflections collected	10726
Independent reflections	4737
R(int)	0.0443
Absorption correction	Gaussian
Data / restraints / parameters	4739 / 0 / 257
Goodness of fit, S	1.066
Final R indices [I>2σ(I)]	R_1 = 0.0554
	wR_2 = 0.1057
R indices (all data)	R_1 = 0.1098
	wR_2 = 0.1253
Max residual electron density (e^-Å^{-3})	0.18
Min residual electron density (e^-Å^{-3})	-0.17

Table S5: Crystal data and structure refinement for compound **25**.

**Compound**	**25**
Empirical formula	C_{15} H_{10} F_6 N_2 O
M_r	348.25
Crystal system	Monoclinic
Space group	C 2/c
Temperature (K)	180
a, *b*, *c* (Å)	14.3767(10, 4.7555(3), 20.5532(18)
α, β, γ (°)	90, 94.332(7), 90
Volume, V (Å^3)	1401.18(18)
Z	4
Density, calc (g cm^{-3})	1.651
Absorption coefficient, μ (mm^{-1})	1.425
Crystal size (mm)	0.284, 0.099, 0.024
Radiation type	Cu K\a
Wavelength (Å)	1.54178
θ range (°)	4.3280, 71.0800
Index ranges	–13≤ h ≤ 17
	-5≤ k ≤5
	-23 ≤ l ≤ 25
Reflections collected	4160
Independent reflections	1375
R(int)	0.0375
Absorption correction	Gaussian
Data / restraints / parameters	1375 / 1 / 114
Goodness of fit, S	1.047
Final R indices [I>2σ(I)]	R_1 = 0.0510
	wR_2 = 0.1371
R indices (all data)	R_1 = 0.0654
	wR_2 = 0.1524
Max residual electron density (e^-Å^{-3})	0.34
Min residual electron density (e^-Å^{-3})	-0.26

Table S6: Crystal data and structure refinement for compound **29.**

**Compound**	**29**
Empirical formula	$C_{15}H_{12}N_2O_4$
M_r	284.27
Crystal system	Monoclinic
Space group	$P2_1/c$
Temperature (K)	180
a, *b*, *c* (Å)	6.9774 (5), 10.8607 (6), 16.8160 (11)
α, β, γ (°)	90, 94.150 (6), 90
Volume, V (Å^3)	1270.97 (14)
Z	4
Density, calc (g cm^{-3})	1.486
Absorption coefficient, μ (mm^{-1})	0.11
Crystal size (mm)	0.74, 0.37, 0.10
Radiation type	Mo K\a
Wavelength (Å)	0.71073
θ range (°)	4.0990, 29.2510
Index ranges	$-9 \leq h \leq 8$
	$-13 \leq k \leq 14$
	$-16 \leq l \leq 21$
Reflections collected	5786
Independent reflections	2815
R(int)	0.0241
Absorption correction	Gaussian
Data / restraints / parameters	2815 / 0 / 191
Goodness of fit, S	1.123
Final R indices [I>2σ(I)]	$R_1 = 0.0587$
	$wR_2 = 0.1467$
R indices (all data)	$R_1 = 0.0746$
	$wR_2 = 0.1574$
Max residual electron density (e^-Å^{-3})	0.81
Min residual electron density (e^-Å^{-3})	-0.31

Table S7. Crystal data and structure refinement for compound **30a.**

**Compound**	**30a**
Empirical formula	$C_{21}H_{12}B_3Cl_6N_3O_6$
M_r	647.47
Crystal system	Hexagonal
Space group	$P6_3/m$
Temperature (K)	180
a, b, c (Å)	18.0280 (9), 6.8007 (4), 1914.2 (2)
α, β, γ (°)	90, 90, 120
Volume, V (Å^3)	1914.2 (2)
Z	2
Density, calc (g cm^{-3})	1.123
Absorption coefficient, μ (mm^{-1})	0.48
Crystal size (mm)	0.48, 0.34, 0.23
Radiation type	Mo K\a
Wavelength (Å)	0.71073
θ range (°)	3.267-27.496
Index ranges	–21≤ h ≤ 18
	-21≤ k ≤22
	-8 ≤ l ≤ 8
Reflections collected	5768
Independent reflections	1582
R(int)	0.0221
Absorption correction	Gaussian
Data / restraints / parameters	1582 / 0 / 76
Goodness of fit, S	1.012
Final R indices [I>2σ(I)]	R_1 = 0.0327
	wR_2 = 0.0795
R indices (all data)	R_1 = 0.0435
	wR_2 = 0.0849
Max residual electron density (e^-Å^{-3})	0.24
Min residual electron density (e^-Å^{-3})	-0.22

5. Computational Data

5.1 Computational details:

Gaussian 16[17] was used to fully optimise all the structures reported in this paper at the M06-2X level of theory.[18] For all the calculations, solvent effects were considered using the SMD solvation model[19] with dichloroethane as the solvent. The 6-31G(d) basis set[20] was used for all atoms. This basis set combination will be referred to as BS1. Frequency calculations were carried out at the same level of theory as those for the structural optimisation. Transition structures were located using the Berny algorithm. Intrinsic reaction coordinate (IRC) calculations were used to confirm the connectivity between transition structures and minima.[21] To further refine the energies obtained from the SMD/M06-2X/6-31G(d) calculations, we carried out single-point energy calculations using the M06-2X functional method for all of the structures with a larger basis set (BS2). BS2 utilises the def2-TZVP basis set[22] on all atoms with D3 empirical dispersion correction.[23] Tight convergence criterion and ultrafine integral grid were also employed to increase the accuracy of the calculations. In this work, the free energy for each species in solution was calculated using the following formula:

$$G = E(BS2) + G(BS1) - E(BS1) + \Delta G^{1atm\rightarrow 1M} \quad (1)$$

where $\Delta G^{1atm\rightarrow 1M}$ = 1.89 kcal/mol is the free-energy change for compression of 1 mol of an ideal gas from 1 atm to the 1 M solution phase standard state.

5.2 Cartesian coordinates and total energies for the calculated structures:

Total potential (E) and Gibbs free energies (G) of all structures optimised at the SMD/M06-2X/BS1 level of theory along with the total potential energies calculated by SMD/M06-2X-D3/BS2//SMD/M06-2X/BS1 and cartesian coordinates for all of the calculated structures.

1

E (SMD/M06-2X/BS1) = -1405.4610358 au

G (SMD/M06-2X/BS1) = -1405.480883 au

E (SMD/M06-2X-D3/BS2//SMD/M06-2X/BS1) = -1405.57478984 au

B	0.00000000	0.00000000	0.00000000
Cl	0.00000000	1.74493100	0.00000000
Cl	-1.51115400	-0.87246500	0.00000000
Cl	1.51115400	-0.87246500	0.00000000

2

E (SMD/M06-2X/BS1) = -399.573059777 au

G (SMD/M06-2X/BS1) = -399.50079 au

E (SMD/M06-2X-D3/BS2//SMD/M06-2X/BS1) = -399.731207695 au

C	2.53077800	-0.07655500	-0.00010600
O	3.64198800	0.30079900	-0.00093300
N	1.44573300	-0.59461800	0.00103100
C	0.08652000	-0.25808400	0.00047800
C	-0.84931800	-1.29255900	0.00008900
C	-0.32905100	1.07647100	0.00056700
C	-2.20716000	-0.98710900	-0.00042500
H	-0.50290700	-2.32082900	0.00017300
C	-1.68863400	1.36790300	0.00002600
H	0.41323900	1.86909200	0.00111700
C	-2.63134600	0.34020900	-0.00051800
H	-2.93503100	-1.79253500	-0.00076700
H	-2.01085400	2.40459200	0.00002900
H	-3.69120700	0.57395000	-0.00097100

3

E (SMD/M06-2X/BS1) = -1805.05429067 au

G (SMD/M06-2X/BS1) = -1804.980448 au

E (SMD/M06-2X-D3/BS2//SMD/M06-2X/BS1) = -1805.32100188 au

C	0.49460200	1.91746800	0.05078300
O	0.74279900	3.03433800	0.07411200
N	0.20359400	0.69659300	0.02581100
C	-1.22998500	0.36982500	0.01588900
C	-1.87150700	0.15907400	1.22959600
C	-1.87632900	0.26515300	-1.20929200
C	-3.22145400	-0.18167700	1.20650200
H	-1.32650000	0.25973600	2.16204400
C	-3.22627500	-0.07515500	-1.21149000
H	-1.33381900	0.44603700	-2.13122800
C	-3.89410400	-0.29953400	-0.00858600
H	-3.74478600	-0.35252900	2.14131900
H	-3.75332400	-0.16312700	-2.15564900
H	-4.94641400	-0.56493800	-0.01821600
B	1.37426100	-0.42513200	-0.01101300
Cl	1.15682200	-1.47621500	1.47470700
Cl	2.96377200	0.51699100	0.02555300
Cl	1.16271800	-1.36908700	-1.56832900

H_2O

E (SMD/M06-2X/BS1) = -76.3809725135 au

G (SMD/M06-2X/BS1) = -76.377395 au

E (SMD/M06-2X-D3/BS2//SMD/M06-2X/BS1) = -76.433747743 au

O	0.00000000	0.00000000	0.11977100
H	0.00000000	0.76126400	-0.47908300
H	0.00000000	-0.76126400	-0.47908300

$(H_2O)_3$

E (SMD/M06-2X/BS1) = -229.167084716 au

G (SMD/M06-2X/BS1) = -229.125720 au

E (SMD/M06-2X-D3/BS2//SMD/M06-2X/BS1) = -229.316665952 au

O	1.08600200	-1.37624900	0.01150000
H	1.26725000	-0.42396100	-0.08737200
H	0.11905800	-1.37672100	0.08961500
O	0.73780100	1.44859900	-0.10630400
H	-0.17318700	1.09509700	-0.07429700
H	0.90399900	1.75962500	0.79687000
O	-1.55289100	-0.16548800	0.01347100
H	-2.20588300	-0.06898200	0.72579100
H	-2.07853500	-0.23994800	-0.79994300

5

E (SMD/M06-2X/BS1) = -1481.88115497 au

G (SMD/M06-2X/BS1) = -1481.877957 au

E (SMD/M06-2X-D3/BS2//SMD/M06-2X/BS1) = -1482.03676961 au

H	0.79726400	0.34953100	2.09977100
B	-0.00017100	-0.01282100	0.11865900
Cl	1.52858400	-0.89282500	-0.35141700
Cl	0.01296900	1.75549800	-0.37493700
Cl	-1.54069600	-0.87120400	-0.35266900
O	-0.00163300	-0.06205100	1.69400000
H	-0.79793200	0.35600000	2.09834100

6

E (SMD/M06-2X/BS1) = -1881.46771394 au

G (SMD/M06-2X/BS1) = -1881.372387 au

E (SMD/M06-2X-D3/BS2//SMD/M06-2X/BS1) = -1881.77907908 au

C	-0.73513900	2.18633700	-0.27914900
O	-0.45826500	3.29765800	-0.09995200
N	-0.92667100	0.99766300	-0.51686400
C	-2.10781700	0.22920200	-0.29063700
C	-2.07237700	-1.12249100	-0.62279000
C	-3.25801800	0.80795800	0.24601800
C	-3.20534400	-1.90349700	-0.41157700

H	-1.17044400	-1.56045600	-1.03903200
C	-4.38201000	0.01588800	0.45175000
C	-4.36015200	-1.33948100	0.12494300
H	-3.17899200	-2.95771500	-0.66782700
H	-5.27753900	0.46340500	0.87099800
H	-5.24024900	-1.95255900	0.28956400
H	0.41471100	0.17175700	-1.05665100
B	2.17644700	-0.36050700	0.02170900
Cl	1.21182400	-1.20087300	1.33186600
Cl	2.50230800	1.41367700	0.42839200
Cl	3.69550500	-1.25423300	-0.47172100
O	1.25652900	-0.38388700	-1.22114200
H	1.70319700	-0.05918900	-2.03571500
H	-3.27103200	1.86428000	0.50043700

7

E (SMD/M06-2X/BS1) = -1881.47983587 au

G (SMD/M06-2X/BS1) = -1881.379916 au

E (SMD/M06-2X-D3/BS2//SMD/M06-2X/BS1) = -1881.78713174 au

C	0.01519100	-1.14331900	-0.58748200
O	0.27275700	-2.30181400	-0.45360400
N	0.75475700	-0.03906400	-0.57863400
C	2.14049800	0.03111600	-0.27335500
C	2.93570600	-1.09790300	-0.07345100
C	2.68686400	1.31301700	-0.18420200
C	4.28652000	-0.92297400	0.22071100
H	2.51749000	-2.09283100	-0.14568900
C	4.03654200	1.46753400	0.10595800
C	4.84327600	0.34945900	0.31107100
H	4.90575800	-1.80058500	0.37785800
H	4.45484700	2.46671700	0.17380400
H	5.89728400	0.47012000	0.53927600
H	0.29569800	0.84527900	-0.78689900
B	-2.25867800	0.17669000	0.09113800
Cl	-1.66999400	-0.06392800	1.79953700
Cl	-3.96313800	-0.40429700	-0.18938100
Cl	-1.95870200	1.86586500	-0.54545600
O	-1.38071900	-0.79547000	-0.84783400
H	-1.85490200	-1.64277300	-1.02906200
H	2.05120300	2.18087700	-0.33845700

TS_{6-7}

E (SMD/M06-2X/BS1) = -1881.45049641 au

G (SMD/M06-2X/BS1) = -1881.356099 au

E (SMD/M06-2X-D3/BS2//SMD/M06-2X/BS1) = -1881.76132927 au

C	-0.54194800	1.53064100	-0.13932200
O	-0.07367400	2.53895100	0.11084600
N	-1.07497900	0.42618600	-0.44408700
C	-2.45949800	0.05787400	-0.24647900
C	-2.73339900	-1.30047600	-0.15605800
C	-3.44567600	1.03276800	-0.16689200
C	-4.05625500	-1.69214500	0.02647100
H	-1.93042100	-2.02813800	-0.22283500
C	-4.76000600	0.61927900	0.02759000
C	-5.06632000	-0.73707300	0.12247800
H	-4.29185200	-2.74870300	0.09778900
H	-5.54532100	1.36464300	0.09579300
H	-6.09494200	-1.04983600	0.26839200
H	-0.34268800	-0.24376600	-0.79497000
B	2.38412800	-0.25065900	-0.11143000
Cl	1.51021900	-0.20204400	1.55491500
Cl	3.61787500	1.16589500	-0.21693700
Cl	3.30452900	-1.87737400	-0.30146100
O	1.36161100	-0.10505900	-1.10357700
H	1.73378200	-0.11816300	-2.00179800
H	-3.20380500	2.08751100	-0.26228900

TS_{7-8}

E (SMD/M06-2X/BS1) = -1881.46441207 au

G (SMD/M06-2X/BS1) = -1881.367444 au

E (SMD/M06-2X-D3/BS2//SMD/M06-2X/BS1) = -1881.77672950 au

C	-0.29470900	-0.64080500	-0.37742500
O	-0.00919100	0.53421100	-0.45796800
N	-1.55092600	-1.15258200	-0.21521200
C	-2.75782300	-0.43084000	-0.10580000
C	-2.83904000	0.96539600	-0.14263300
C	-3.92585900	-1.18816500	0.04957600
C	-4.08538100	1.57797200	-0.02327400
H	-1.94448500	1.55921800	-0.26238600
C	-5.15941100	-0.56098400	0.16724100
C	-5.24853100	0.82978900	0.13159400
H	-4.13767100	2.66238900	-0.05286800
H	-6.05358400	-1.16515600	0.28730300

H	-6.21139700	1.32186400	0.22374800
H	-1.65665800	-2.16032200	-0.16934700
B	3.07749000	0.26587500	0.17929800
Cl	3.04156900	1.72248000	-0.78118200
Cl	3.90732600	-1.14731300	-0.43383100
Cl	2.42567400	0.25755900	1.79913800
O	0.69127600	-1.55938600	-0.44928800
H	0.37182000	-2.47551700	-0.36198500
H	-3.85881500	-2.27290300	0.07779400

8

E (SMD/M06-2X/BS1) = -1881.50992670 au

G (SMD/M06-2X/BS1) = -1881.409108 au

E (SMD/M06-2X-D3/BS2//SMD/M06-2X/BS1) = -1881.81588467 au

C	0.06073800	-0.18734100	-0.05075000
O	0.21498200	0.93554900	-0.69207600
N	1.13500200	-0.79767700	0.40018500
C	2.49574700	-0.39557300	0.21467800
C	2.88675100	0.92833700	0.39940400
C	3.42048600	-1.38624900	-0.10734200
C	4.22754800	1.26188200	0.22964400
H	2.16093100	1.68249500	0.68190300
C	4.75921600	-1.04041600	-0.26042300
C	5.16345900	0.28355000	-0.09934400
H	4.53984100	2.29189600	0.36825500
H	5.48405700	-1.80810200	-0.51092000
H	6.20772600	0.55157200	-0.22396200
H	0.96222800	-1.68324500	0.87211900
B	-2.41622800	-0.09721200	0.04974800
Cl	-2.66607800	0.43613400	-1.73355400
Cl	-2.44733700	1.39031000	1.15708600
Cl	-3.64322300	-1.36118700	0.53867400
O	-1.07763900	-0.74704500	0.16495100
H	3.08873000	-2.41207500	-0.23782700
H	-0.62694000	1.24472000	-1.09530300

TS^{i}_{3}

E (SMD/M06-2X/BS1) = -2204.61660893 au

G (SMD/M06-2X/BS1) = -2204.447640 au

E (SMD/M06-2X-D3/BS2//SMD/M06-2X/BS1) = -2205.03688446 au

C	-2.27427900	-1.47657500	1.39280200
O	-2.77004400	-2.43832300	1.78952000

N	-1.67402800	-0.46797900	1.00029100
C	-2.29140000	0.67999600	0.38765300
C	-1.72658300	1.93180800	0.60598100
C	-3.40849900	0.51347500	-0.42742500
C	-2.29976000	3.04155300	-0.01191300
H	-0.86598900	2.05543800	1.25601300
C	-3.97631900	1.63308700	-1.02441500
H	-3.81369200	-0.47942200	-0.60321500
C	-3.42198900	2.89668400	-0.82265900
H	-1.86450000	4.02195900	0.15197300
H	-4.84810900	1.51219000	-1.65909000
H	-3.86395100	3.76566900	-1.29901400
C	0.16666800	-0.25064800	1.47643500
O	0.18987900	0.02826300	2.60826800
N	0.83799300	-0.34772500	0.37059200
C	1.96100000	0.58908700	0.33903500
C	3.09275700	0.33590700	1.10467700
C	1.84951500	1.71231600	-0.47147700
C	4.14964700	1.23993500	1.04955900
H	3.14183500	-0.55614500	1.72201700
C	2.91947900	2.60323600	-0.52463500
H	0.94440900	1.87664500	-1.04877000
C	4.06417700	2.36849100	0.23469800
H	5.04202000	1.05763600	1.63951200
H	2.85346100	3.48268900	-1.15696400
H	4.89317500	3.06797900	0.19280400
B	0.62783800	-1.43036800	-0.76464600
Cl	2.27534800	-1.77676300	-1.52079000
Cl	-0.02403900	-2.98796000	0.03564500
Cl	-0.55076500	-0.79836600	-2.04429100

TS^{ii}_{3}

E (SMD/M06-2X/BS1) = -2034.23690311 au

G (SMD/M06-2X/BS1) = -2034.096808 au

E (SMD/M06-2X-D3/BS2//SMD/M06-2X/BS1) = -2034.64320622 au

C	0.40568000	-0.02055000	1.76836200
O	0.32650600	0.15380200	2.89939600
N	0.11782600	-0.38370900	0.58049300
C	-1.33184100	-0.35075700	0.34782500
C	-1.85720700	0.64487200	-0.46492700
C	-2.12162300	-1.33460200	0.92891800
C	-3.22717400	0.63883500	-0.71270400

H	-1.20921000	1.40624500	-0.88422800
C	-3.49191700	-1.31879900	0.68233400
H	-1.66596700	-2.09829300	1.55248000
C	-4.04168600	-0.33717700	-0.14000700
H	-3.65874600	1.40378600	-1.35015500
H	-4.12532400	-2.07880000	1.12779300
H	-5.10942800	-0.33132200	-0.33474700
B	1.14404100	-0.88212700	-0.51968900
Cl	0.26146700	-2.08688100	-1.59269800
Cl	1.70381900	0.58618200	-1.53692700
Cl	2.57801400	-1.68274200	0.32660500
O	2.28054800	1.15834000	1.53275200
H	2.83501400	1.02760600	0.74699900
H	1.70716000	1.93510400	1.32896400
O	0.12759600	2.64673000	1.07226300
H	-0.12411700	3.37151700	1.66618100
H	0.05437000	3.01003900	0.16062800
O	0.01231000	3.49085500	-1.53881900
H	-0.85720700	3.66516700	-1.93547900
H	0.39130400	2.77531800	-2.07564400

4

E (SMD/M06-2X/BS1) = -1805.04682784 au

G (SMD/M06-2X/BS1) = -1804.975172 au

E (SMD/M06-2X-D3/BS2//SMD/M06-2X/BS1) = -1805.31330854 au

C	-0.10290600	0.04601000	-0.82405100
O	-1.29933200	0.06631700	-1.11445500
N	1.02774400	0.02887600	-0.59339700
C	2.39382400	0.01385900	-0.31343300
C	3.06539000	1.22970800	-0.19609200
C	3.03261400	-1.21571100	-0.16244100
C	4.42701900	1.20335400	0.08155700
H	2.52476000	2.16162200	-0.32121600
C	4.39432900	-1.21792700	0.11620700
H	2.46731000	-2.13595300	-0.26164000
C	5.08863000	-0.01444900	0.23715200
H	4.97045300	2.13732300	0.17699900
H	4.91247400	-2.16299700	0.23913500
H	6.15177000	-0.02576500	0.45473500
B	-2.39969800	0.00104800	0.02054600
Cl	-2.14136800	-1.59373100	0.89348800
Cl	-3.97395800	0.08941700	-0.88519300

Cl	-2.09768700	1.44659800	1.11201500

TS^{i}_{4}

E (SMD/M06-2X/BS1) = -2204.60948465 au

G (SMD/M06-2X/BS1) = -2204.441358 au

E (SMD/M06-2X-D3/BS2//SMD/M06-2X/BS1) = -2205.02810226 au

C	0.36373300	2.97004900	0.07996700
O	1.06291200	3.88110000	0.05542200
N	-0.32049300	1.93012500	0.10474000
C	-1.75997100	1.88238500	0.14880500
C	-2.49557700	2.29916500	-0.95576200
C	-2.35271100	1.31721800	1.27228500
C	-3.88001000	2.15392600	-0.92063000
H	-1.98975900	2.71692600	-1.82076400
C	-3.73691200	1.16920800	1.28570600
H	-1.73927700	0.99662200	2.10848400
C	-4.49704700	1.58578500	0.19369200
H	-4.47317500	2.47685500	-1.76981900
H	-4.21870700	0.72552900	2.15066200
H	-5.57558400	1.46604200	0.21022900
C	0.53588500	0.40581900	0.00363100
O	1.75488800	0.83046000	-0.00902500
N	-0.12406800	-0.57820800	-0.03821200
C	-1.02410100	-1.62951300	-0.08814700
C	-0.61972200	-2.86764700	0.41949000
C	-2.29416800	-1.45833800	-0.64717200
C	-1.50604800	-3.93803800	0.37980200
H	0.37803000	-2.96963000	0.83407000
C	-3.16267000	-2.54311200	-0.69140100
H	-2.58348100	-0.49042300	-1.04445600
C	-2.77519800	-3.77921600	-0.17513200
H	-1.19930700	-4.90036500	0.77674800
H	-4.14881400	-2.41759400	-1.12715200
H	-3.46143600	-4.61955600	-0.20829800
B	2.89774300	-0.13977200	-0.04297800
Cl	2.74999100	-1.22051800	-1.54369200
Cl	2.86264700	-1.16084100	1.51059100
Cl	4.40615200	0.91973600	-0.11763300

TS^{ii}_{4}

E (SMD/M06-2X/BS1) = -2034.22456591 au

G (SMD/M06-2X/BS1) = -2034.087336 au

E (SMD/M06-2X-D3/BS2//SMD/M06-2X/BS1) = -2034.63202265 au

C	-0.34003600	-0.59930400	0.86518600
O	-1.53160800	-0.32123600	1.04544600
N	0.59680400	-1.15970400	0.41598600
C	1.97271200	-1.06616300	0.17203000
C	2.53346900	0.18975100	-0.06895100
C	2.73622500	-2.23002300	0.15315200
C	3.89759600	0.27177600	-0.32180800
H	1.90033900	1.07216000	-0.06517300
C	4.10125400	-2.12707300	-0.09599600
H	2.26115800	-3.18815200	0.33459200
C	4.68150500	-0.88196100	-0.33301600
H	4.35010200	1.23973000	-0.51340000
H	4.71125200	-3.02424600	-0.10853400
H	5.74600600	-0.81005800	-0.53149400
B	-2.50746900	-0.32618600	-0.16381600
Cl	-4.05788800	0.37494100	0.48576500
Cl	-1.73846700	0.73920900	-1.47640500
Cl	-2.67207700	-2.06418500	-0.72906100
O	0.36415300	0.83858300	2.28655500
H	1.31804900	0.75142100	2.45239100
H	0.25923300	1.66653700	1.74627100
O	0.00625800	2.83408400	0.56011000
H	-0.68559800	2.58528000	-0.07375500
H	0.78252100	3.09685400	0.01798000
O	2.24095000	3.47717600	-0.91794400
H	2.46843100	2.93829000	-1.69309700
H	3.07719100	3.60076600	-0.44059300

TS_{6-13}

E (SMD/M06-2X/BS1) = -2168.95756801 au

G (SMD/M06-2X/BS1) = -2168.757126 au

E (SMD/M06-2X-D3/BS2//SMD/M06-2X/BS1) = -2169.38181658 au

N	-1.72403900	-0.25798400	-1.13557200
C	-2.92976700	-0.63439400	-0.50790300
C	-3.58075900	-1.81648300	-0.87670900
C	-3.44593000	0.13572600	0.54056000
C	-4.73533500	-2.21504200	-0.20882600
H	-3.17720100	-2.41700100	-1.68817700
C	-4.59924300	-0.27167200	1.20268700
H	-2.93733300	1.05323800	0.82691600
C	-5.25174700	-1.44792400	0.83393300

H	-5.23362100	-3.13256800	-0.50836000
H	-4.99143800	0.33815000	2.01142800
H	-6.15241300	-1.76128800	1.35218700
C	0.01246000	-1.85841600	-0.20623200
O	-0.76732800	-2.67480100	0.03286900
N	0.98462800	-1.10162100	-0.40777700
C	2.33127700	-1.53964800	-0.13617600
C	3.25863500	-0.56391800	0.21844500
C	2.69398300	-2.87906400	-0.25123800
C	4.57428500	-0.94458900	0.46746900
H	2.95562200	0.47485200	0.31445300
C	4.01137800	-3.24485500	0.00822100
H	1.96137700	-3.62477900	-0.54796500
C	4.95305800	-2.28149400	0.36554200
H	5.30124700	-0.18910000	0.74756500
H	4.30012500	-4.28755400	-0.07691300
H	5.97967000	-2.57225600	0.56312800
B	0.52995100	2.35285300	-0.10004500
Cl	0.12575400	1.51843600	1.49881100
Cl	2.06778500	3.38106100	0.02180500
Cl	-0.90300900	3.37482500	-0.70026200
H	-1.69120400	-0.54984800	-2.11093400
H	-1.57167100	0.74812200	-1.09437200
O	0.75837000	1.26498900	-1.09622000
H	0.82214000	0.16022600	-0.77600300
H	1.33970400	1.49843100	-1.84713200

TS_{8-9}

E (SMD/M06-2X/BS1) = -1881.46924014 au

G (SMD/M06-2X/BS1) = -1881.370470 au

E (SMD/M06-2X-D3/BS2//SMD/M06-2X/BS1) = -1881.78216452 au

C	-0.23164000	1.77473000	-0.76508900
O	0.29338200	2.59410000	0.15704300
N	0.54006200	0.68171800	-1.03114300
C	1.76820900	0.25132200	-0.48561100
C	2.22616400	0.61812900	0.78410800
C	2.51881300	-0.64305900	-1.25851800
C	3.43614800	0.10759400	1.24895800
H	1.64907400	1.29070300	1.40398400
C	3.71843400	-1.15242900	-0.77667900
C	4.18915800	-0.77525700	0.47972100
H	3.78418900	0.40277900	2.23439400

H	4.28732100	-1.84418500	-1.39049600
H	5.12838600	-1.16813200	0.85553400
H	0.20781600	0.16240700	-1.83647300
B	-2.10685200	-0.76544800	0.37595500
Cl	-1.51295500	0.15371400	1.73779800
Cl	-1.20342800	-2.14762300	-0.19826900
Cl	-3.62508700	-0.34827700	-0.36938900
O	-1.29983400	1.97606600	-1.31093400
H	2.15317600	-0.93356200	-2.24014600
H	-0.35125400	3.31486800	0.28884200

$TS_{10\text{-}11}$

E (SMD/M06-2X/BS1) = -2110.67339167 au

G (SMD/M06-2X/BS1) = -2110.510362 au

E (SMD/M06-2X-D3/BS2//SMD/M06-2X/BS1) = -2111.11874728 au

C	0.14420100	0.43349600	2.19169500
O	-0.72871100	-0.04161800	2.82355100
N	-0.32827000	0.11869600	0.25083400
C	-1.59569200	0.69773000	-0.10612400
C	-1.64466700	1.64309300	-1.13384800
C	-2.76350800	0.36459400	0.58507600
C	-2.85160000	2.24623300	-1.47582100
H	-0.73156600	1.90118200	-1.66533100
C	-3.96717100	0.97008800	0.23296400
H	-2.72384300	-0.35190000	1.39553800
C	-4.01954900	1.90870700	-0.79576500
H	-2.87614200	2.97769200	-2.27798500
H	-4.87075700	0.70633700	0.77446300
H	-4.96238900	2.37604900	-1.06243400
B	-0.02686800	-1.33149600	-0.14986400
Cl	0.24485700	-1.40643900	-2.03942800
Cl	1.60419000	-1.82167800	0.66955600
Cl	-1.33749900	-2.52742900	0.32387000
O	1.18012900	1.03170000	2.11405100
H	2.28877800	0.94191200	0.76672000
H	0.38902100	0.69749300	-0.19065100
H	3.85204600	-1.32151000	-1.22806600
O	3.38698900	-0.58380800	-1.66395400
H	2.55245300	-0.97482000	-1.99552700
H	2.99935600	0.39740000	-0.70817200
O	2.63334600	1.20797500	-0.12502100
H	3.31803600	1.99236900	-0.04234500

O	4.28631800	3.09191600	0.00857900
H	3.85686900	3.96068100	-0.09214800
H	4.76739500	3.13504200	0.85472500

CO_2
E (SMD/M06-2X/BS1) = -188.507988505 au
G (SMD/M06-2X/BS1) = -188.516875 au
E (SMD/M06-2X-D3/BS2//SMD/M06-2X/BS1) = -188.595634284 au

C	0.00000000	0.00000000	0.00000000
O	0.00000000	0.00000000	1.16265200
O	0.00000000	0.00000000	-1.16265200

$PhNH_2$
E (SMD/M06-2X/BS1) = -1769.41178625 au
G (SMD/M06-2X/BS1) = -1769.299260 au
E (SMD/M06-2X-D3/BS2//SMD/M06-2X/BS1) = -1769.67729149 au

N	2.33041400	-0.00024000	-0.08194900
C	0.93772100	0.00010400	-0.01019600
C	0.22175800	1.20662800	-0.00509400
C	0.22165700	-1.20661500	-0.00521900
C	-1.16909900	1.20075400	0.00361300
H	0.76802000	2.14664900	-0.00869500
C	-1.16900900	-1.20083300	0.00352200
H	0.76820900	-2.14646000	-0.00909800
C	-1.87806600	0.00004000	0.00757500
H	-1.70270600	2.14719600	0.00966700
H	-1.70275600	-2.14719600	0.00956200
H	-2.96327300	-0.00003600	0.01546400
H	2.76507300	0.83560600	0.29391400
H	2.76476600	-0.83455400	0.29762300

12
E (SMD/M06-2X/BS1) = -1769.41178625 au
G (SMD/M06-2X/BS1) = -1769.299260 au
E (SMD/M06-2X-D3/BS2//SMD/M06-2X/BS1) = -1769.67729149 au

N	-1.15584200	-1.81867200	0.03909700
C	-2.19246600	-0.78003100	0.03855800
C	-3.48781200	-1.11073200	0.40765800
C	-1.82897800	0.50784600	-0.33301100
C	-4.45680100	-0.10973800	0.40175600
H	-3.73481300	-2.12897100	0.69441800
C	-2.80863700	1.49678300	-0.33544500

H	-0.80326000	0.73445100	-0.61098800
C	-4.11831800	1.18984400	0.03096100
H	-5.47534600	-0.34958700	0.68866700
H	-2.54256100	2.50926200	-0.62102000
H	-4.87640900	1.96656000	0.02926200
H	-1.46043600	-2.65346100	0.55365500
H	-0.91882600	-2.11797900	-0.91571400
H	-0.27451300	-1.44874600	0.48373100
B	1.98224200	0.02230300	0.16656100
Cl	1.68960600	-0.78295100	-1.51066600
Cl	1.77714400	1.89515400	0.00211000
Cl	3.73845900	-0.33186500	0.74368500
O	1.01237100	-0.52709500	1.05077300
H	1.03639400	-0.10516000	1.92526400

13

E (SMD/M06-2X/BS1) = -2169.00882437 au

G (SMD/M06-2X/BS1) = -2168.796152 au

E (SMD/M06-2X-D3/BS2//SMD/M06-2X/BS1) = -2169.42632854 au

N	1.05758000	1.00778200	-1.14896600
C	2.25979500	1.45906400	-0.43256200
C	2.89341200	2.61956800	-0.85430200
C	2.69745800	0.71940000	0.65695600
C	4.02088200	3.04616100	-0.15806500
H	2.51523600	3.17488300	-1.70714900
C	3.82713600	1.15934200	1.34186300
H	2.17202400	-0.18239400	0.96118000
C	4.48611200	2.31808300	0.93589100
H	4.53445200	3.94831600	-0.47343000
H	4.18945600	0.59123200	2.19210000
H	5.36683500	2.65474400	1.47331900
C	-0.21918100	1.70877400	-0.67146600
O	-0.16627700	2.89326400	-0.47823900
N	-1.22546400	0.84029700	-0.56990000
C	-2.55505400	1.13584500	-0.17489300
C	-3.41317500	0.04027900	-0.04103400
C	-3.01598800	2.43161200	0.06602200
C	-4.73509400	0.24118100	0.33499000
H	-3.03840500	-0.96330400	-0.22835800
C	-4.34529100	2.61348600	0.44288700
H	-2.35439900	3.28107900	-0.03551800
C	-5.20816800	1.52983600	0.57894600

H	-5.39480600	-0.61449800	0.43830700
H	-4.70354400	3.62103300	0.63039100
H	-6.24084100	1.68646500	0.87351900
B	0.32681500	-2.58722400	-0.14622700
Cl	-0.13675400	-1.77971600	1.47825200
Cl	-0.73082800	-4.10682200	-0.44561100
Cl	2.14055800	-3.09435900	-0.10419700
H	1.15320000	1.23688200	-2.15055500
H	0.95327800	-0.03477600	-1.10187600
O	0.09914700	-1.60608400	-1.17531300
H	-1.02588600	-0.14728500	-0.75626600
H	0.15389600	-2.01078300	-2.05897200

14

E (SMD/M06-2X/BS1) = -687.101576398 au

G (SMD/M06-2X/BS1) = -686.914276 au

E (SMD/M06-2X-D3/BS2//SMD/M06-2X/BS1) = -687.369698048 au

N	-1.14480800	-0.74295500	-0.15264400
C	-2.47633200	-0.30509300	-0.06253800
C	-2.86317800	1.04106800	-0.03813300
C	-3.46103100	-1.30246100	-0.00715600
C	-4.21685200	1.36200400	0.04744500
H	-2.11580000	1.81951000	-0.08429900
C	-4.80539600	-0.96451300	0.07314900
H	-3.15964100	-2.34705300	-0.02451900
C	-5.19535800	0.37381400	0.10366300
H	-4.50292100	2.40969100	0.06746000
H	-5.54989100	-1.75401200	0.11557400
H	-6.24538500	0.64018500	0.16931900
C	-0.00025100	0.01699400	-0.04856600
O	0.00055000	1.23470900	0.06386200
N	1.14368900	-0.74987700	-0.09080700
C	2.47555100	-0.30857200	-0.03025100
C	3.46066800	-1.30236400	0.06661000
C	2.86292600	1.03681400	-0.07905700
C	4.80583600	-0.96159100	0.11545600
H	3.15921800	-2.34640200	0.10422700
C	4.21744300	1.36078500	-0.02582500
H	2.11525200	1.81263600	-0.15554600
C	5.19642900	0.37612200	0.07113300
H	5.55062000	-1.74840800	0.19040900
H	4.50388200	2.40788500	-0.06383700

H	6.24715600	0.64476100	0.11099600
H	-1.03657200	-1.74739900	-0.23668900
H	1.03477600	-1.75729800	-0.11542500

15

E (SMD/M06-2X/BS1) = -2169.01750952 au

G (SMD/M06-2X/BS1) = -2168.807410 au

E (SMD/M06-2X-D3/BS2//SMD/M06-2X/BS1) = -2169.43914751 au

N	2.03182900	-1.61794300	-0.52604700
C	3.20493200	-0.89290200	-0.16939000
C	3.41908000	-0.38341500	1.11164000
C	4.17904300	-0.75604700	-1.16031600
C	4.60799000	0.29113800	1.37916100
H	2.67793200	-0.51345400	1.88884800
C	5.36638800	-0.09259100	-0.87376800
H	3.99781200	-1.16488400	-2.15076100
C	5.58248800	0.44017000	0.39577000
H	4.77184700	0.69330100	2.37399500
H	6.11934900	0.01254200	-1.64845000
H	6.50649200	0.96417800	0.61819200
C	0.78273600	-1.40402400	-0.11019800
O	0.58627200	-0.60794400	0.88375400
N	-0.21896100	-2.05554000	-0.71371600
C	-1.56765000	-2.05370800	-0.25076900
C	-2.58351300	-1.68137100	-1.12967400
C	-1.85011900	-2.40418100	1.06933900
C	-3.89902000	-1.64830100	-0.67335100
H	-2.33641800	-1.39980000	-2.14824300
C	-3.16653200	-2.34730600	1.51985600
H	-1.04679200	-2.70407500	1.73521500
C	-4.19112500	-1.96979800	0.65243600
H	-4.69273600	-1.35269200	-1.35169100
H	-3.38981300	-2.60463500	2.55011100
H	-5.21537800	-1.92657300	1.00818000
H	2.14791200	-2.28949800	-1.28034100
H	-0.03034900	-2.48894900	-1.61364600
B	-1.35181000	1.86762700	0.03418800
Cl	-3.00576100	2.69572300	-0.25031000
Cl	-0.13538000	3.02922600	0.82821300
Cl	-0.65891600	1.29982900	-1.63980900
H	-0.35230000	-0.13248000	0.90464400
O	-1.51909800	0.73654200	0.91719500

H	-2.33320900	0.24724400	0.70174000

TS$_{6-16}$

E (SMD/M06-2X/BS1) = -2568.57339882 au
G (SMD/M06-2X/BS1) = -2568.268817 au
E (SMD/M06-2X-D3/BS2//SMD/M06-2X/BS1) = -2569.14898500 au

C	0.88239200	0.83132000	-1.38002500
O	0.23395600	1.40423600	-2.13392400
N	1.80367900	0.19680700	-0.78504000
C	3.01942300	-0.19782100	-1.47051500
C	3.65536700	-1.34149900	-1.00200100
C	3.51208200	0.55191500	-2.53115500
C	4.83263100	-1.74198300	-1.62717800
H	3.23748500	-1.90436000	-0.17268400
C	4.68443600	0.12728700	-3.14937500
H	3.00421200	1.45144000	-2.86754600
C	5.34518400	-1.01397900	-2.69918000
H	5.34394500	-2.63133300	-1.27392100
H	5.08281400	0.69931800	-3.98066500
H	6.26114300	-1.33557200	-3.18383800
B	0.42968500	-2.17972000	1.47575900
Cl	-0.13495800	-2.43812900	-0.29071100
Cl	1.23795600	-3.70054800	2.16493300
Cl	-1.10255800	-1.74837500	2.52078000
O	1.32826200	-1.05758900	1.46179500
H	1.61348400	-0.22240100	0.18374500
H	1.83103900	-0.94833600	2.28811100
N	-2.43902600	0.22201900	-0.05018900
C	-3.68705900	0.01533400	-0.67140200
C	-4.36973200	0.99487800	-1.40105500
C	-4.24487100	-1.26243900	-0.53353200
C	-5.59803500	0.67908300	-1.97952900
H	-3.94851300	1.98357800	-1.51294300
C	-5.46919900	-1.55918500	-1.11757100
H	-3.71122700	-2.02092100	0.03440500
C	-6.15632100	-0.58865400	-1.84573900
H	-6.12116500	1.44561500	-2.54375200
H	-5.88557400	-2.55509000	-1.00057100
H	-7.11359600	-0.81926000	-2.30213600
C	-1.71230100	1.37335600	-0.00396700
O	-2.02962200	2.43741900	-0.49974600
N	-0.45150900	1.17998700	0.61963600

C	0.26559500	2.28562500	1.17794200
C	0.82321200	2.13261600	2.44970500
C	0.48705800	3.45954700	0.45185800
C	1.59474100	3.15308000	2.99732000
H	0.64206900	1.22138700	3.01405400
C	1.25117800	4.47701200	1.01783000
H	0.05503000	3.58208600	-0.53318400
C	1.80974200	4.33096500	2.28554700
H	2.02049900	3.02552600	3.98774100
H	1.41307500	5.39061900	0.45402100
H	2.40746500	5.12842600	2.71521500
H	-2.06059800	-0.59330200	0.42450200
H	-0.43340700	0.37809400	1.25190800

16

E (SMD/M06-2X/BS1) = -2568.60287916 au

G (SMD/M06-2X/BS1) = -2568.291163 au

E (SMD/M06-2X-D3/BS2//SMD/M06-2X/BS1) = -2569.17347760 au

C	0.87360600	0.93478600	-0.92690600
O	0.56175500	1.40197100	-1.98980500
N	1.95268600	0.25969000	-0.53619000
C	3.02991300	-0.13478200	-1.37236300
C	3.82918900	-1.17894300	-0.90137200
C	3.31086900	0.48725700	-2.58922900
C	4.91587700	-1.60526300	-1.65474100
H	3.59138600	-1.65317300	0.04805600
C	4.40216700	0.04397300	-3.33326300
H	2.69368000	1.29970600	-2.95033300
C	5.20574000	-0.99684500	-2.87514000
H	5.53354300	-2.41808700	-1.28618500
H	4.62292300	0.52563200	-4.28071600
H	6.05314400	-1.33210200	-3.46439900
B	0.26124700	-2.19010700	1.72456100
Cl	0.08351600	-2.50226200	-0.12993600
Cl	1.27962800	-3.53596000	2.51035500
Cl	-1.45618100	-2.14960800	2.49696800
O	0.89843000	-0.90600100	1.84175400
H	1.93734200	-0.15685300	0.39936500
H	1.10707800	-0.70373200	2.77151700
N	-2.06271800	0.14855800	-0.54454800
C	-3.36842900	-0.06743200	-1.06457500
C	-4.12303000	0.94315700	-1.66226200

C	-3.86960800	-1.36788900	-0.96852700
C	-5.38880000	0.63457600	-2.15612100
H	-3.73425900	1.94904200	-1.74474000
C	-5.13195500	-1.65818200	-1.47096200
H	-3.27057200	-2.14408800	-0.49909300
C	-5.89895500	-0.65740400	-2.06515500
H	-5.97588200	1.42035400	-2.62121200
H	-5.51478500	-2.67080300	-1.39340800
H	-6.88599200	-0.88417200	-2.45512800
C	-1.51095200	1.33744500	-0.28807700
O	-1.93657600	2.44840700	-0.42154200
N	-0.07861900	1.16732900	0.25922400
C	0.33715600	2.34455400	1.05538500
C	0.20433300	2.26844900	2.43566300
C	0.80562000	3.48226600	0.41135700
C	0.58014300	3.37017100	3.19969200
H	-0.19825300	1.37743600	2.90914400
C	1.17973200	4.57232700	1.19132900
H	0.87487300	3.52357600	-0.67050100
C	1.07080900	4.51678400	2.57970000
H	0.48459000	3.32721400	4.27937900
H	1.55205500	5.46876600	0.70706000
H	1.36405100	5.37228300	3.17945000
H	-1.53412200	-0.69862400	-0.33103500
H	-0.01429500	0.34319000	0.90512700

17

E (SMD/M06-2X/BS1) = -2568.62517784 au

G (SMD/M06-2X/BS1) = -2568.316688 au

E (SMD/M06-2X-D3/BS2//SMD/M06-2X/BS1) = -2569.19913799 au

N	-0.55239400	-1.65467900	-0.52364400
C	-1.84077500	-2.21297400	-0.78934200
C	-2.49972200	-2.88239000	0.23973800
C	-2.41086800	-2.09499400	-2.05651800
C	-3.75673300	-3.43124500	-0.00337700
H	-2.03507000	-2.95464200	1.21785400
C	-3.67569700	-2.63191700	-2.28183100
H	-1.87497700	-1.58326000	-2.84995200
C	-4.34976600	-3.29784200	-1.25850700
H	-4.27669600	-3.95062500	0.79495400
H	-4.13016800	-2.53351000	-3.26226200
H	-5.33493700	-3.71530400	-1.44005200

C	-0.22568600	-0.39702600	-0.76275800
O	-1.06023200	0.46656200	-1.21904500
N	1.04889900	0.07283800	-0.56957700
C	1.25892800	1.49047500	-0.79534500
C	1.68578700	1.92129100	-2.04767700
C	1.04536600	2.37944500	0.25321100
C	1.90720200	3.28130500	-2.25046100
H	1.83911900	1.19838300	-2.84348900
C	1.26378300	3.73710400	0.03668700
H	0.70471200	2.00726000	1.21482500
C	1.69531000	4.18558700	-1.21097900
H	2.23994900	3.63254400	-3.22174700
H	1.09408700	4.44289200	0.84318100
H	1.86533300	5.24512600	-1.37447300
H	0.16948600	-2.23345300	-0.08967400
C	2.11998700	-0.75674200	-0.12112500
O	1.94055400	-1.93315100	0.16025400
N	3.30568900	-0.11723400	-0.04151700
C	4.54478700	-0.67292100	0.35867800
C	5.68073100	0.09664600	0.08245900
C	4.67535400	-1.89843100	1.01832100
C	6.93907000	-0.35593600	0.45733700
H	5.56991500	1.05015800	-0.42784800
C	5.94618200	-2.33909600	1.38358400
H	3.80492000	-2.49706200	1.24465900
C	7.07997200	-1.58045600	1.10850200
H	7.81089100	0.25192100	0.23611500
H	6.04140600	-3.29188300	1.89554600
H	8.06272400	-1.93667600	1.40009200
H	3.35008200	0.84778900	-0.35054700
H	-2.07683900	0.33688100	-0.92719200
B	-3.37947700	1.03535100	0.91709100
Cl	-3.00188900	2.83761400	0.66730400
Cl	-5.03724600	0.78822600	1.74130100
Cl	-2.03098900	0.26365500	2.00642900
O	-3.38544500	0.40551100	-0.38754000
H	-3.80807400	-0.47186400	-0.35079000

18

E (SMD/M06-2X/BS1) = -1086.71140917 au

G (SMD/M06-2X/BS1) = -1086.427419 au

E (SMD/M06-2X-D3/BS2//SMD/M06-2X/BS1) = -1087.13145772 au

N	2.12749900	-0.53103000	0.01758900
C	3.51418800	-0.76924800	0.03430100
C	3.90699300	-2.11341000	-0.04014000
C	4.49282500	0.22792700	0.12688900
C	5.25186700	-2.45746200	-0.02301900
H	3.14515900	-2.88552200	-0.11293600
C	5.83880300	-0.13520100	0.14172500
H	4.20448800	1.26723700	0.18583200
C	6.23080500	-1.46836800	0.06777100
H	5.53361700	-3.50453200	-0.08187300
H	6.58882100	0.64719200	0.21406800
H	7.28283200	-1.73478100	0.08060400
C	1.50823500	0.67549500	0.00187100
O	2.07059800	1.75618100	0.00741400
N	0.07985400	0.65085700	-0.02210600
C	-0.53212700	1.95805000	-0.05287200
C	-0.79606200	2.62215300	1.14261900
C	-0.87370800	2.52454500	-1.27834700
C	-1.41037300	3.87150600	1.10813500
H	-0.51966900	2.15674100	2.08418400
C	-1.48830300	3.77396000	-1.30533300
H	-0.65715100	1.98432200	-2.19532600
C	-1.75608900	4.44643200	-0.11396200
H	-1.61763100	4.39512000	2.03603800
H	-1.75628400	4.22145000	-2.25717400
H	-2.23537900	5.42019100	-0.13779300
H	1.51638200	-1.34617800	-0.00805100
C	-0.72108100	-0.50203500	-0.02582900
O	-0.25198700	-1.63640200	-0.04060400
N	-2.06108700	-0.25681000	-0.01592700
C	-3.09498200	-1.21795300	0.01391200
C	-4.35543800	-0.76343700	0.42173300
C	-2.93292200	-2.55273700	-0.37251200
C	-5.43961100	-1.63143700	0.45007400
H	-4.47776700	0.27519900	0.71912100
C	-4.02932900	-3.41206100	-0.33191800
H	-1.96785200	-2.91267800	-0.69884800
C	-5.28272800	-2.96530400	0.07674900
H	-6.40901700	-1.26143700	0.76981900
H	-3.89341400	-4.44689600	-0.63204800
H	-6.12768300	-3.64596900	0.10213400
H	-2.36798700	0.70270300	0.08979700

6. References

[1] M. Santi, D. M. C. Ould, J. Wenz, Y. Soltani, R. L. Melen, T. Wirth, *Angew. Chem. Int. Ed*. **2019**, *58*, 7861–7865.

[2] R. K. Harris, E. D. Becker, S. M. Cabral De Menezes, R. Goodfellow, P. Granger, *Solid State Nucl Magn Reson*. **2002**, *4*, 458–483.

[3] A. J. South, A. M. Geer, L. J. Taylor, H. R. Sharpe, W. Lewis, A. J. Blake, D. L. Kays, *Organometallics* **2019**, *38*, 4115–4120.

[4] A. I. McKay, W. A. O. Altalhi, L. E. McInnes, M. L. Czyz, A. J. Canty, P. S. Donnelly, R. A. J. O'Hair, *J. Org. Chem.* **2020**, *85*, 2680–2687.

[5] R. Martínez, H. A. Jiménez-Vázquez, F. Delgado, J. Tamariz, *Tetrahedron* **2003**, *59*, 481–492.

[6] A. Kumar, N. Tadigoppula, *Org. Lett.* **2021**, *23*, 8–12.

[7] N. Busschaert, I. L. Kirby, S. Young, S. J. Coles, P. N. Horton, M. E. Light, P. A. Gale, *Angew. Chemie - Int. Ed.* **2012**, *51*, 4426–4430.

[8] N. Della Ca', P. Bottarelli, A. Dibenedetto, M. Aresta, B. Gabriele, G. Salerno, M. Costa, *J. Catal.* **2011**, *282*, 120–127.

[9] A. Ying, H. Hou, S. Liu, G. Chen, J. Yang, S. Xu, *ACS Sustain. Chem. Eng.* **2016**, *4*, 625–632.

[10] J. C. Anderson, R. B. Moreno, *Org. Biomol. Chem.* **2012**, *10*, 1334–1338.

[11] J. Lee, J. Lee, H. Jung, D. Kim, J. Park, S. Chang, *J. Am. Chem. Soc.* **2020**, *142*, 12324–12332.

[12] SHELXL-2013, G.M. Sheldrick, University of Göttingen, Germany (2013)

[13] CrysAlisPro, Agilent Technologies, Version 1.171.37.33 (release 27-03–2014 CrysAlis 171.NET)

[14] M. Soriano-García, S. Hernández-Ortega, A. E. Domínguez-Pérez, G. Trujillo-Chávez, F. Martínez-Ramos, *Analytical Sciences* **2001**, *17*, 355–356.

[15] S.-G. Roh, J. H. Jeong, *Acta Crystallographica Section C* **2000**, *56*, e529–e530.

[16] M. A. Solomos, T. A. Watts, J. A. Swift, *Crystal Growth & Design* **2017**, *17*, 5073–5079.

[17] M. J. Frisch, G. W. Trucks, H. B.Schlegel, G. E. Scuseria, M. A. Robb, J. R. Cheeseman, G.Scalmani, V. Barone, G. A. Petersson, H. Nakatsuji, X. Li, M. Caricato, A. V. Marenich, J. Bloino, B. G.Janesko, R.Gomperts, B.Mennucci, H. P.Hratchian, J. V.Ortiz, A. F.Izmaylov, J. L.Sonnenberg, D. Williams-Young, F. Ding, F. Lipparini, F. Egidi, J. Goings, B. Peng, A. Petrone, T. Henderson, D. Ranasinghe, V. G. Zakrzewski, J. Gao, N. Rega, G. Zheng, W. Liang, M. Hada, M. Ehara, K. Toyota, R. Fukuda, J. Hasegawa, M. Ishida, T. Nakajima, Y.

Honda, O. Kitao, H. Nakai, T. Vreven, K. Throssell, J. A. Montgomery, J. E. Peralta, F. Ogliaro, M. J. Bearpark, J. J. Heyd, E. N. Brothers, K. N. Kudin, V. N. Staroverov, T. A. Keith, R. Kobayashi, J. Normand, K. Raghavachari, A. P. Rendell, J. C. Burant, S. S. Iyengar, J. Tomasi, M. Cossi, J. M. Millam, M. Klene, C. Adamo, R. Cammi, J. W. Ochterski, R. L. Martin, K. Morokuma, O. Farkas, J. B. Foresman and D. J. Fox, Gaussian 16, Revision A.03, Gaussian, Inc., Wallingford CT, 2016.

[18] (a) Y. Zhao and D. G. Truhlar, *Theor. Chem. Acc.*, **2008**, *120*, 215–241; (b) Y. Zhao and D. G. Truhlar, *Acc. Chem. Res.*, **2008**, *41*, 157–167.

[19] A. V. Marenich, C. J. Cramer and D. G. Truhlar, *J. Phys. Chem. B*, **2009**, *113*, 6378–6396.

[20] P. C. Hariharan and J. A. Pople, *Theor. Chem. Acta.*, **1973**, *28*, 213–222.

[21] (a) K. Fukui, *J. Chem. Phys.*, **1970**, *74*, 4161-4163; (b) K. Fukui, *Acc. Chem. Res.*, **1981**, *14*, 363–368.

[22] F. Weigend, F. Furche and R. J. Ahlrichs, *J. Chem. Phys.*, **2003**, *119*, 12753–12762.

[23] (a) L. Goerigk, S. Grimme, *Physical Chemistry Chemical Physics* **2011**, *13*, 6670–6688; M. Modrzejewski, M. Hapka, G. Chalasinski, M. M. Szczesniak, *Journal of Chemical Theory and Computation* **2016**, *12*, 3662–3673.
